# Efficacy and safety of immunotherapy or antiangiogenic agent-based treatment strategies versus chemotherapy as first-line treatment for extensive-stage small cell lung cancer: a network meta-analysis

**DOI:** 10.3389/fphar.2025.1539246

**Published:** 2025-06-09

**Authors:** Chengjun Wang, Chuang Yang, Wen Zhao, Rongyu Zhang, Tiantian Xuan, Jisheng Li

**Affiliations:** ^1^ Department of Medical Oncology, Qilu Hospital, Cheeloo College of Medicine, Shandong University, Jinan, Shandong, China; ^2^ Department of Medical Oncology, Qilu Hospital (Qingdao), Cheeloo College of Medicine, Shandong University, Qingdao, Shandong, China

**Keywords:** small cell lung cancer, immunotherapy, antiangiogenesis, network meta-analysis, chemotherapy

## Abstract

**Objective:**

Immune checkpoint inhibitors (ICIs) combined with etoposide-platinum are recommended as the standard first-line therapy for extensive-stage small cell lung cancer (ES-SCLC). Despite the potential of antiangiogenic agents to enhance treatment efficacy, the optimal combination pattern remains unclear. This meta-analysis explores existing treatment strategies involving ICIs or antiangiogenic agents in ES-SCLC.

**Methods:**

Hazard ratios (HRs) and odds ratios (ORs) were generated by R software. The outcomes of overall survival (OS), progression-free survival (PFS), objective response rate (ORR), and adverse events of grade 3 or higher (Grade ≥3 AEs) were analyzed. The included trials were classified in terms of different treatment strategies, including ICI + Chemotherapy (ICI + Chemo), ICI + ICI + Chemotherapy (ICI + ICI + Chemo), ICI + Antiangiogenic agent + Chemotherapy (ICI + Antiangio + Chemo), Antiangiogenic agent + Chemotherapy (Antiangio + Chemo), and Chemotherapy (Chemo).

**Results:**

A total of 13 randomized controlled trials (RCTs) involving 6,822 patients were included in the analysis. The drug combination patterns included ipilimumab, durvalumab, adebrelimab, atezolizumab, socazolimab, pembrolizumab, serplulimab, tislelizumab, toripalimab, durvalumab + tremelimumab, tiragolumab + atezolizumab, benmelstobart + anlotinib, bevacizumab + atezolizumab, anlotinib, bevacizumab in combination with chemotherapy. The antiangiogenic agent-containing regimen benmelstobart + anlotinib + chemotherapy demonstrated the highest potential to achieve superior PFS and OS versus chemotherapy. The group meta-analysis also showed that ICI + Chemo, ICI + ICI + Chemo, and ICI + Antiangio + Chemo presented significantly better OS. Additionally, ICI + Antiangio + Chemo achieved better PFS with the lowest HR of 0.37 and the best ORR of 2.08 versus chemotherapy. Patients treated with benmelstobart + anlotinib + chemotherapy, durvalumab + tremelimumab + chemotherapy, and anlotinib + chemotherapy experienced a higher likelihood of grade ≥3 AEs.

**Conclusion:**

For individuals with ES-SCLC, ICI + Antiangio + Chemo was identified as an optimal treatment option due to better OS, PFS, and ORR. Benmelstobart + anlotinib + chemotherapy demonstrated a better survival benefit than chemotherapy. The toxicity of ICI + Antiangio + Chemo was acceptable but needed careful attention. These findings clarified the roles of ICIs and antiangiogenic agent-based treatment strategies in this population.

## 1 Introduction

Lung cancer was the most frequently diagnosed cancer in 2022, responsible for almost 2.5 million new cases or one in eight cancers worldwide (12.4% of all cancers globally), and is also the leading cause of cancer death with an estimated 1.8 million deaths (18.7%) (Bray et al., 2024). Small cell lung cancer (SCLC) accounts for 10%–15% of all lung carcinoma diagnoses and is characterized by rapid development of treatment resistance and high recurrence rates (Micke et al., 2002; Torre et al., 2016; Oronsky et al., 2017). This neoplasm is marked by aggressive growth, rapid progression, and a high propensity for metastasis, with more than two-thirds of patients presenting with extensive-stage SCLC (ES-SCLC) (Micke et al., 2002; Torre et al., 2016; Oronsky et al., 2017). SCLC is often associated with a poor prognosis, as evidenced by a 5-year survival rate of only 7% (Gazdar and Minna, 2016; Torre et al., 2016; Siegel et al., 2022; García-Campelo et al., 2023). For decades, the standard first-line treatment for ES-SCLC has been platinum-based chemotherapy with etoposide or irinotecan (Jackman and Johnson, 2005; Jalal et al., 2017; Ganti et al., 2021). However, due to the rapid development of resistance, the transient benefit of therapy, and the limited effectiveness of subsequent treatments, the survival outcomes remained poor, with a median overall survival (mOS) of approximately 10 months (Jackman and Johnson, 2005; Rossi et al., 2012; Jalal et al., 2017; Wang et al., 2019). This ongoing challenge highlights the urgent necessity for further research to develop novel therapeutic strategies aimed at improving patient prognosis and prolonging survival.

In recent years, the emergence of immunotherapy, particularly immune checkpoint inhibitors (ICIs) targeting the programmed cell death-(ligand) 1 (PD-1/PD-L1) pathway, has transformed the treatment landscape and significantly improved the prognosis of ES-SCLC. Numerous clinical studies have demonstrated substantial enhancements in overall survival (OS) and progression-free survival (PFS) (Goldman et al., 2021; Liu et al., 2021; Cheng Y. et al., 2022; Wang et al., 2022). For instance, the IMpower133 trial showed that the combination of atezolizumab with carboplatin and etoposide significantly improved mOS in patients with ES-SCLC (Liu et al., 2021). The CASPIAN trial is another randomized phase III trial that evaluated durvalumab with etoposide-platinum in comparison to etoposide-platinum as first-line therapy and proved the addition of durvalumab significantly improved OS (Paz-Ares et al., 2019; Goldman et al., 2021). Based on these findings, atezolizumab and durvalumab combined with chemotherapy are recommended as first-line therapeutic options for small cell lung cancer. Then, several ICIs, such as pembrolizumab (Rudin et al., 2020), ipilimumab (Reck et al., 2016), tremelimumab (Goldman et al., 2021), adebrelimab (Wang et al., 2022), serplulimab (Cheng Y. et al., 2022), tislelizumab (Cheng et al., 2024b), and toripalimab (Cheng et al., 2024c), were also investigated in the first-line treatment of ES-SCLC.

Vascular endothelial growth factor (VEGF) is over-expressed in SCLC and is associated with poor prognosis (Montanino et al., 2021). Previous studies have confirmed angiogenesis plays a fundamental role in SCLC growth and spread, and it has been involved in the development of chemotherapy resistance (Stratigos et al., 2016; Caliman et al., 2023). Antiangiogenic therapies, such as bevacizumab, are also transforming the treatment landscape for SCLC. Bevacizumab, an anti-VEGF antibody, impedes tumor growth by inhibiting the formation of new blood vessels (Stratigos et al., 2016). Clinical studies, such as BEAT-SC, have demonstrated that incorporating bevacizumab into standard chemotherapy regimens can improve the therapeutic effect to a certain extent (Spigel et al., 2011; Tiseo et al., 2017; Ohe et al., 2024). Positive results have been observed in ETER701, which explored the efficacy and safety of the combination of a PD-L1 antibody, benmelstobart, an antiangiogenic agent, anlotinib, and chemotherapy (Cheng et al., 2024a). This approach addresses tumor vascularity and resistance, offering new avenues for treatment. It is worth exploring their positive synergistic effects in chemoimmunotherapy.

Current randomized clinical trials do not conduct direct head-to-head comparisons between different combination treatments. Furthermore, previous meta-analyses have only partially compared various chemoimmunotherapy regimens, excluding antiangiogenic agents (Zhou et al., 2020; Zhang et al., 2023). Therefore, our meta-analysis aims to evaluate recent phase III immunotherapy or antiangiogenic agent-based clinical trial data, classifying them based on different treatment strategies and focusing on their impact on OS, PFS, objective response rate (ORR), and adverse events of grade 3 or higher (Grade ≥3 AEs). By analyzing these factors, the study seeks to optimize treatment strategies, advance clinical practice, enhance patient prognosis, and support evidence-based decision-making in the management of ES-SCLC.

## 2 Methods

We followed the Preferred Reporting Items for Systemic Reviews and Meta-analyses (PRISMA) checklist when conducting this meta-analysis. The network meta-analysis (NMA) was conducted and reported in accordance with the PRISMA Extension version (PRISMA-NMA) (Supplementary Table S1). This study protocol has been duly registered on the International Prospective Register of Systematic Reviews (PROSPERO) under the registration number CRD42024555325.

### 2.1 Retrieval method

We searched PubMed, Embase, Web of Science, and ClinicalTrials.gov using the following terms: small cell lung carcinoma, extensive-stage, immunotherapy, immune checkpoint inhibitor, tremelimumab, nivolumab, pembrolizumab, atezolizumab, adebrelimab, ipilimumab, durvalumab, serplulimab, tislelizumab, benmelstobart, toripalimab, socazolimab, bevacizumab, anlotinib, angiogenesis, and randomized controlled trial, as well as their related MeSH terms. Additionally, major international conferences were searched for phase III randomized controlled trials (RCTs) regarding comparing immunotherapy or antiangiogenic agent-based combinations with chemotherapy as first-line treatments for patients with ES-SCLC from 2016 to 2024. The detailed search strategy is shown in Supplementary Table S2.

### 2.2 Inclusion and exclusion criteria

Studies were included if they (1) were prospective, randomized, phase III, and controlled clinical trials; (2) enrolled patients with either histologically or cytologically confirmed ES-SCLC who had not yet received any treatment; (3) compared any two or more different arms of treatments for patients with ES-SCLC; and (4) were based on immunotherapy or antiangiogenic-agent treatment strategies in the intervention arms. The exclusion criteria were as follows: (1) reviews, case reports, meta-analyses, or letters; (2) retrospective study, phase I or II clinical trials, single-arm studies, or observational studies; (3) studies without complete survival data or with data unavailable.

### 2.3 Data extraction and quality assessment

The primary outcome was the OS, and the secondary outcomes included PFS, ORR, and grade ≥3 AEs. Two researchers conducted independent reviews of articles and extracted data from each eligible study. The extracted information included first author, year of publication, study title, sample size, study phase, treatment regimens, intervention, and patient characteristics, including age and gender, smoking status, Eastern Cooperative Oncology Group performance status (ECOG PS), brain metastases, and liver metastases. The clinical outcomes extracted included hazard ratios (HRs) and a median with corresponding 95% confidence intervals (CIs) for OS (randomization to death regardless of any causes) and PFS (randomization to the progression of any causes or death irrespective of any causes), and dichotomous data for ORR and AEs. Two other investigators assessed the risk of bias in the included studies by using Review Manager 5.4 software. Any disagreements were resolved through discussions, and consensus was reached.

### 2.4 Statistical analysis

We performed network meta-analysis with R software (version 4.3.1) (R Project for Statistical Computing with the gemtc package) using Bayesian fixed-effect consistency models to be fitted for multiple comparisons of different treatments for SCLC. As for Rstudio, we ran 100,000 simulations, using the first 50,000 as the burn-in period. At the same time, we ranked the likelihood of different treatment options based on cumulative ranking probabilities to present pairwise comparisons between regimens for OS, PFS, ORR, and grade ≥3 AEs. The software can calculate the likelihood of each intervention being ranked as the top choice. The regimens in these included trials, except for ipilimumab (anti-cytotoxic T-lymphocyte antigen 4 antibody, anti-CTLA4) + chemotherapy, were classified into five different treatment strategies: ICI + Chemotherapy (ICI + Chemo), ICI + ICI + Chemotherapy (ICI + ICI + Chemo), ICI + Antiangiogenic agent + Chemotherapy (ICI + Antiangio + Chemo), Antiangiogenic agent + Chemotherapy (Antiangio + Chemo), and Chemotherapy alone (Chemo) to conduct meta-analysis in groups. Hazard ratios (HRs) for survival outcomes (PFS and OS) and odds ratios (ORs) for binary outcomes (ORR and grade ≥3 AEs) were calculated, along with their 95%CIs. The fixed-effect model was adopted for OS analysis; the random-effects model was employed for other analyses to account for potential heterogeneity. Statistical heterogeneity among studies was assessed using the I^2^ statistic and Q-test. Results were presented as conventional meta-analysis forest plots using Review Manager 5.4 software. A p < 0.05 was considered a significant inconsistency. Chuang Yang and Chengjun Wang made the major contributions to the statistical analysis.

## 3 Results

### 3.1 Systematic review and baseline characteristics

**FIGURE 1:**
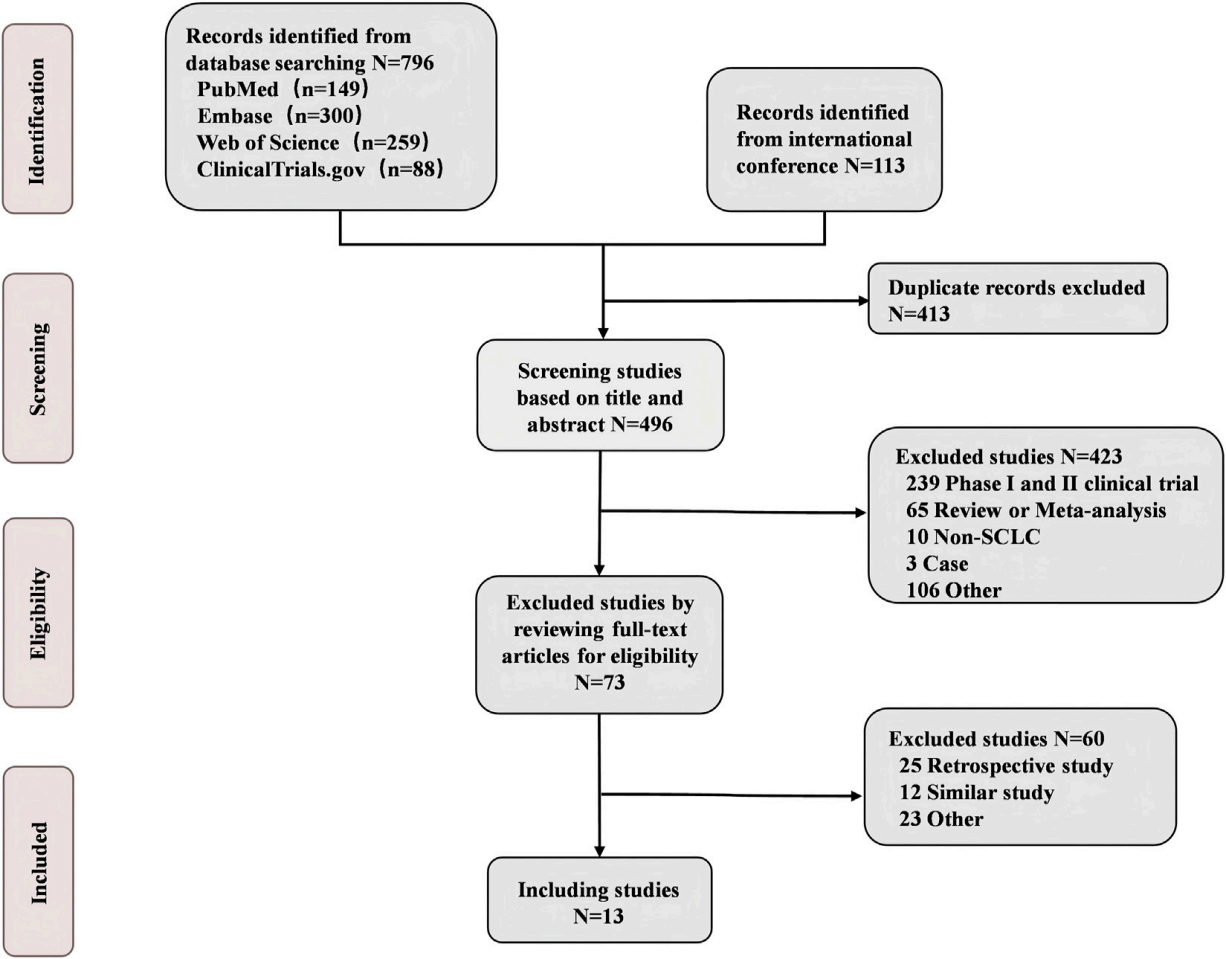
Flow diagram of eligible studies in our analysis.

A total of 13 eligible phase III RCTs were included in our study, involving 6822 ES-SCLC patients who received chemotherapy with or without immunotherapy and antiangiogenic agents (Figure 1; Table 1). Among the 13 trials, the intervention arms involved diverse regimens, including ipilimumab + chemotherapy (Ipi + Chemo), durvalumab + chemotherapy (Dur + Chemo), adebrelimab + chemotherapy (Ade + Chemo), atezolizumab + chemotherapy (Ate + Chemo), pembrolizumab + chemotherapy (Pem + Chemo), serplulimab + chemotherapy (Ser + Chemo), tislelizumab + chemotherapy (Tis + Chemo), toripalimab + chemotherapy (Tor + Chemo), durvalumab + tremelimumab + chemotherapy (Dur + Tre + Chemo), tiragolumab + atezolizumab + chemotherapy (Tir + Ate + Chemo), benmelstobart + anlotinib + chemotherapy (Ben + Anl + Chemo), bevacizumab + atezolizumab + chemotherapy (Bev + Ate + Chemo), anlotinib + chemotherapy (Anl + Chemo), and bevacizumab + chemotherapy (Bev + Chemo).

**TABLE 1:** The baseline characteristics of included trails.

No.	Study	Author	Year	Intervention arm	No. of patients	Control arm	No. of patients 2	OS (HR, 95%CI)	PFS (HR, 95%CI)
1	Reck et al.	Reck et al.	2016	Ipilimumab plus EP	478	Placebo plus EP	476	0.94 (0.81, 1.09)	0.85 (0.75, 0.97)
2	GORIC-AIFA	Tiseo et al.	2017	Bevacizumab plus EC	101	EC	103	0.78 (0.58, 1.06)	0.72 (0.54, 0.97)
3	KEYNOTE-604	Rudin et al.	2020	Pembrolizumab plus EP	228	Placebo plus EP	225	0.80 (0.64, 0.98)	0.75 (0.61, 0.91)
4	CASPIAN	Goldman et al.	2021	Durvalumab plus EP, durvalumab plus tremelimumab plus EP	268 268	EP	269	0.75 (0.62, 0.91) 0.82 (0.68, 1.00)	0.80 (0.66, 0.96) 0.84 (0.70, 1.01)
5	CAPSTONE-1	Wang et al.	2022	Adebrelimab plus chemotherapy	230	Placebo plus chemotherapy	232	0.72 (0.58, 0.90)	0.67 (0.54, 0.83)
6	IMpower133	Stephen et al.	2022	Atezolizumab plus CP/ET	201	Placebo Plus CP/ET	202	0.76 (0.60, 0.95)	0.77 (0.63, 0.95)
7	ASTRUM-005	Cheng et al.	2022	Serplulimab plus chemotherapy	389	Placebo plus chemotherapy	196	0.63 (0.49, 0.82)	0.48 (0.38, 0.59)
8	SKYSCRAPER-02	Rudin et al.	2022	Tiragolumab plus atezolizumab plus EC	243	Atezolizumab plus EC	247	1.09 (0.88, 1.35)	1.08 (0.89, 1.31)
9	ETER701	Cheng et al.	2023	Benmelstobart plus anlotinib plus EC, placebo plus anlotinib plus EC	246 245	Placebo plus Placebo plus EC	247	0.61 (0.46, 0.79) 0.86 (0.67.1.10)	0.32 (0.26, 0.41) 0.44 (0.36, 0.55)
10	RATIONALE-312	Cheng et al.	2023	Tislelizumab plus EC	227	Placebo plus EC	230	0.75 (0.61, 0.92)	0.63 (0.51, 0.78)
11	EXTENTORCH	Cheng et al.	2023	Toripalimab plus EP	223	Placebo plus EP	219	0.80 (0.65, 0.98)	0.67 (0.54, 0.82)
12	BEAT-SC	Yuichiro Ohe et al.	2024	Bevacizumab plus atezolizumab plus EP/EC	167	Placebo plus atezolizumab plus EP/EC	166	1.22 (0.89, 1.67)	0.70 (0.54, 0.90)
13	NCT04878016	Shun Lu et al.	2024	Socazolimab plus EC	248	Placebo plus EC	248	0.80 (0.65,0.98)	0.57 (0.46,0.71)

Abbreviations: CI, confidence interval; EC, etoposide plus carboplatin; EP, etoposide plus cisplatin/carboplatin; CP/ET, etoposide plus carboplatin; HR, hazard ratio; No., number; OS, overall survival; PFS, progression-free survival.

We conducted a comprehensive analysis of the demographic information and disease characteristics of patients enrolled, with details provided in Supplementary Table S3. The analysis revealed that most participants were under the age of 65, with male patients typically accounting for more than 60%. A significant proportion of patients were smokers. There were significant differences in the ECOG PS scores among patients across different trials. The rate of patients with brain metastases was lower than those with liver metastases, and the rates of both liver and brain metastases varied among different studies.

These trials, except for ipilimumab + chemotherapy (Reck et al., 2016), were classified into five different treatment strategies to conduct the meta-analysis in groups: ICI + Chemo, ICI + ICI + Chemo, ICI + Antiangio + Chemo, Antiangio + Chemo, and Chemo. We ruled out ipilimumab in the group meta-analysis because it is an anti-CTLA4 monoclonal antibody and demonstrated no benefit to OS (HR = 0.94, 95%CI: 0.81–1.09) compared with chemotherapy alone. Detailed information on all the included studies is presented in Table 1. The risk of bias assessment of the 13 included trials conducted independently by two investigators (Chuang Yang and Chengjun Wang) is shown in Supplementary Figure S1 and suggests a low risk of bias. The trace and density plots are shown in Supplementary Figures S2, S6.

### 3.2 Results in the network meta-analysis

**FIGURE 2:**
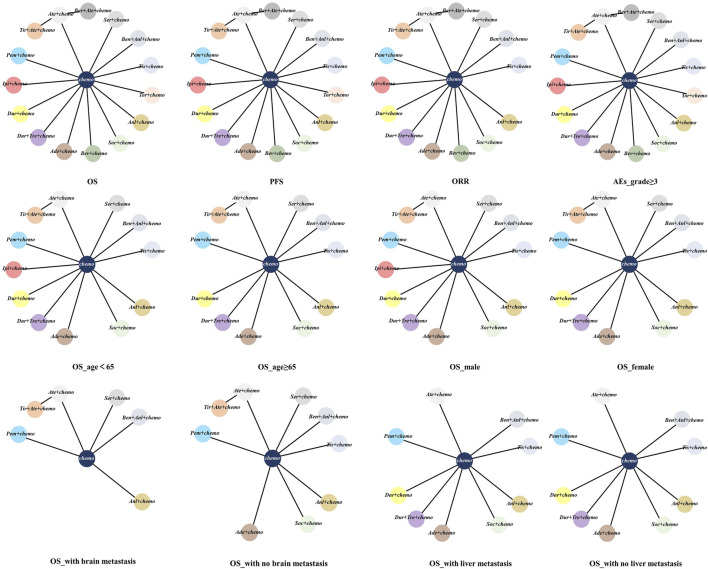
Network meta-analysis of comparisons of different outcomes of first-line treatments in various groups of ES-SCLC patients. Each circle represents a treatment, and the line between the two points represents a comparison between the two treatments. Chemo, chemotherapy; OS, overall survival; PFS, progression-free survival; ORR, objective response rate; AEs, adverse events. Ade + chemo, adebrelimab + chemotherapy; Ate + chemo, atezolizumab + chemotherapy; Dur + Tre + chemo, durvalumab + tremelimuamb + chemotherapy; Dur + chemo, durvalumab + chemotherapy; Ipi + chemo, ipilimumab + chemotherapy; Pem + chemo, pembrolizumab + chemotherapy; Ser + chemo, serplulimab + chemotherapy; Ben + Anl + chemo, benmelstobart + anlotinib + chemotherapy; Tis + chemo, tislelizumab + chemotherapy; Tir + Ate + chemo, tiragolumab + atezolizumab + chemotherapy; Tor + chemo, toripalimab + chemotherapy; Anl + chemo, anlotinib + chemotherapy; Bev + Ate + chemo, bevacizumab + atezolizumab + chemotherapy; Soc + chemo, socazolimab + chemotherapy; Bev + chemo, bevacizumab + chemotherapy; Chemo, chemotherapy.

**FIGURE 3:**
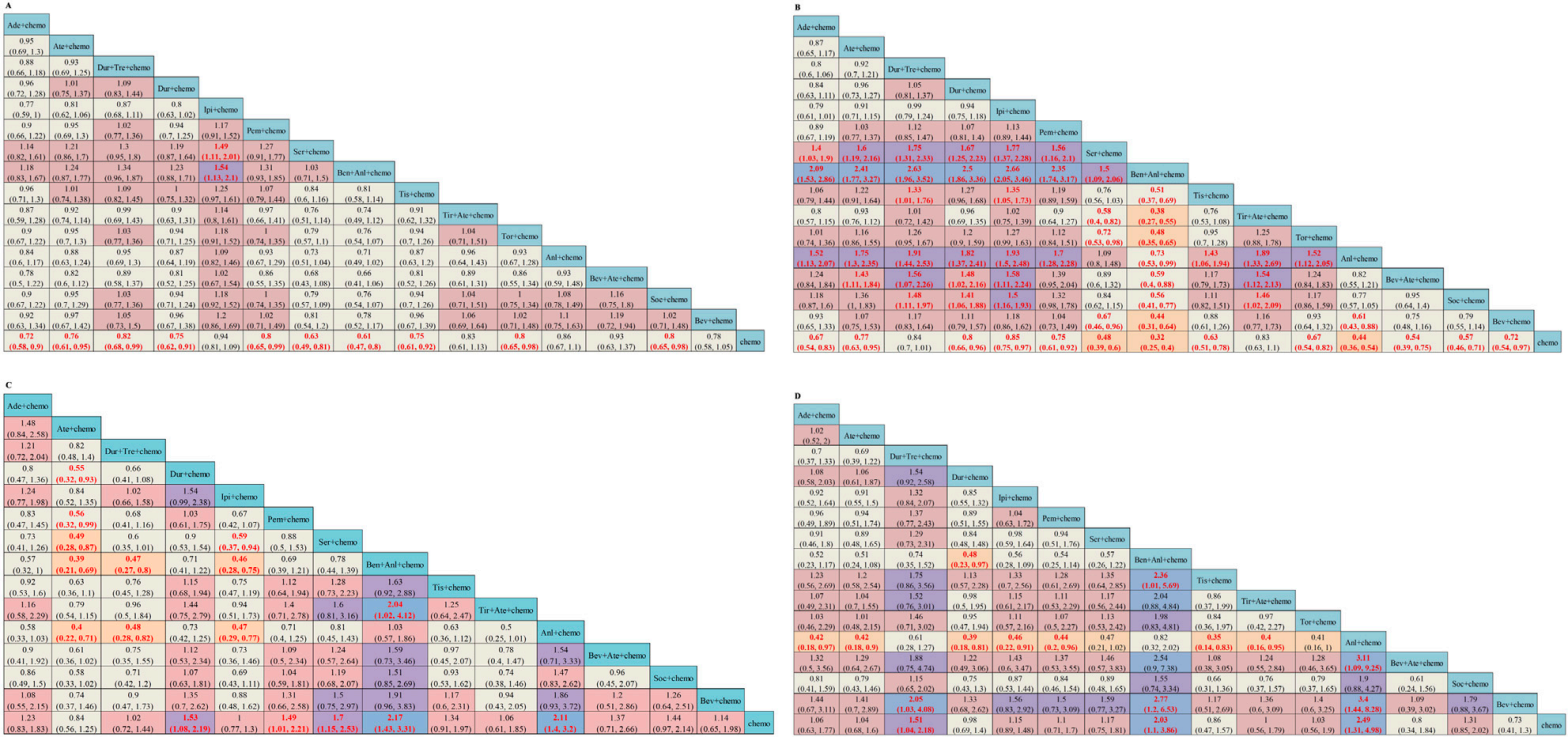
Efficacy and safety summaries from Bayesian network meta-analysis in ES-SCLC patients: **(A)** OS, **(B)** PFS, **(C)** ORR, and **(D)** AEs. The data in each cell represent the HR or OR values that compare treatment effects as defined by the columns and rows. The cells are color-coded according to the HR or OR values: those with HR or OR below 0.5 are highlighted in orange, those ranging from 0.5 to 1 in tan, from 1 to 1.5 in light red, from 1.5 to 2 in purple, and those exceeding 2 in blue. The important HRs (or ORs) and 95% confidence interval are highlighted in red and bold. OS, overall survival; PFS, progression-free survival; ORR, objective response rate; AE, adverse event; ICIs, immune checkpoint inhibitors; HR, Hazard ratio; OR, Odds ratio; CI, confidence interval; Ade + chemo, adebrelimab + chemotherapy; Ate + chemo, atezolizumab + chemotherapy; Dur + Tre + chemo, durvalumab + tremelimuamb + chemotherapy; Dur + chemo, durvalumab + chemotherapy; Ipi + chemo, ipilimumab + chemotherapy; Pem + chemo, pembrolizumab + chemotherapy; Ser + chemo, serplulimab + chemotherapy; Ben + Anl + chemo, benmelstobart + anlotinib + chemotherapy; Tis + chemo, tislelizumab + chemotherapy; Tir + Ate + chemo, tiragolumab + atezolizumab + chemotherapy; Tor + chemo, toripalimab + chemotherapy; Anl + chemo, anlotinib + chemotherapy; Bev + Ate + chemo, bevacizumab + atezolizumab + chemotherapy; Soc + chemo, socazolimab + chemotherapy; Bev + chemo, bevacizumab + chemotherapy; Chemo, chemotherapy.

The network was structured to facilitate multiple comparisons between different combination regimens with chemotherapy alone (Figure 2). From the NMA results in Figure 3A, benmelstobart + anlotinib + chemotherapy (HR = 0.61, 95%CI: 0.47–0.80) showed a statistically significantly better OS than chemotherapy alone. Adebrelimab (HR = 0.72, 95%CI: 0.58–0.90), atezolizumab (HR = 0.76, 95%CI: 0.61–0.95), durvalumab + tremelimumab (HR = 0.82, 95%CI: 0.68–0.99), durvalumab (HR = 0.75, 95%CI: 0.62–0.91), pembrolizumab (HR = 0.80, 95%CI: 0.65–0.99), serplulimab (HR = 0.63, 95%CI: 0.49–0.81), tislelizumab (HR = 0.75, 95%CI: 0.61–0.92), toripalimab (HR = 0.80, 95%CI: 0.65–0.98), and socazolimab (HR = 0.80, 95%CI: 0.65–0.98) combined with chemotherapy also achieved better overall survival than chemotherapy alone.

The OS rate ORs at the third, sixth, ninth, 12th, 15th, 18th, 21st, and 24th months were examined to analyze the OS for different ICI-based regimen combinations compared to standard chemotherapy (Supplementary Table S4). Compared with chemotherapy, only Ser + chemo (OR = 2.29, 95%CI: 1.40–3.75) significantly increased the sixth month OS rate. At the 12th month, Ade + chemo (OR = 1.49, 95%CI: 1.03–2.17), Ate + chemo (OR = 1.67, 95%CI: 1.12–2.48), Dur + chemo (OR = 1.74, 95%CI: 1.23–2.45), Ser + chemo (OR = 1.60, 95%CI: 1.12–2.28), and Ben + Anl + chemo (OR = 1.74, 95%CI: 1.21–2.50) all presented significantly better OS benefit than chemotherapy. Ade + chemo (OR = 2.25, 95%CI: 1.44–3.54), Dur + Tre + chemo (OR = 1.73, 95%CI: 1.12–2.70), Dur + chemo (OR = 1.71, 95%CI: 1.09–2.68), Pem + chemo (OR = 2.31, 95%CI: 1.38–3.97), Ben + Anl + chemo (OR = 2.25, 95%CI: 1.53–3.34), Tis + chemo (OR = 1.78, 95%CI: 1.17–2.73), and Soc + chemo (OR = 4.19, 95%CI: 2.33–7.98) all significantly increased the 24th month OS rate compared with chemotherapy alone.

We found that Tor + chemo and Bev + chemo showed no significant difference in efficacy at any time point. A significant advantage was observed with Ben + Anl + chemo compared with chemo alone, as summarized based on a matrix plot of each comparison of all regimens on the efficacy across all regimens from the 3rd to the 24th months (Supplementary Table S5). As for the rank-heat plot of OS, each sector was colored based on the surface under the cumulative ranking value of the corresponding treatment and outcome for each month. Ben + Anl + chemo was a first-echelon regimen and achieved the top ranking in terms of best survival benefit among the other regimens in the 3rd to 24th month (Supplementary Figure S3A).

Regarding PFS, according to the results in Figure 3B, our NMA indicated that benmelstobart + anlotinib + chemotherapy showed the best efficacy among all combinations (HR = 0.32, 95%CI: 0.25–0.40) compared to chemotherapy. It also demonstrated the considerable advantage of better PFS than all other combination regimens, including Tis + chemo (HR = 0.51, 95%CI: 0.37–0.69), Tir + Ate + chemo (HR = 0.38, 95%CI: 0.27–0.55), Tor + chemo (HR = 0.48, 95%CI: 0.35–0.65), Anl + chemo (HR = 0.73, 95%CI: 0.53–0.99), Bev + Ate + chemo (HR = 0.59, 95%CI: 0.40–0.88), Soc + chemo (HR = 0.56, 95%CI: 0.41–0.77), and Bev + chemo (HR = 0.44, 95%CI: 0.31–0.64). In terms of PFS improvement, Ade + chemo (HR = 2.09, 95%CI: 1.53–2.86), Ate + chemo (HR = 2.41, 95%CI: 1.77–3.27), Dur + Tre + chemo (HR = 2.63, 95%CI: 1.96–3.52), Dur + chemo (HR = 2.50, 95%CI: 1.86–3.36), Ipi + chemo (HR = 2.66, 95%CI: 2.05–3.46), Pem + chemo (HR = 2.35, 95%CI: 1.74–3.17), and Ser + chemo (HR = 1.50, 95%CI: 1.09–2.06) were also inferior to Ben + Anl + chemo. At the second month (Supplementary Table S6), only Soc + chemo (OR = 2.76, 95%CI: 1.17–7.33) increased PFS compared with chemotherapy. At the sixth month, Ade + chemo (OR = 1.64, 95%CI: 1.13–2.38), Pem + chemo (OR = 1.69, 95%CI: 1.12–2.56), Ser + chemo (OR = 2.60, 95%CI: 1.82–3.72), Tis + chemo (OR = 2.65, 95%CI: 1.72–4.13), Ben + Anl + chemo (OR = 6.22, 95%CI: 3.98–10.00), Anl + chemo (OR = 4.93, 95%CI: 3.26–7.59), and Soc + chemo (OR = 2.57, 95%CI: 1.74–3.80) demonstrated improved PFS rate. From the eighth month to the 12th month, all regimens, with the exception of Ate + chemo, Dur + Tre + chemo, Ipi + chemo, and Bev + chemo, improved the PFS rate. Bev + chemo failed to show statistical superiority in PFS rates at any month. These data are summarized based on a matrix plot of each pairwise comparison of the efficacy of all regimens from the first to the 12th months (Supplementary Table S7). The rank-heat plot shows that Ben + Anl + chemo demonstrated the highest potential in improving PFS, followed by Soc + chemo (Supplementary Figure S3B).

Regarding the ORR of 12 studies (Tor + chemo is not included), compared with chemotherapy, Dur + chemo (OR = 1.53, 95%CI: 1.08–2.19), Pem + chemo (OR = 1.49, 95%CI: 1.01–2.21), Ser + chemo (OR = 1.70, 95%CI: 1.15–2.53), Ben + Anl + chemo (OR = 2.17, 95%CI: 1.43–3.31), and Anl + chemo (OR = 2.11, 95%CI: 1.40–3.20) significantly increased ORR (Figure 3C).

To compare the safety of various treatments, we calculated ORs for AEs of grade≥3 in 13 studies. Compared with chemotherapy, Dur + Tre + chemo (OR = 1.51, 95%CI: 1.04–2.18), Ben + Anl + chemo (OR = 2.03, 95%CI: 1.10–3.86), and Anl + chemo (OR = 2.49, 95%CI: 1.31–4.98) caused more grade ≥3 AEs. The details of the results are shown in Figure 3D. Additionally, the commonly reported adverse events of grade ≥3 associated with combined immunotherapy are presented in Supplementary Table S8.

### 3.3 Subgroup analysis of OS

**FIGURE 4:**
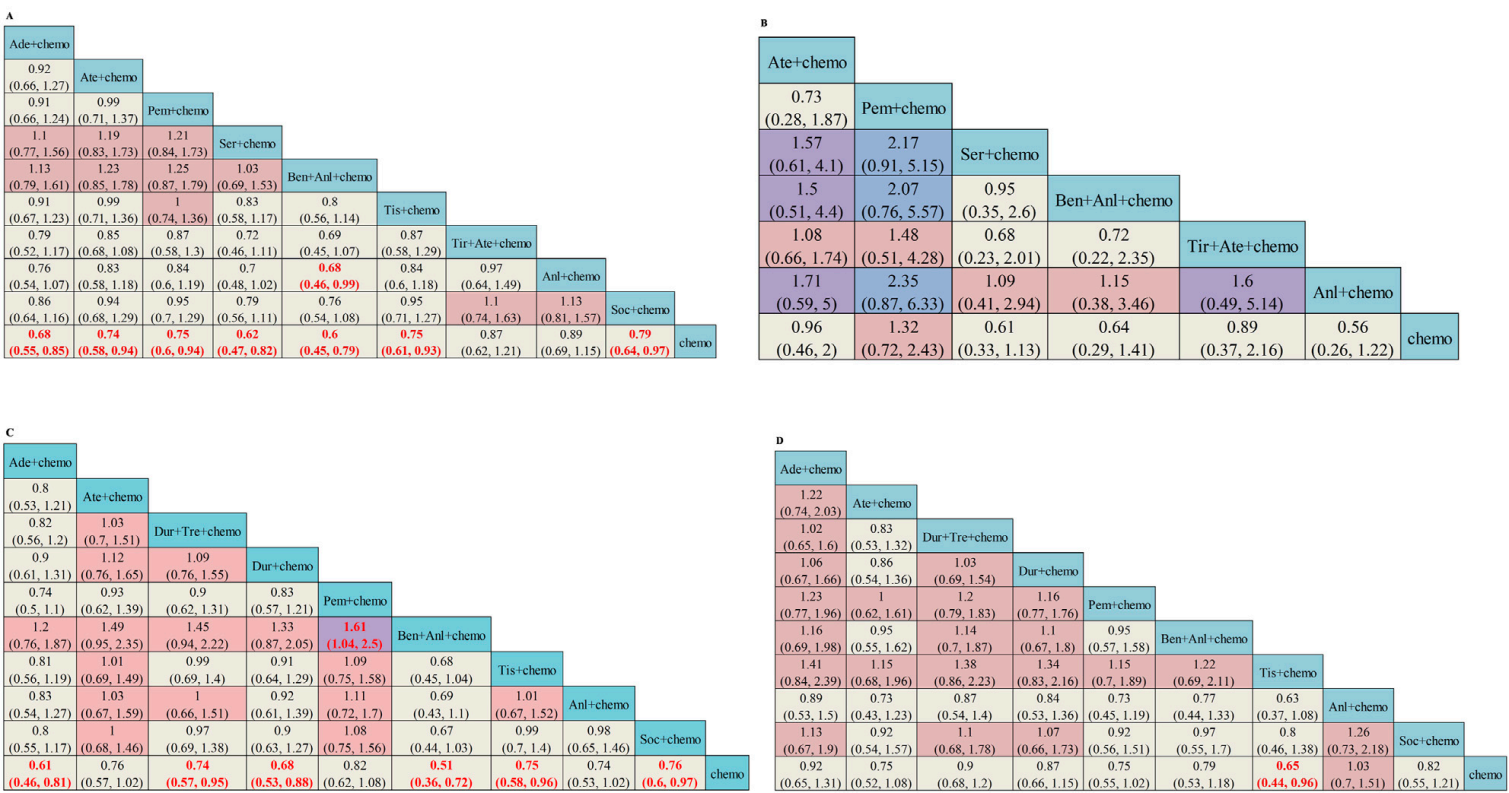
Subgroup analysis of OS from Bayesian network meta-analysis in ES-SCLC patients: **(A)** Pooled HRs and 95%CI in patients with no brain metastasis, **(B)** Pooled HRs and 95%CI in patients with brain metastasis, **(C)** Pooled HRs and 95%CI in patients with no liver metastasis, and **(D)** Pooled HRs and 95%CI in patients with liver metastasis. The data in each cell represent the HR values that compare treatment effects as defined by the columns and rows. The cells are color-coded according to the HR values: those with HR below 0.5 are highlighted in orange, those ranging from 0.5 to 1 in tan, from 1 to 1.5 in light red, from 1.5 to 2 in purple, and those exceeding 2 in blue. The important results are highlighted in red and bold. HRs, hazard ratios; CI, confidence interval; y, years; Ade + chemo, adebrelimab + chemotherapy; Ate + chemo, atezolizumab + chemotherapy; Dur + Tre + chemo, durvalumab + tremelimuamb + chemotherapy; Dur + chemo, durvalumab + chemotherapy; Ipi + chemo, ipilimumab + chemotherapy; Pem + chemo, pembrolizumab + chemotherapy; Ser + chemo, serplulimab + chemotherapy; Ben + Anl + chemo, benmelstobart + anlotinib + chemotherapy; Tis + chemo, tislelizumab + chemotherapy; Tir + Ate + chemo, tiragolumab + atezolizumab + chemotherapy; Tor + chemo, toripalimab + chemotherapy; Anl + chemo, anlotinib + chemotherapy; Bev + Ate + chemo, bevacizumab + atezolizumab + chemotherapy; Soc + chemo, socazolimab + chemotherapy; Bev + chemo, bevacizumab + chemotherapy; Chemo, chemotherapy.

The subgroup analysis of OS was stratified by age, gender, and the presence of liver metastases or brain metastases. In patients without brain metastasis, except for Tir + Ate + chemo (HR = 0.87, 95%CI: 0.62–1.21) and Anl + chemo (HR = 0.89, 95%CI: 0.69–1.15), other ICI-based regimens all demonstrated statistically significantly superior OS versus chemo (Figure 4A). Subgroup survival data for patients with brain metastasis are available in six studies. In patients with brain metastasis, no regimens showed a significantly superior OS versus chemo (Figure 4B). Subgroup survival data for patients with or without liver metastasis are available in eight studies. In patients without liver metastasis, Ate + chemo (HR = 0.76, 95%CI: 0.57–1.02), Pem + chemo (HR = 0.82, 95%CI: 0.62–1.08), and Anl + chemo (HR = 0.74, 95%CI: 0.53–1.02) failed to demonstrate OS superiority versus chemotherapy (Figure 4C). For patients with liver metastases, only Tis + chemo (HR = 0.65, 95%CI: 0.44–0.96) presented better efficacy than chemotherapy (Figure 4D). Both Ser + chemo and Ben + Anl + chemo significantly improved OS versus chemo with better HR in patients under the age of 65 and aged 65 years or older (Supplementary Figure S4A, S4B). Among all ICI-based regimens, benmelstobart + anlotinib + chemotherapy was associated with the best OS HR in both genders (Supplementary Figure S4C, S4D).

### 3.4 Results in the group meta-analysis

**FIGURE 5:**
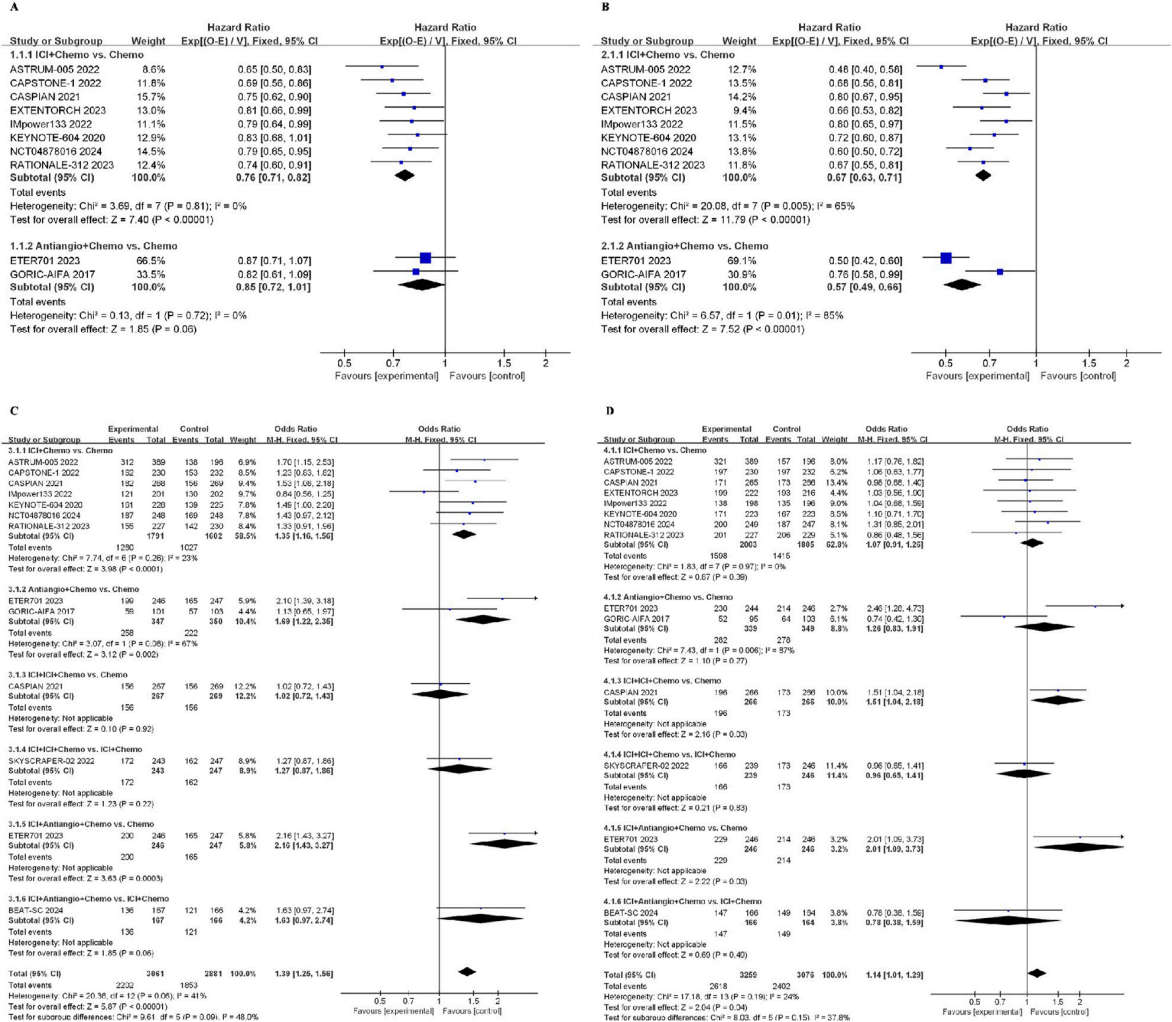
Forest plot of survival outcomes from integrated analysis of different therapy strategies (excluding ipilimumab + chemotherapy) in ES-SCLC patients: **(A)** OS, **(B)** PFS, **(C)** ORR, **(D)** AEs. OS, overall survival; PFS, progression-free survival; ORR, objective response rate; AE, adverse event; HR, hazard ratio; CI, confidence interval; ICI, immune checkpoint inhibitor; ICI + Chemo, ICI + chemotherapy; ICI + ICI + Chemo, ICI + ICI + chemotherapy; ICI + Antiangio + Chemo, ICI + antiangiogenic agent + chemotherapy; Antiangio + Chemo, antiangiogenic agent + chemotherapy; Chemo, chemotherapy.

Across all ICI + Chemo treatment strategies, the pooled hazard ratio (HR) for OS was 0.76 (95%CI: 0.71–0.82, I^2^ = 0%) compared with chemotherapy. Similarly, the pooled HR of Antiangio + Chemo was 0.85 (95%CI: 0.72–1.01, I^2^ = 0%) compared to chemotherapy (Figure 5A). As for PFS, the pooled HR of ICI + Chemo was 0.67 (95%CI: 0.63–0.71, I^2^ = 65%), and that of Antiangio + Chemo was 0.57 (95%CI: 0.49–0.66, I^2^ = 85%) compared to chemotherapy (Figure 5B). The odds ratio of the ORR was 1.35 (95%CI: 1.16–1.56, I^2^ = 23%) for ICI + Chemo, 1.69 (95%CI: 1.22–2.35, I^2^ = 67%) for Antiangio + Chemo, and 2.16 (95%CI: 1.43–3.27) for ICI + Antiangio + Chemo compared to chemotherapy (Figure 5C). ICI + ICI + Chemo (OR = 1.51, 95%CI: 1.04–2.18) and ICI + Antiangio + Chemo (OR = 2.01, 95%CI: 1.09–3.73) regimens showed more ≥ grade 3 adverse events than chemotherapy (Figure 5D).

**FIGURE 6:**
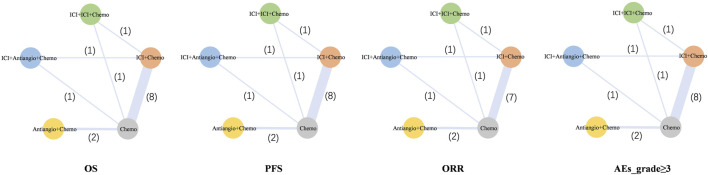
Network meta-analysis of comparisons of each outcome in various treatment strategies (excluding ipilimumab + chemotherapy) of ES-SCLC patients. Each circle represents a treatment strategy, and the line between the two points represents a comparison between the two treatment strategies. The numbers represent the count of involved studies, with the thickness of the lines proportional to the number of studies included. ICI, immune checkpoint inhibitor; ICI + Chemo, ICI + chemotherapy; ICI + ICI + Chemo, ICI + ICI + chemotherapy; ICI + Antiangio + Chemo, ICI + antiangiogenic agent + chemotherapy; Antiangio + Chemo, antiangiogenic agent + chemotherapy; Chemo, chemotherapy.

**FIGURE 7:**
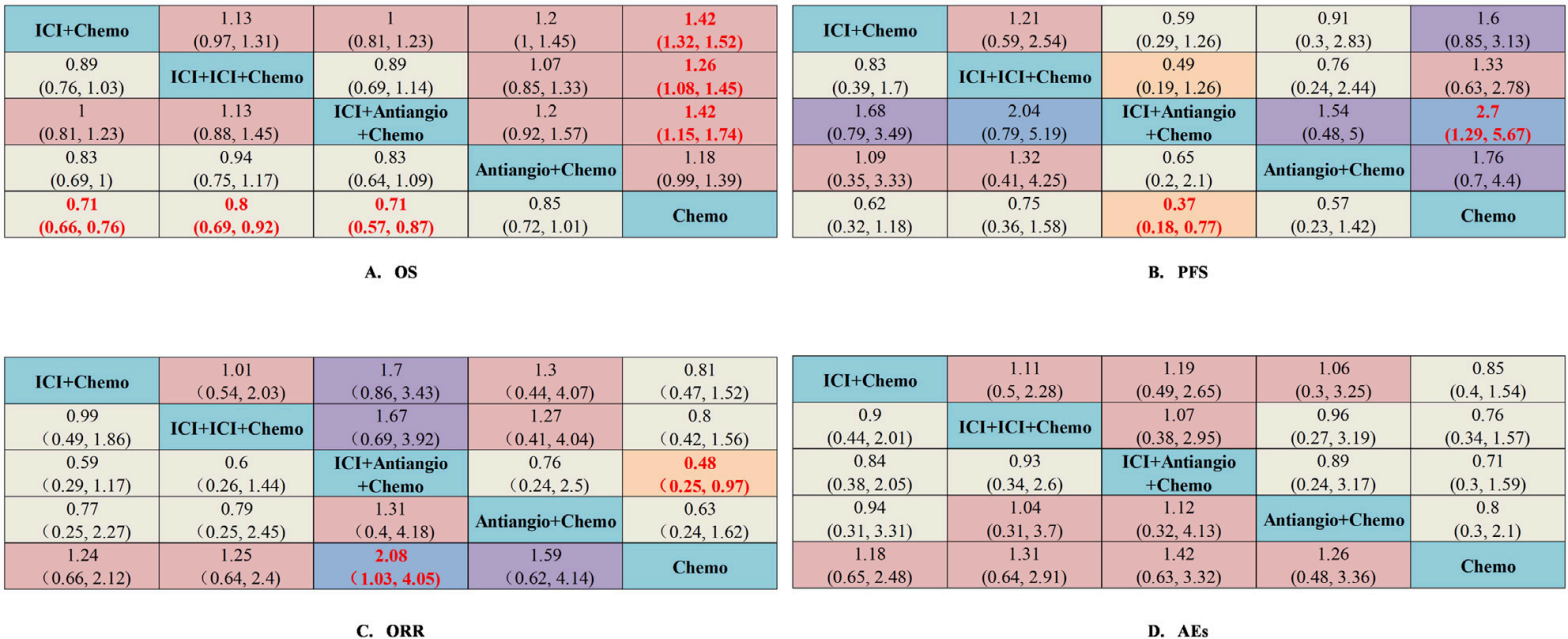
Efficacy and safety summaries from Bayesian network meta-analysis of different treatment strategies (excluding ipilimumab + chemotherapy) in ES-SCLC patients. **(A)** OS, **(B)** PFS, **(C)** ORR, **(D)** AEs. The data in each cell represent the HR or OR values that compare treatment effects as defined by the columns and rows. The cells are color-coded according to the HR or OR values: those with HRs or ORs below 0.5 are highlighted in orange, those ranging from 0.5 to 1 in tan, from 1 to 1.5 in light red, from 1.5 to 2 in purple, and those exceeding 2 in blue. The important results are highlighted in red and bold. ICI, immune checkpoint inhibitor; ICI + Chemo, ICI + chemotherapy; ICI + ICI + Chemo, ICI + ICI + chemotherapy; ICI + Antiangio + Chemo, ICI + antiangiogenic agent + chemotherapy; Antiangio + Chemo, antiangiogenic agent + chemotherapy; Chemo, chemotherapy.

The network was designed to allow for various comparisons of different treatment strategies (Figure 6). From the NMA results in Figure 7A, all treatment strategies, except for Antiangio + Chemo (HR = 0.85, 95%CI: 0.72–1.01), including ICI + Chemo (HR = 0.71, 95%CI: 0.66–0.76), ICI + ICI + Chemo (HR = 0.80, 95%CI: 0.69–0.92), and ICI + Antiangio + Chemo (HR = 0.71, 95%CI: 0.57–0.87) presented significantly better OS than chemotherapy. As for the NMA results for PFS (Figure 7B), only ICI + Antiangio + Chemo (HR = 0.37, 95%CI: 0.18–0.77) demonstrated a statistically significant improvement compared to chemotherapy alone. Other treatment strategies, including ICI + Chemo, ICI + ICI + Chemo, and Antiangio + Chemo, demonstrated better PFS without statistically significant differences. ICI + Antiangio + Chemo (OR = 2.08, 95%CI: 1.03–4.05) significantly increased the ORR compared to chemotherapy alone (Figure 7C). In terms of safety, no significant difference was observed in ≥grade 3 adverse events across the comparable treatment strategies in the network meta-analysis (Figure 7D). However, a higher likelihood of adverse events was observed with the ICI + Antiangio + Chemo regimen (OR = 1.42, 95%CI: 0.63–3.32).

### 3.5 Rank probability for treatment strategies

**FIGURE 8:**
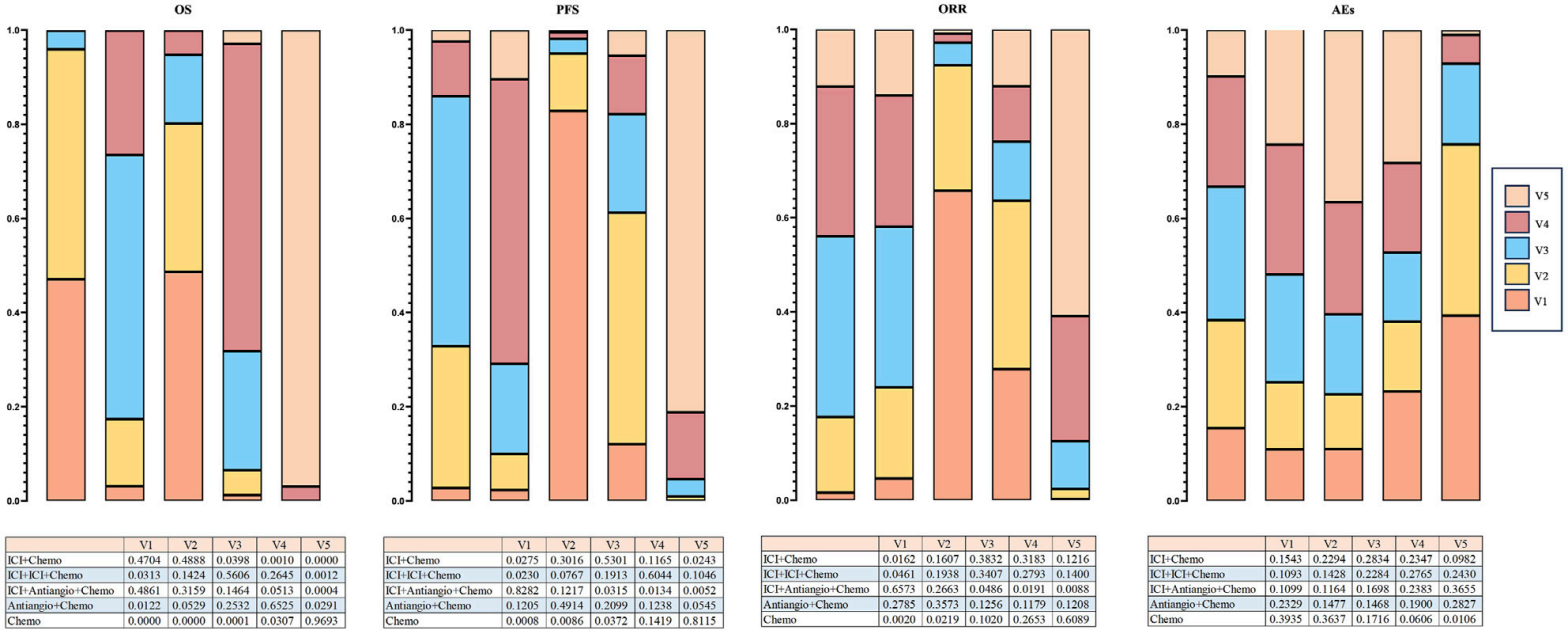
Bayesian ranking profiles indicate the effectiveness of treatment strategies or the likelihood of causing fewer grade ≥3 adverse events (AEs) (excluding ipilimumab + chemotherapy), ranked from most likely to least likely in the overall population. The top illustrates the possibility of improving overall survival (OS), extending progression-free survival (PFS), or causing fewer grade ≥3 adverse events (AEs). Under the premise of the same level, different image areas represent different possibilities of causing the outcome, and the corresponding values are presented in the table. ICI, immune checkpoint inhibitor; ICI + Chemo, ICI + chemotherapy; ICI + ICI + Chemo, ICI + ICI + chemotherapy; ICI + Antiangio + Chemo, ICI + antiangiogenic agent + chemotherapy; Antiangio + Chemo, antiangiogenic agent + chemotherapy; Chemo, chemotherapy.

Bayesian ranking profiles determined the probability that each regimen had the best outcome and safety profiles (Supplementary Figure S5). Of all the regimens, Ben + Anl + chemo had the highest probability (46.10%; 96.92%; 42.86%) of ranking first for OS, PFS, and ORR. In addition, Ben + Anl + chemo demonstrated the highest probability of ranking first for improving OS in female (76.63%), older (43.01%), without brain metastasis (46.18%), and without liver metastasis (71.52%) subgroups. For patients aged <65 years, Ser + chemo (37.05%) presented the highest possibility of ranking first for improving OS. For male patients, Ser + chemo and Ben + Anl + chemo demonstrated a comparable possibility of being ranked highest for OS improvement. As for patients with brain or liver metastasis, Anl + chemo (39.13%; with brain metastasis) and Tis + chemo (45.01%; with liver metastasis) showed the highest possibility of ranking first for improving OS, respectively. Finally, Bev + chemo (33.45%) had the probability of ranking first to cause fewer AEs of grade ≥3. Additionally, it could be seen from the Bayesian ranking profiles that ICI + Antiangio + Chemo demonstrated the highest probability (48.61%; 82.82%; 65.73%) in improving OS, PFS, and ORR in the network meta-analysis. ICI + Chemo ranked a close second in enhancing OS, achieving a rate of 47.04%, which was marginally lower than the ICI + Antiangio + Chemo group. Regarding safety profiles, Chemo showed a significantly lower incidence of grade ≥3 adverse events at 39.35% (Figure 8).

## 4 Discussion

In this meta-analysis, we comprehensively summarized and analyzed the efficacy and safety of currently available ICI or antiangiogenic agent-based regimens with chemotherapy for ES-SCLC and probed into the most appropriate therapy for patients. We also classified patients into five groups according to different treatment strategies, including chemotherapy monotherapy and its combination with ICI, antiangiogenic agents, ICI + antiangiogenic agents, and ICI + ICI for the first time. To the best of our knowledge, we have analyzed all available results of ICIs (PD-1, PD-L1, and CTLA-4 inhibitors) in combination with platinum and etoposide from phase III RCTs. We especially included the results of two novel ICI-based combinations, Soc + chemo and bevacizumab + Ate + chemo, for the first time, which were not included in previous ES-SCLC NMAs.

The results of the network meta-analysis were highly consistent, indicating that the efficacy can be improved by both anti-PD-1/PD-L1 antibodies plus chemotherapy versus chemotherapy alone. The combination of chemo with ICI boosts the immune system, which results in immunogenic tumor cell death and the release of immunogenic tumor-specific antigens, therefore activating the cytotoxic T-cell anti-tumor response. Specifically, the addition of antiangiogenic therapy to chemotherapy + ICI showed encouraging anti-tumor activity, offering the best benefits in terms of progression-free survival, overall survival, and objective response rate among all comparable treatment options. The network meta-analysis also showed that the antiangiogenic agent containing the Ben + Anl + chemo combination achieved significantly better PFS and OS with the lowest HR of 0.32 and 0.61 versus chemo and was also associated with the best ORR of 2.17 versus chemo.

Antiangiogenic agents, radiation therapy, and T-cell modulation are all under investigation for combination with immunotherapy. Studies have revealed the synergistic effects of antiangiogenic agents with immunotherapy could reprogram the tumor microenvironment from an immunosuppressive one to an immune permissive microenvironment and could be an opportunity to overcome immunotherapy resistance (Herbst et al., 2019; Taylor et al., 2020; Su et al., 2022). This concept has been explored in patients with advanced non-small-cell lung cancer (Socinski et al., 2018; Lu et al., 2023; Özgüroğlu et al., 2023). IMpower150 demonstrated that the combination of ICI plus anti-VEGF antibody and chemotherapy was associated with greater OS than with anti-VEGF antibody and chemotherapy (19.2 versus 14.7 months, respectively) (Socinski et al., 2018). Diverse combination regimens involving ICIs (PD-1, PD-L1, and CTLA-4 inhibitors) with inhibitors of vascular endothelial growth factor (VEGF) pathway such as anti-VEGF antibody, anti-VEGFR antibody, or VEGFR tyrosine kinase inhibitor (TKI) had shown improved clinical benefit compared to ICIs or antiangiogenic monotherapy, providing a hopeful solution to improve SCLC outcomes (Song et al., 2020; Socinski et al., 2021; Cheng A. et al., 2022).

In our study, the superior efficacy of benmelstobart + anlotinib + chemotherapy further supported the underlying synergistic action of anti-PD-L1 antibody and antiangiogenic agent with chemotherapy combination, in which the reversal of VEGF-mediated immunosuppression by anlotinib and chemotherapy-induced cell death potentiated T-cell-mediated killing activated by benmelstobart in the tumor microenvironment (Lin et al., 2018; Fukumura et al., 2018; Galon and Bruni, 2019). However, another combination treatment regimen showed the addition of VEGF antibody bevacizumab to first-line atezolizumab-platinum-etoposide only improved PFS, while the OS did not show improvement. These results might indicate that monoclonal antibodies and small molecule TKIs may not be exactly the same in the treatment against SCLC.

In terms of mechanism of action, bevacizumab inhibits angiogenesis by binding to VEGF-A and blocking its interaction with receptors. Meanwhile, recombinant enzyme assays *in vitro* indicated that anlotinib selectively inhibited VEGFR (1, 2, and 3), PDGFR (α and β), and FGFR (1, 2, 3, and 4) (Lin et al., 2018). Diverse results and mechanisms of different antiangiogenic drugs in combination with immunotherapy need to be further studied. In addition, the OS of chemotherapy combined with antiangiogenesis therapy is not better than a combination of immunotherapy or immunotherapy plus antiangiogenesis therapy. Our findings suggest that chemotherapy plus immunotherapy represents the backbone of therapeutic management for ES-SCLC.

In the group meta-analysis, ICI + Chemo, ICI + ICI + Chemo, and ICI + Antiangio + Chemo presented significantly better OS than chemotherapy. The benefits observed with these combination therapies over their individual components suggested a synergistic effect of ICI-based and ICI plus antiangiogenic agent**-**based therapies in enhancing the anticancer activity in ES-SCLC. From the group NMA results for PFS and ORR, only ICI + Antiangio + Chemo (HR = 0.37, 95%CI: 0.18–0.77; OR = 2.08, 95%CI: 1.03–4.05) demonstrated a statistically significantly improvement compared to chemotherapy alone, suggesting that chemotherapy-induced neoantigen release and antiangiogenesis-induced immune reprogramming play important roles in activating the tumor microenvironment from an immunosuppressive state (Zitvogel et al., 2013; Fukumura et al., 2018; Galon and Bruni, 2019).

In the present study, only Tis + chemo significantly improved the OS of patients with liver metastasis (LM). It has been known that antiangiogenic agents could reverse the VEGF-mediated immunosuppression as an underlying choice to enhance the anti-tumor activity of ICIs in patients with LM. Therefore, combination treatment with an ICI and an antiangiogenic agent was an effective strategy for the treatment of primary hepatocellular carcinoma as well as many solid tumors with LM (Ren et al., 2021; Sangro et al., 2021; Cheng A. et al., 2022). However, benmelstobart + anlotinib + chemotherapy failed to significantly prolong the OS of patients with LMs (HR = 0.79). These findings may lie in the immunosuppressive microenvironment within LMs, which undermined the efficacy of immunotherapy (Horst et al., 2016). The results may also be influenced by the smaller sample size of the LM subgroup and thus need further exploration. Similarly, the current study also showed no survival benefit from ICIs + chemo versus chemo in patients with brain metastases (BMs), even with anlotinib in combination, which may be due to the poor prognosis of brain metastases and the small sample size of enrolled patients with BMs.

In terms of safety and toxicity, the ICI + chemo combinations were not associated with unexpected safety events, and all adverse events were generally manageable, as previously reported. Patients treated with Ben + Anl + chemo, Dur + Tre + chemo, and Anl + chemo experienced a higher likelihood of grade ≥3 AEs. In the group meta-analysis, no significant difference was observed across the comparable treatment strategies. A higher likelihood of adverse events was observed with the ICI + Antiangio + Chemo regimen. The additional AEs of Dur + Tre + chemo may be induced by two immune checkpoint inhibitors. AEs of Ben + Anl + chemo and Anl + chemo might be mainly induced by the addition of antiangiogenic agents, including hypertension, proteinuria, and bleeding, which were generally manageable and tolerable. It should be noted that among all the regimens containing antiangiogenic agents, the regimens containing anlotinib have a higher incidence of adverse reactions than those containing bevacizumab, especially hematological toxicity and hypertension. This might be associated with anlotinib’s multi-target effects interfering with normal tissue functions. In general, the adverse effects were predictable, and most adverse events were manageable.

Immunotherapy or antiangiogenic agents combined with chemotherapy are important combined anti-tumor therapy strategies. By comparing the efficacy and safety profiles of novel treatment combinations for ES-SCLC, this timely study aims to provide instruction in selecting the most appropriate immunotherapy agent and combination pattern for ES-SCLC patients in clinical work. The novel combination of ICI and antiangiogenic agent with chemotherapy yielded the best survival benefit for ES-SCLC patients, although it caused more adverse effects, which were generally well manageable.

The current study has some innate limitations. First, there might be publication bias and potential selection bias limitations because of the missing unpublished literature, though we have proposed a comprehensive retrieval strategy. Second, the comparisons between different ICI-based combinations were not head-to-head and relied on the transitivity and consistency assumptions of different clinical trials. Third, the grouped network meta-analysis examined a relatively low number of trials and participants involving immunotherapy + antiangiogenic agents + chemotherapy. Fourth, some data were extracted from slide pictures presented in meetings, which might be different from the real trial data. Finally, the diversity in patient races among trials should be considered.

## 5 Conclusion

For individuals with ES-SCLC, ICI + Antiangio + Chemo was identified as the optimal treatment option because of better OS, PFS, and ORR. Benmelstobart + anlotinib + chemotherapy demonstrated the best survival benefit compared to chemotherapy. The toxicity of ICI + Antiangio + Chemo was acceptable but needed careful attention. These findings clarified the roles of ICI and antiangiogenic agent-based treatment strategies in this population.

## Data Availability

The original contributions presented in the study are included in the article/Supplementary Material; further inquiries can be directed to the corresponding authors.
