# Supplementary material for: Efficacy and safety of immunotherapy or antiangiogenic agent-based treatment strategies versus chemotherapy as first-line treatment for extensive-stage small cell lung cancer: a network meta-analysis
Source: Front Pharmacol. 2025 Jun 9;16:1539246. doi: 10.3389/fphar.2025.1539246 (PMC12183167; doi:10.3389/fphar.2025.1539246)
Supplement: Supplementary file 1 [file DataSheet1.docx]

**Efficacy and safety of immunotherapy or antiangiogenic agents-based treatment strategies versus chemotherapy as first--line treatment for extensive-stage small cell lung cancer: A network meta-analysis**

Supplementary Materials

| Table of Contents | | |
| --- | --- | --- |
| Title | Content | Page |
| Table S1 | Checklist of the PRISMA extension for network meta-analysis. | 2-4 |
| Table S2 | Literature search strategy. | 5-6 |
| Table S3 | Baseline clinical and disease characteristics of trials included in the Network Meta-analysis. | 7-8 |
| Table S4 | Comparison of the OS rate of combined therapy with standard chemotherapy at the 3rd, 6th, 9th, 12th, 15th, 18th, 21st, and 24th month, ORs and 95%CI. | 9 |
| Table S5 | Matrix of pairwise comparisons of regimens on OS. | 10-22 |
| Table S6 | Comparison of the PFS rate of combined therapy to standard chemotherapy at t the 1st, 2nd, 3rd, 4th, 5th, 6th, 7th, 8th, 9th, 10th, 11th and 12th month, ORs and 95%CI. | 23 |
| Table S7 | Matrix of pairwise comparisons of regimens on PFS. | 24-36 |
| Table S8 | Incidence of Grade≥3 adverse events in each immunotherapy combination (%). | 37 |
| Figure S1 | Results of risk of bias assessment. | 38 |
| Figure S2 | The Brooks-Gelman-Rubin diagnostic and the density trace plot | 39-46 |
| Figure S3 | The rank-heat plot presented in this study illustrates the evaluation of several first-line treatment regimens for patients with extensive-stage small cell lung cancer (ES-SCLC). | 47 |
| Figure S4 | Efficacy and Safety summaries from Bayesian Network Meta-Analysis in SCLC patients. | 48-51 |
| Figure S5 | Bayesian ranking profiles indicate the likelihood of causing fewer grade ≥3 adverse events (AEs) or the effectiveness of immunotherapies, ranked from most likely to least likely in the overall population. | 52 |
| Figure S6 | The Brooks-Gelman-Rubin diagnostic and the density trace plot in group meta-analysis. | 53-56 |
| Figure S7 | Forest plot of survival outcomes from integrated analysis of different therapy strategies in SCLC patients. (A) OS; (B) PFS; (C) ORR; (D) AEs. | 57-60 |
| Figure S8 | Network meta-analysis of comparisons on each outcome in various treatment strategies of ES-SCLC patients. | 61 |
| Figure S9 | Efficacy and Safety summaries from Bayesian Network Meta-Analysis of different treatment strategies in SCLC patients. | 62-63 |
| Figure S10 | Bayesian ranking profiles indicate the likelihood of causing fewer grade ≥3 adverse events (AEs) or the effectiveness of treatment strategies, ranked from most likely to least likely in the overall population. | 64 |
| Figure S11 | The Brooks-Gelman-Rubin diagnostic and the density trace plot in group meta-analysis (Excluding ipilimumab + chemotherapy). | 65-68 |

| Table S1 Checklist of the PRISMA extension for network meta-analysis. | | | |
| --- | --- | --- | --- |
| Topic | No. | Item | Location where item is reported |
| TITLE |  |  |  |
| Title | 1 | Identify the report as a systematic review. |  |
| ABSTRACT |  |  |  |
| Abstract | 2 | See the PRISMA 2020 for Abstracts checklist |  |
| INTRODUCTION |  |  |  |
| Rationale | 3 | Describe the rationale for the review in the context of existing knowledge. |  |
| Objectives | 4 | Provide an explicit statement of the objective(s) or question(s) the review addresses. |  |
| METHODS |  |  |  |
| Eligibility criteria | 5 | Specify the inclusion and exclusion criteria for the review and how studies were grouped for the syntheses. |  |
| Information sources | 6 | Specify all databases, registers, websites, organisations, reference lists and other sources searched or consulted to identify studies. Specify the date when each source was last searched or consulted. |  |
| Search strategy | 7 | Present the full search strategies for all databases, registers and websites, including any filters and limits used. |  |
| Selection process | 8 | Specify the methods used to decide whether a study met the inclusion criteria of the review, including how many reviewers screened each record and each report retrieved, whether they worked independently, and if applicable, details of automation tools used in the process. |  |
| Data collection process | 9 | Specify the methods used to collect data from reports, including how many reviewers collected data from each report, whether they worked independently, any processes for obtaining or confirming data from study investigators, and if applicable, details of automation tools used in the process. |  |
| Data items | 10a | List and define all outcomes for which data were sought. Specify whether all results that were compatible with each outcome domain in each study were sought (e.g. for all measures, time points, analyses), and if not, the methods used to decide which results to collect. |  |
|  | 10b | List and define all other variables for which data were sought (e.g. participant and intervention characteristics, funding sources). Describe any assumptions made about any missing or unclear information. |  |
| Study risk of bias assessment | 11 | Specify the methods used to assess risk of bias in the included studies, including details of the tool(s) used, how many reviewers assessed each study and whether they worked independently, and if applicable, details of automation tools used in the process. |  |
| Effect measures | 12 | Specify for each outcome the effect measure(s) (e.g. risk ratio, mean difference) used in the synthesis or presentation of results. |  |
| Synthesis methods | 13a | Describe the processes used to decide which studies were eligible for each synthesis (e.g. tabulating the study intervention characteristics and comparing against the planned groups for each synthesis (item 5)). |  |
|  | 13b | Describe any methods required to prepare the data for presentation or synthesis, such as handling of missing summary statistics, or data conversions. |  |
|  | 13c | Describe any methods used to tabulate or visually display results of individual studies and syntheses. |  |
|  | 13d | Describe any methods used to synthesize results and provide a rationale for the choice(s). If meta-analysis was performed, describe the model(s), method(s) to identify the presence and extent of statistical heterogeneity, and software package(s) used. |  |
|  | 13e | Describe any methods used to explore possible causes of heterogeneity among study results (e.g. subgroup analysis, meta-regression). |  |
|  | 13f | Describe any sensitivity analyses conducted to assess robustness of the synthesized results. |  |
| Reporting bias assessment | 14 | Describe any methods used to assess risk of bias due to missing results in a synthesis (arising from reporting biases). |  |
| Certainty assessment | 15 | Describe any methods used to assess certainty (or confidence) in the body of evidence for an outcome. |  |
| RESULTS |  |  |  |
| Study selection | 16a | Describe the results of the search and selection process, from the number of records identified in the search to the number of studies included in the review, ideally using a flow diagram. |  |
|  | 16b | Cite studies that might appear to meet the inclusion criteria, but which were excluded, and explain why they were excluded. |  |
| Study characteristics | 17 | Cite each included study and present its characteristics. |  |
| Risk of bias in studies | 18 | Present assessments of risk of bias for each included study. |  |
| Results of individual studies | 19 | For all outcomes, present, for each study: (a) summary statistics for each group (where appropriate) and (b) an effect estimate and its precision (e.g. confidence/credible interval), ideally using structured tables or plots. |  |
| Results of syntheses | 20a | For each synthesis, briefly summarize the characteristics and risk of bias among contributing studies. |  |
|  | 20b | Present results of all statistical syntheses conducted. If meta-analysis was done, present for each the summary estimate and its precision (e.g. confidence/credible interval) and measures of statistical heterogeneity. If comparing groups, describe the direction of the effect. |  |
|  | 20c | Present results of all investigations of possible causes of heterogeneity among study results. |  |
|  | 20d | Present results of all sensitivity analyses conducted to assess the robustness of the synthesized results. |  |
| Reporting biases | 21 | Present assessments of risk of bias due to missing results (arising from reporting biases) for each synthesis assessed. |  |
| Certainty of evidence | 22 | Present assessments of certainty (or confidence) in the body of evidence for each outcome assessed. |  |
| DISCUSSION |  |  |  |
| Discussion | 23a | Provide a general interpretation of the results in the context of other evidence. |  |
|  | 23b | Discuss any limitations of the evidence included in the review. |  |
|  | 23c | Discuss any limitations of the review processes used. |  |
|  | 23d | Discuss implications of the results for practice, policy, and future research. |  |
| OTHER INFORMATION |  |  |  |
| Registration and protocol | 24a | Provide registration information for the review, including register name and registration number, or state that the review was not registered. |  |
|  | 24b | Indicate where the review protocol can be accessed, or state that a protocol was not prepared. |  |
|  | 24c | Describe and explain any amendments to information provided at registration or in the protocol. |  |
| Support | 25 | Describe sources of financial or non-financial support for the review, and the role of the funders or sponsors in the review. |  |
| Competing interests | 26 | Declare any competing interests of review authors. |  |
| Availability of data, code and other materials | 27 | Report which of the following are publicly available and where they can be found: template data collection forms; data extracted from included studies; data used for all analyses; analytic code; any other materials used in the review. |  |

| Table S2 Literature search strategy | |
| --- | --- |
| (((("Small Cell Lung Carcinoma"[Mesh]) OR (((((Small Cell Lung Cancer) OR (Oat Cell Lung Cancer)) OR (Small Cell Cancer Of The Lung)) OR (Carcinoma, Small Cell Lung)) OR (Oat Cell Carcinoma of Lung))) AND (((extensive-stage) OR (extensive-disease)))) AND ((((((((((((((((((immunotherapy) OR (immune checkpoint inhibitor)) OR (tremelimumab)) OR (nivolumab)) OR (ipilimumab)) OR (pembrolizumab)) OR (durvalumab)) OR (adebrelimab)) OR (atezolizumab)) OR (serplulimab)) OR (tiragolumab)) OR (benmelstobart)) OR (tislelizumab)) OR (toripalimab)) OR (socazolimab)) OR (bevacizumab)) OR (anlotinib)) OR ("Angiogenesis"[Mesh]))) AND (((randomized controlled trial) OR (randomized)) OR (placebo)) | |
| Search Strategy in PubMed | |
| #1 | Search: "Small Cell Lung Carcinoma"[Mesh] |
| #2 | Search: ((((Small Cell Lung Cancer) OR (Oat Cell Lung Cancer)) OR (Small Cell Cancer Of The Lung)) OR (Carcinoma, Small Cell Lung)) OR (Oat Cell Carcinoma of Lung) |
| #3 | #1 OR #2 |
| #4 | Search: (extensive-stage) OR (extensive-disease) |
| #5 | Search: (((((((((((((((((immunotherapy) OR (immune checkpoint inhibitor)) OR (tremelimumab)) OR (nivolumab)) OR (ipilimumab)) OR (pembrolizumab)) OR (durvalumab)) OR (adebrelimab)) OR (atezolizumab)) OR (serplulimab)) OR (tiragolumab)) OR (benmelstobart)) OR (tislelizumab)) OR (toripalimab)) OR (socazolimab)) OR (bevacizumab)) OR (anlotinib)) OR ("Angiogenesis"[Mesh]) |
| #6 | Search: ((randomized controlled trial) OR (randomized)) OR (placebo) |
| #7 | #3 AND #4 AND #5 AND #6 |

| Search Strategy in Embase | |
| --- | --- |
| #1 | Search: 'small cell lung carcinoma':ti,ab,kw OR 'small cell lung cancer':ti,ab,kw OR 'oat cell lung cancer':ti,ab,kw OR 'small cell cancer of the lung':ti,ab,kw OR 'carcinoma, small cell lung':ti,ab,kw OR 'oat cell carcinoma of lung':ti,ab,kw |
| #2 | Search: 'extensive stage':ti,ab,kw OR 'extensive disease':ti,ab,kw |
| #3 | Search: 'immunotherapy':ti,ab,kw OR 'immune checkpoint inhibitor':ti,ab,kw OR tremelimumab:ti,ab,kw OR nivolumab:ti,ab,kw OR ipilimumab:ti,ab,kw OR pembrolizumab:ti,ab,kw OR durvalumab:ti,ab,kw OR adebrelimab:ti,ab,kw OR atezolizumab:ti,ab,kw OR serplulimab:ti,ab,kw OR tiragolumab:ti,ab,kw OR benmelstobart:ti,ab,kw OR tislelizumab:ti,ab,kw OR toripalimab:ti,ab,kw OR socazolimab:ti,ab,kw OR bevacizumab:ti,ab,kw OR anlotinib:ti,ab,kw OR 'angiogenesis':ti,ab,kw |
| #4 | Search: 'randomized controlled trial':ti,ab,kw OR randomized:ti,ab,kw OR placebo:ti,ab,kw |
| #5 | #1 AND #2 AND #3 AND #4 |

| Search Strategy in Web of Science | |
| --- | --- |
| #1 | Search: (((((TS=(Small cell lung carcinoma)) OR TS=(Small cell lung cancer)) OR TS=(Oat Cell Lung Cancer)) OR TS=(Small Cell Cancer Of The Lung)) OR TS=(Carcinoma, Small Cell Lung)) OR TS=(Oat Cell Carcinoma of Lung) |
| #2 | Search: (TS=(extensive-stage)) OR TS=(extensive-disease) |
| #3 | Search: (((((((((((((((((TS=(immunotherapy)) OR TS=(immune checkpoint inhibitor)) OR TS=(tremelimumab)) OR TS=(nivolumab)) OR TS=(ipilimumab)) OR TS=(pembrolizumab)) OR TS=(durvalumab)) OR TS=(adebrelimab)) OR TS=(atezolizumab)) OR TS=(serplulimab)) OR TS=(tiragolumab)) OR TS=(benmelstobart)) OR TS=(tislelizumab)) OR TS=(toripalimab)) OR TS=(socazolimab)) OR TS=(bevacizumab)) OR TS=(anlotinib)) OR TS=(Angiogenesis) |
| #4 | Search: ((TS=(randomized controlled trial)) OR TS=(randomized)) OR TS=(placebo) |
| #5 | #1 AND #2 AND #3 AND #4 |

| Search Strategy in ClinicalTrials.gov |
| --- |
| ((Small cell lung carcinoma) OR (Small cell lung cancer) OR (Oat Cell Lung Cancer) OR (Small Cell Cancer Of The Lung) OR (Carcinoma, Small Cell Lung) OR (Oat Cell Carcinoma of Lung)) AND ((extensive-stage) OR (extensive-disease)) AND ((immunotherapy) OR (immune checkpoint inhibitor) OR (tremelimumab) OR (nivolumab) OR (ipilimumab) OR (pembrolizumab) OR (durvalumab) OR (adebrelimab) OR (atezolizumab) OR (serplulimab) OR (tiragolumab) OR (benmelstobart) OR (tislelizumab) OR (toripalimab) OR (socazolimab) OR (bevacizumab) OR (anlotinib) OR (antiangiogenic)) AND ((randomized controlled trial) OR (randomized) OR (Placebo)) |

| Table S3 Baseline Clinical and disease Characteristics of Trials Included in the Network Meta-analysis. | | | | | | | | | | | | | | | | | |
| --- | --- | --- | --- | --- | --- | --- | --- | --- | --- | --- | --- | --- | --- | --- | --- | --- | --- |
|  | | CAPSTONE-1(n=462) | | IMpower133(n=403) | | CASPIAN(n=805) | | | Reck.et al.(n=954) | | KEYNOTE-604(n=446) | | ASTRUM-005(n=585) | | ETER701(n=493) | | |
|  | | Ade Group  (n=230) | Pla Group  (n=232) | Ate Group  (n=201) | Pla Group  (n=202) | Dur + Tre Group  (n=268) | Dur Group  (n=268) | Pla Group  (n=269) | Ipi Group  (n = 478) | Pla Group  (n = 476) | Pem Group  (n=223) | Pla Group  (n=223) | Ser Group  (n = 389) | Pla Group  (n = 196) | Ben + Anl Group  (n=246) | Anl Group  (n=247) | Pla Group  (n=247) |
| Age | | | | | | | | | | | | | | | | | |
|  | <65 | 67% | 63% | 55% | 53% | 57% | 62% | 58% | 63% | 58% | 50% | 45% | 60% | 61% | NR | NR | NR |
|  | ≥65 | 33% | 37% | 45% | 48% | 43% | 38% | 42% | 37% | 42% | 50% | 55% | 40% | 39% | NR | NR | NR |
| Gender | | | | | | | | | | | | | | | | | |
|  | Male | 80% | 81% | 64% | 65% | 75% | 71% | 68% | 66% | 68% | 67% | 63% | 82% | 84% | 85% | 84% | 84% |
|  | Female | 20% | 19% | 36% | 35% | 25% | 29% | 32% | 34% | 32% | 33% | 37% | 19% | 16% | 15% | 16% | 16% |
| Smoking status | | | | | | | | | | | | | | | | | |
|  | Current or former | 78% | 13% | 96% | 99% | 94% | 92% | 94% | 56% | 57% | 97% | 96% | 79% | 82% | 76% | 78% | 78% |
|  | Never or unknow | 22% | 87% | 5% | 2% | 6% | 8% | 6% | 44% | 43% | 4% | 4% | 21% | 18% | 24% | 22% | 22% |
| ECOG performance status | | | | | | | | | | | | | | | | | |
|  | 0 | 14% | 13% | 36% | 33% | 41% | 37% | 33% | 29% | 31% | 26% | 25% | 18% | 16% | 19% | 19% | 19% |
|  | ≥1 | 86% | 87% | 64% | 67% | 59% | 63% | 67% | 71% | 69% | 74% | 75% | 82% | 84% | 81% | 81% | 81% |
| Brain metastases at enrollment | | | | | | | | | | | | | | | | | |
|  | Yes | 2% | 2% | 8.5% | 8.9% | 14% | 10% | 10% | 12% | 10% | 15% | 10% | 13% | 14% | 10% | 11% | 11% |
|  | No | 98% | 98% | 91.5% | 91.1% | 86% | 90% | 90% | 88% | 90% | 86% | 90% | 87% | 86% | 90% | 89% | 89% |
| Liver metastases at enrollment | | | | | | | | | | | | | | | | | |
|  | Yes | 32% | 32% | 38.3% | 35.6% | 44% | 40% | 39% | NR | NR | 42% | 41% | 25% | 26% | 32% | 32% | 32% |
|  | No | 68% | 68% | 61.7% | 64.4% | 56% | 60% | 61% | NR | NR | 58% | 59% | 75% | 74% | 68% | 68% | 68% |
|  |  |  |  |  |  |  |  |  |  |  |  |  |  |  |  |  |  |
|  |  | SKYSCRAPER-02(n=490) | | EXTENTORCH(n=442) | | RATIONALE-312(n=457) | | BEAT-SC(n=333) | | NCT04878016(n=496) | | GORIC-AIFA(n=204) | |  |  |  |  |
|  |  | Tir + Ate Group  (n=243) | Ate Group  (n=247) | Tor Group  (n=223) | Pla Group  (n=219) | Tis group  (n=227) | Pla Group  (n=230) | Bev + Ate Group  （n=167） | Pla Group (n=166) | Soc Group (n=248) | Pla Group (n=248) | Bev Group (n=103) | Pla Group （n=101） |  |  |  |  |
| Age | | | | | | | | | | | | | | | | | |
|  | <65 | 48% | 47% | 65% | 57% | 61% | 65% | NR | NR | 52% | 60.5% | NR | NR |  |  |  |  |
|  | ≥65 | 52% | 53% | 35% | 43% | 39% | 35% | NR | NR | 48% | 39.5% | NR | NR |  |  |  |  |
| Gender | | | | | | | | | | | | | | | | | |
|  | Male | 67% | 66% | 82% | 84% | 82% | 81% | 82.6% | 82.5 | 83.1% | 82.7% | 68.0% | 68.3% |  |  |  |  |
|  | Female | 33% | 34% | 18% | 16% | 18% | 19% | 17.4% | 17.5% | 16.9% | 17.3% | 32.0% | 31.7% |  |  |  |  |
| Smoking status | | | | | | | | | | | | | | | | | |
|  | Current or former | 96% | 96% | 78.5% | 78% | 77% | 74% | 87.3% | 87.9% | NR | NR | NR | NR |  |  |  |  |
|  | Never or unknow | 4% | 4% | 21.5% | 22% | 23% | 26% | 12.7% | 12.1% | NR | NR | NR | NR |  |  |  |  |
| ECOG performance status | | | | | | | | | | | | | | | | | |
|  | 0 | 35% | 33% | 19% | 17% | 15% | 15% | 28.7% | 28.3% | 17.7% | 17.3% | 55.3% | 52.5% |  |  |  |  |
|  | ≥1 | 65% | 67% | 81% | 83% | 85% | 85% | 71.3% | 71.7% | 82.3% | 82.7% | 44.7% | 47.5% |  |  |  |  |
| Brain metastases at enrollment | | | | | | | | | | | | | | | | | |
|  | Yes | 19% | 19% | 1% | 2% | ＜1% | 2% | 22.8% | 16.3% | 4.0% | 2.8% | NR | NR |  |  |  |  |
|  | No | 81% | 81% | 99% | 98% | 99% | 98% | 77.2% | 83.7% | 96% | 97.2% | NR | NR |  |  |  |  |
| Liver metastases at enrollment | | | | | | | | | | | | | | | | | |
|  | Yes | 37% | 38% | 27% | 23% | 28% | 26% | NR | NR | NR | NR | NR | NR |  |  |  |  |
|  | No | 63% | 62% | 73% | 77% | 72% | 74% | NR | NR | NR | NR | NR | NR |  |  |  |  |
| Ipi, ipilimumab; Pem, pembrolizumab; Dur + Tre, durvalumab + tremelimuamb; Dur, durvalumab; Ade, adebrelimab; Ate, atezolizumab; Ser, serplulimab; Tir + Ate, tiragolumab + atezolizumab; Ben + Anl, benmelstobart + anlotinib; Anl, anlotinib; Tis, tislelizumab; Tor, toripalimab; Bev + Ate, bevacizumab + atezolizumab; Bev, bevacizumab; Soc, socazolimab; ECOG, Eastern Cooperative Oncology Group. | | | | | | | | | | | | | | | | | |

| Table S4 Comparison of the OS rate of combined therapy with standard chemotherapy at the 3rd, 6th, 9th, 12th, 15th, 18th, 21st, and 24th month, ORs and 95%CI. | | | | | | | | | | | | | |
| --- | --- | --- | --- | --- | --- | --- | --- | --- | --- | --- | --- | --- | --- |
| Time (months) | Ade + chemo | Ate + chemo | Dur + Tre  + chemo | Dur + chemo | Ipi + chemo | Pem + chemo | Ser + chemo | Ben + Anl + chemo | Tis + chemo | Tor + chemo | Anl + chemo | Soc + chemo | Bev + chemo |
| 3rd | 0.51 (0.17, 1.4) | 1.48 (0.62, 3.7) | 0.72  (0.39, 1.3) | 0.99 (0.53, 1.85) | 0.82  (0.34, 1.93) | 0.49 (0.24, 0.98) | 1.37 (0.55, 3.29) | 1.27 (0.32, 5.36) | 0.5 (0.19, 1.18) | 0.82 (0.23, 2.79) | 0.97  (0.37, 2.56) | 1.41 (0.55, 3.78) | 1.14 (0.52, 2.54) |
| 6th | 0.75  (0.41, 1.36) | 1.16 (0.67, 2) | 0.8 (0.53, 1.2) | 0.99  (0.65, 1.53) | 1.22 (0.87, 1.73) | 1.09 (0.71, 1.69) | 2.29  (1.4, 3.75) | 1.57 (0.89, 2.83) | 0.99  (0.55, 1.75) | 1.47  (0.81, 2.7) | 1.34 (0.78, 2.32) | 1.38 (0.82, 2.36) | 1.25  (0.67, 2.35) |
| 9th | 1.08 (0.71, 1.63) | 1.22 (0.82, 1.84) | 1.06 (0.75, 1.49) | 1.46  (1.03, 2.07) | 1.11 (0.86, 1.44) | 1.06 (0.73, 1.54) | 1.71  (1.16, 2.51) | 1.31 (0.88, 1.97) | 1.1  (0.73, 1.67) | 1.35 (0.87, 2.11) | 1.18 (0.8, 1.73) | 1.19  (0.81, 1.76) | 1.22 (0.7, 2.12) |
| 12th | 1.49 (1.03, 2.17) | 1.67 (1.12, 2.48) | 1.19 (0.85, 1.68) | 1.74  (1.23, 2.45) | 1.07  (0.83, 1.38) | 1.28 (0.88, 1.87) | 1.6 (1.12, 2.28) | 1.74 (1.21, 2.5) | 1.19 (0.82, 1.75) | 1.42  (0.97, 2.08) | 1.18  (0.83, 1.68) | 1.38 (0.97, 1.98) | 1.58  (0.86, 2.92) |
| 15th | 1.61  (1.11, 2.33) | 1.96 (1.3, 2.96) | 1.21 (0.85, 1.74) | 1.56 (1.1, 2.23) | 1.22 (0.92, 1.61) | 1.59  (1.07, 2.38) | 1.92 (1.36, 2.71) | 2.28 (1.58, 3.29) | 1.46 (1.01, 2.11) | 1.43  (0.98, 2.08) | 1.33 (0.93, 1.91) | 1.73  (1.2, 2.5) | 1.83  (0.94, 3.65) |
| 18th | 1.88 (1.27, 2.77) | 1.92 (1.24, 3.02) | 1.38 (0.95, 2.03) | 1.48 (1.02, 2.15) | 1.24  (0.89, 1.73) | 1.49 (0.98, 2.29) | 1.62 (1.14, 2.29) | 3.1  (2.14, 4.55) | 1.45 (1, 2.14) | 1.37 (0.93, 2.03) | 1.49 (1.01, 2.19) | 1.43  (0.97, 2.11) | 1.62 (0.75, 3.6) |
| 21st | 1.97  (1.31, 2.97) | 1.51 (0.94, 2.44) | 1.47  (0.98, 2.21) | 1.44  (0.96, 2.16) | 1.08  (0.73, 1.58) | 1.82 (1.14, 2.92) | - | 2.49 (1.7, 3.68) | 1.56 (1.05, 2.33) | 1.44 (0.95, 2.2) | 1.2 (0.8, 1.79) | 1.15  (0.75, 1.77) | 1.59 (0.7, 3.72) |
| 24th | 2.25 (1.44, 3.54) | 1.34 (0.82, 2.22) | 1.73  (1.12, 2.7) | 1.71  (1.09, 2.68) | 1.46  (0.95, 2.26) | 2.31 (1.38, 3.97) | - | 2.25 (1.53, 3.34) | 1.78 (1.17, 2.73) | 1.41  (0.9, 2.22) | 0.9 (0.6, 1.37) | 4.19 (2.33, 7.98) | 1.78  (0.72, 4.73) |
| OS, overall survival; OR, Odds ratio; HR, Hazard ratio; CI, confidence interval; Ade + chemo, adebrelimab + chemotherapy; Ate + chemo, atezolizumab + chemotherapy; Dur + Tre + chemo, durvalumab + tremelimuamb + chemotherapy; Dur + chemo, durvalumab + chemotherapy; Ipi + chemo, ipilimumab + chemotherapy; Pem + chemo, pembrolizumab + chemotherapy; Ser + chemo, serplulimab + chemotherapy; Ben + Anl + chemo, benmelstobart + anlotinib + chemotherapy; Tis + chemo, tislelizumab + chemotherapy; Tor + chemo, toripalimab + chemotherapy; Anl + chemo, anlotinib + chemotherapy; Soc + chemo, socazolimab + chemotherapy; Bev + chemo, bevacizumab + chemotherapy. | | | | | | | | | | | | | |

Table S5 Matrix of pairwise comparisons of regimens on OS.

Matrix of pairwise comparisons of Ade + chemo (shown as odds ratios and 95% confidence intervals).

| 3rd | 1.46 (0.46, 5.06) | 2.11 (0.72, 6.76) | 2.91 (1, 9.22) | 3.12 (1.07, 9.88) | 3.65 (1.25, 11.61) | 3.83 (1.3, 12.46) | 4.39 (1.46, 14.36) | 1.94 (0.72, 5.83) |
| --- | --- | --- | --- | --- | --- | --- | --- | --- |
| 0.68 (0.2, 2.19) | 6th | 1.44 (0.7, 2.99) | 1.99 (0.98, 4.04) | 2.14 (1.07, 4.33) | 2.5 (1.23, 5.1) | 2.63 (1.28, 5.41) | 3 (1.43, 6.35) | 1.33 (0.74, 2.43) |
| 0.47 (0.15, 1.39) | 0.69 (0.33, 1.43) | 9th | 1.38 (0.79, 2.41) | 1.49 (0.85, 2.58) | 1.73 (0.99, 3.07) | 1.82 (1.02, 3.28) | 2.08 (1.13, 3.85) | 0.92 (0.61, 1.4) |
| 0.34 (0.11, 1) | 0.5 (0.25, 1.02) | 0.72 (0.41, 1.26) | 12th | 1.08 (0.64, 1.82) | 1.25 (0.73, 2.16) | 1.32 (0.76, 2.3) | 1.51 (0.84, 2.7) | 0.67 (0.46, 0.97) |
| 0.32 (0.1, 0.93) | 0.47 (0.23, 0.94) | 0.67 (0.39, 1.17) | 0.93 (0.55, 1.57) | 15th | 1.17 (0.69, 2) | 1.23 (0.71, 2.13) | 1.4 (0.78, 2.51) | 0.62 (0.43, 0.9) |
| 0.27 (0.09, 0.8) | 0.4 (0.2, 0.81) | 0.58 (0.33, 1.01) | 0.8 (0.46, 1.37) | 0.85 (0.5, 1.46) | 18th | 1.05 (0.6, 1.84) | 1.2 (0.66, 2.17) | 0.53 (0.36, 0.79) |
| 0.26 (0.08, 0.77) | 0.38 (0.18, 0.78) | 0.55 (0.31, 0.98) | 0.76 (0.44, 1.31) | 0.81 (0.47, 1.41) | 0.95 (0.54, 1.67) | 21st | 1.14 (0.62, 2.1) | 0.51 (0.34, 0.76) |
| 0.23 (0.07, 0.69) | 0.33 (0.16, 0.7) | 0.48 (0.26, 0.88) | 0.66 (0.37, 1.19) | 0.71 (0.4, 1.28) | 0.83 (0.46, 1.51) | 0.88 (0.48, 1.61) | 24th | 0.45 (0.28, 0.69) |
| 0.51 (0.17, 1.4) | 0.75 (0.41, 1.36) | 1.08 (0.71, 1.63) | 1.49 (1.03, 2.17) | 1.61 (1.11, 2.33) | 1.88 (1.27, 2.77) | 1.97 (1.31, 2.97) | 2.25 (1.44, 3.54) | chemo |

Matrix of pairwise comparisons of Ate + chemo (shown as odds ratios and 95% confidence intervals).

| 3rd | 0.78 (0.27, 2.18) | 0.83 (0.31, 2.17) | 1.13 (0.42, 2.92) | 1.32 (0.49, 3.47) | 1.3 (0.47, 3.46) | 1.02 (0.36, 2.74) | 0.9 (0.32, 2.48) | 0.68 (0.27, 1.62) |
| --- | --- | --- | --- | --- | --- | --- | --- | --- |
| 1.28 (0.46, 3.72) | 6th | 1.06 (0.54, 2.09) | 1.44 (0.73, 2.83) | 1.69 (0.85, 3.35) | 1.66 (0.82, 3.38) | 1.3 (0.63, 2.69) | 1.15 (0.55, 2.43) | 0.86 (0.5, 1.5) |
| 1.21 (0.46, 3.26) | 0.95 (0.48, 1.87) | 9th | 1.36 (0.78, 2.39) | 1.6 (0.9, 2.84) | 1.57 (0.86, 2.86) | 1.23 (0.66, 2.3) | 1.09 (0.58, 2.08) | 0.82 (0.54, 1.22) |
| 0.89 (0.34, 2.38) | 0.69 (0.35, 1.37) | 0.74 (0.42, 1.29) | 12th | 1.17 (0.67, 2.08) | 1.15 (0.64, 2.1) | 0.9 (0.49, 1.69) | 0.8 (0.42, 1.53) | 0.6 (0.4, 0.89) |
| 0.75 (0.29, 2.06) | 0.59 (0.3, 1.17) | 0.63 (0.35, 1.11) | 0.85 (0.48, 1.49) | 15th | 0.98 (0.54, 1.8) | 0.77 (0.41, 1.44) | 0.68 (0.36, 1.31) | 0.51 (0.34, 0.77) |
| 0.77 (0.29, 2.11) | 0.6 (0.3, 1.21) | 0.64 (0.35, 1.16) | 0.87 (0.48, 1.57) | 1.02 (0.55, 1.86) | 18th | 0.78 (0.41, 1.5) | 0.7 (0.35, 1.36) | 0.52 (0.33, 0.81) |
| 0.98 (0.36, 2.75) | 0.77 (0.37, 1.58) | 0.81 (0.43, 1.51) | 1.11 (0.59, 2.05) | 1.3 (0.69, 2.44) | 1.27 (0.67, 2.45) | 21st | 0.89 (0.45, 1.76) | 0.66 (0.41, 1.06) |
| 1.11 (0.4, 3.12) | 0.87 (0.41, 1.81) | 0.92 (0.48, 1.74) | 1.25 (0.66, 2.36) | 1.47 (0.76, 2.78) | 1.44 (0.74, 2.82) | 1.13 (0.57, 2.25) | 24th | 0.75 (0.45, 1.22) |
| 1.48 (0.62, 3.7) | 1.16 (0.67, 2) | 1.22 (0.82, 1.84) | 1.67 (1.12, 2.48) | 1.96 (1.3, 2.96) | 1.92 (1.24, 3.02) | 1.51 (0.94, 2.44) | 1.34 (0.82, 2.22) | chemo |

Matrix of pairwise comparisons of Dur +Tre + chemo (shown as odds ratios and 95% confidence intervals).

| 3rd | 1.11 (0.54, 2.31) | 1.47 (0.74, 2.92) | 1.65 (0.83, 3.31) | 1.68 (0.84, 3.39) | 1.91 (0.95, 3.91) | 2.04 (1, 4.2) | 2.39 (1.14, 5.05) | 1.38 (0.77, 2.54) |
| --- | --- | --- | --- | --- | --- | --- | --- | --- |
| 0.9 (0.43, 1.86) | 6th | 1.31 (0.77, 2.25) | 1.49 (0.87, 2.53) | 1.51 (0.88, 2.6) | 1.72 (0.99, 3.01) | 1.83 (1.03, 3.26) | 2.15 (1.19, 3.96) | 1.24 (0.83, 1.88) |
| 0.68 (0.34, 1.35) | 0.76 (0.44, 1.29) | 9th | 1.13 (0.69, 1.83) | 1.15 (0.7, 1.88) | 1.3 (0.79, 2.18) | 1.39 (0.82, 2.36) | 1.64 (0.94, 2.87) | 0.95 (0.67, 1.34) |
| 0.6 (0.3, 1.21) | 0.67 (0.4, 1.15) | 0.89 (0.55, 1.44) | 12th | 1.01 (0.62, 1.67) | 1.16 (0.69, 1.94) | 1.24 (0.72, 2.1) | 1.45 (0.83, 2.55) | 0.84 (0.59, 1.18) |
| 0.6 (0.29, 1.19) | 0.66 (0.38, 1.14) | 0.87 (0.53, 1.44) | 0.99 (0.6, 1.61) | 15th | 1.14 (0.68, 1.93) | 1.21 (0.71, 2.09) | 1.43 (0.81, 2.54) | 0.83 (0.57, 1.18) |
| 0.52 (0.26, 1.05) | 0.58 (0.33, 1.01) | 0.77 (0.46, 1.27) | 0.86 (0.52, 1.45) | 0.88 (0.52, 1.48) | 18th | 1.07 (0.61, 1.86) | 1.25 (0.7, 2.25) | 0.73 (0.49, 1.06) |
| 0.49 (0.24, 1) | 0.55 (0.31, 0.97) | 0.72 (0.42, 1.23) | 0.81 (0.48, 1.38) | 0.82 (0.48, 1.41) | 0.94 (0.54, 1.63) | 21st | 1.18 (0.65, 2.14) | 0.68 (0.45, 1.02) |
| 0.42 (0.2, 0.87) | 0.46 (0.25, 0.84) | 0.61 (0.35, 1.07) | 0.69 (0.39, 1.2) | 0.7 (0.39, 1.23) | 0.8 (0.44, 1.42) | 0.85 (0.47, 1.54) | 24th | 0.58 (0.37, 0.89) |
| 0.72 (0.39, 1.3) | 0.8 (0.53, 1.2) | 1.06 (0.75, 1.49) | 1.19 (0.85, 1.68) | 1.21 (0.85, 1.74) | 1.38 (0.95, 2.03) | 1.47 (0.98, 2.21) | 1.73 (1.12, 2.7) | chemo |

Matrix of pairwise comparisons of Dur + chemo (shown as odds ratios and 95% confidence intervals).

| 3rd | 1 (0.47, 2.13) | 1.47 (0.72, 3.01) | 1.75 (0.86, 3.56) | 1.57 (0.77, 3.23) | 1.49 (0.71, 3.09) | 1.45 (0.69, 3.06) | 1.72 (0.8, 3.69) | 1.01 (0.54, 1.88) |
| --- | --- | --- | --- | --- | --- | --- | --- | --- |
| 1 (0.47, 2.14) | 6th | 1.46 (0.84, 2.55) | 1.75 (1.01, 3.02) | 1.57 (0.9, 2.75) | 1.49 (0.84, 2.62) | 1.45 (0.8, 2.61) | 1.72 (0.93, 3.22) | 1.01 (0.65, 1.54) |
| 0.68 (0.33, 1.4) | 0.68 (0.39, 1.19) | 9th | 1.2 (0.73, 1.95) | 1.07 (0.65, 1.77) | 1.01 (0.61, 1.71) | 0.99 (0.58, 1.69) | 1.17 (0.66, 2.08) | 0.69 (0.48, 0.98) |
| 0.57 (0.28, 1.17) | 0.57 (0.33, 0.99) | 0.84 (0.51, 1.37) | 12th | 0.9 (0.55, 1.47) | 0.85 (0.51, 1.42) | 0.83 (0.49, 1.41) | 0.98 (0.56, 1.74) | 0.57 (0.41, 0.81) |
| 0.64 (0.31, 1.3) | 0.64 (0.36, 1.11) | 0.93 (0.56, 1.54) | 1.12 (0.68, 1.83) | 15th | 0.95 (0.56, 1.59) | 0.92 (0.54, 1.59) | 1.09 (0.62, 1.95) | 0.64 (0.45, 0.91) |
| 0.67 (0.32, 1.4) | 0.67 (0.38, 1.19) | 0.99 (0.59, 1.65) | 1.18 (0.71, 1.96) | 1.06 (0.63, 1.77) | 18th | 0.97 (0.56, 1.7) | 1.16 (0.65, 2.08) | 0.68 (0.46, 0.98) |
| 0.69 (0.33, 1.45) | 0.69 (0.38, 1.25) | 1.01 (0.59, 1.73) | 1.21 (0.71, 2.06) | 1.08 (0.63, 1.86) | 1.03 (0.59, 1.79) | 21st | 1.19 (0.65, 2.18) | 0.7 (0.46, 1.04) |
| 0.58 (0.27, 1.25) | 0.58 (0.31, 1.08) | 0.85 (0.48, 1.51) | 1.02 (0.58, 1.8) | 0.91 (0.51, 1.62) | 0.86 (0.48, 1.55) | 0.84 (0.46, 1.54) | 24th | 0.59 (0.37, 0.92) |
| 0.99 (0.53, 1.85) | 0.99 (0.65, 1.53) | 1.46 (1.03, 2.07) | 1.74 (1.23, 2.45) | 1.56 (1.1, 2.23) | 1.48 (1.02, 2.15) | 1.44 (0.96, 2.16) | 1.71 (1.09, 2.68) | chemo |

Matrix of pairwise comparisons of Ipi + chemo (shown as odds ratios and 95% confidence intervals).

| 3rd | 1.48 (0.59, 3.83) | 1.35 (0.55, 3.36) | 1.3 (0.54, 3.23) | 1.48 (0.6, 3.73) | 1.5 (0.6, 3.84) | 1.31 (0.51, 3.4) | 1.77 (0.68, 4.71) | 1.21 (0.52, 2.91) |
| --- | --- | --- | --- | --- | --- | --- | --- | --- |
| 0.68 (0.26, 1.69) | 6th | 0.91 (0.59, 1.4) | 0.88 (0.57, 1.34) | 1 (0.64, 1.55) | 1.01 (0.63, 1.64) | 0.88 (0.53, 1.47) | 1.2 (0.69, 2.08) | 0.82 (0.58, 1.15) |
| 0.74 (0.3, 1.81) | 1.1 (0.72, 1.7) | 9th | 0.96 (0.67, 1.39) | 1.1 (0.75, 1.61) | 1.11 (0.73, 1.71) | 0.97 (0.61, 1.54) | 1.32 (0.8, 2.19) | 0.9 (0.69, 1.17) |
| 0.77 (0.31, 1.87) | 1.14 (0.74, 1.75) | 1.04 (0.72, 1.49) | 12th | 1.14 (0.78, 1.66) | 1.16 (0.76, 1.77) | 1 (0.63, 1.59) | 1.37 (0.83, 2.26) | 0.93 (0.72, 1.21) |
| 0.68 (0.27, 1.66) | 1 (0.65, 1.57) | 0.91 (0.62, 1.33) | 0.88 (0.6, 1.28) | 15^th^ | 1.02 (0.66, 1.58) | 0.88 (0.55, 1.41) | 1.2 (0.72, 2.01) | 0.82 (0.62, 1.08) |
| 0.67 (0.26, 1.65) | 0.99 (0.61, 1.59) | 0.9 (0.59, 1.37) | 0.87 (0.57, 1.32) | 0.98 (0.63, 1.52) | 18th | 0.87 (0.52, 1.44) | 1.18 (0.68, 2.04) | 0.81 (0.58, 1.13) |
| 0.77 (0.29, 1.96) | 1.14 (0.68, 1.9) | 1.03 (0.65, 1.64) | 1 (0.63, 1.58) | 1.13 (0.71, 1.82) | 1.15 (0.69, 1.91) | 21st | 1.36 (0.76, 2.44) | 0.93 (0.63, 1.36) |
| 0.56 (0.21, 1.46) | 0.84 (0.48, 1.45) | 0.76 (0.46, 1.26) | 0.73 (0.44, 1.21) | 0.83 (0.5, 1.39) | 0.85 (0.49, 1.46) | 0.74 (0.41, 1.32) | 24th | 0.68 (0.44, 1.05) |
| 0.82 (0.34, 1.93) | 1.22 (0.87, 1.73) | 1.11 (0.86, 1.44) | 1.07 (0.83, 1.38) | 1.22 (0.92, 1.61) | 1.24 (0.89, 1.73) | 1.08 (0.73, 1.58) | 1.46 (0.95, 2.26) | chemo |

Matrix of pairwise comparisons of Pem + chemo (shown as odds ratios and 95% confidence intervals).

| 3rd | 2.22 (0.98, 5.23) | 2.16 (0.99, 4.92) | 2.6 (1.19, 5.97) | 3.23 (1.47, 7.47) | 3.03 (1.35, 7.07) | 3.69 (1.6, 8.86) | 4.72 (1.97, 11.66) | 2.03 (1.02, 4.23) |
| --- | --- | --- | --- | --- | --- | --- | --- | --- |
| 0.45 (0.19, 1.02) | 6th | 0.97 (0.55, 1.72) | 1.17 (0.66, 2.08) | 1.46 (0.81, 2.63) | 1.36 (0.74, 2.51) | 1.66 (0.88, 3.17) | 2.12 (1.08, 4.22) | 0.92 (0.59, 1.41) |
| 0.46 (0.2, 1.01) | 1.03 (0.58, 1.81) | 9th | 1.21 (0.71, 2.05) | 1.5 (0.87, 2.58) | 1.4 (0.8, 2.47) | 1.71 (0.94, 3.11) | 2.18 (1.15, 4.19) | 0.94 (0.65, 1.36) |
| 0.38 (0.17, 0.84) | 0.85 (0.48, 1.51) | 0.83 (0.49, 1.4) | 12th | 1.24 (0.71, 2.13) | 1.16 (0.66, 2.05) | 1.41 (0.77, 2.6) | 1.81 (0.95, 3.46) | 0.78 (0.53, 1.14) |
| 0.31 (0.13, 0.68) | 0.69 (0.38, 1.24) | 0.67 (0.39, 1.15) | 0.81 (0.47, 1.4) | 15th | 0.94 (0.52, 1.68) | 1.14 (0.62, 2.12) | 1.46 (0.75, 2.85) | 0.63 (0.42, 0.93) |
| 0.33 (0.14, 0.74) | 0.73 (0.4, 1.35) | 0.71 (0.41, 1.25) | 0.86 (0.49, 1.52) | 1.07 (0.6, 1.91) | 18th | 1.22 (0.65, 2.3) | 1.55 (0.79, 3.08) | 0.67 (0.44, 1.03) |
| 0.27 (0.11, 0.62) | 0.6 (0.32, 1.14) | 0.58 (0.32, 1.06) | 0.71 (0.38, 1.29) | 0.88 (0.47, 1.62) | 0.82 (0.43, 1.54) | 21st | 1.28 (0.63, 2.6) | 0.55 (0.34, 0.88) |
| 0.21 (0.09, 0.51) | 0.47 (0.24, 0.93) | 0.46 (0.24, 0.87) | 0.55 (0.29, 1.06) | 0.69 (0.35, 1.33) | 0.64 (0.32, 1.27) | 0.78 (0.38, 1.59) | 24th | 0.43 (0.25, 0.72) |
| 0.49 (0.24, 0.98) | 1.09 (0.71, 1.69) | 1.06 (0.73, 1.54) | 1.28 (0.88, 1.87) | 1.59 (1.07, 2.38) | 1.49 (0.98, 2.29) | 1.82 (1.14, 2.92) | 2.31 (1.38, 3.97) | chemo |

Matrix of pairwise comparisons of Ser + chemo (shown as odds ratios and 95% confidence intervals).

| 3rd | 1.67 (0.61, 4.74) | 1.24 (0.48, 3.37) | 1.16 (0.45, 3.11) | 1.39 (0.54, 3.72) | 1.17 (0.46, 3.13) | 0.73 (0.3, 1.83) |
| --- | --- | --- | --- | --- | --- | --- |
| 0.6 (0.21, 1.65) | 6th | 0.75 (0.4, 1.4) | 0.7 (0.38, 1.28) | 0.84 (0.46, 1.54) | 0.71 (0.39, 1.29) | 0.44 (0.27, 0.72) |
| 0.8 (0.3, 2.08) | 1.34 (0.71, 2.53) | 9th | 0.94 (0.55, 1.58) | 1.12 (0.67, 1.88) | 0.95 (0.56, 1.59) | 0.59 (0.4, 0.86) |
| 0.86 (0.32, 2.2) | 1.43 (0.78, 2.63) | 1.07 (0.63, 1.8) | 12^th^ | 1.2 (0.73, 1.96) | 1.01 (0.62, 1.66) | 0.63 (0.44, 0.89) |
| 0.72 (0.27, 1.84) | 1.19 (0.65, 2.19) | 0.89 (0.53, 1.49) | 0.84 (0.51, 1.36) | 15th | 0.84 (0.51, 1.38) | 0.52 (0.37, 0.74) |
| 0.85 (0.32, 2.16) | 1.41 (0.78, 2.58) | 1.06 (0.63, 1.77) | 0.99 (0.6, 1.63) | 1.19 (0.73, 1.94) | 18th | 0.62 (0.44, 0.87) |
| 1.37 (0.55, 3.29) | 2.29 (1.4, 3.75) | 1.71 (1.16, 2.51) | 1.6 (1.12, 2.28) | 1.92 (1.36, 2.71) | 1.62 (1.14, 2.29) | chemo |

Matrix of pairwise comparisons of Ben + Anl + chemo (shown as odds ratios and 95% confidence intervals).

| 3rd | 1.25 (0.27, 5.5) | 1.04 (0.23, 4.34) | 1.37 (0.31, 5.62) | 1.8 (0.41, 7.37) | 2.45 (0.55, 10.19) | 1.98 (0.44, 8.18) | 1.78 (0.4, 7.43) | 0.79 (0.19, 3.1) |
| --- | --- | --- | --- | --- | --- | --- | --- | --- |
| 0.8 (0.18, 3.75) | 6th | 0.83 (0.41, 1.67) | 1.1 (0.56, 2.15) | 1.44 (0.73, 2.84) | 1.97 (0.98, 3.9) | 1.58 (0.79, 3.15) | 1.43 (0.71, 2.85) | 0.64 (0.35, 1.12) |
| 0.96 (0.23, 4.29) | 1.2 (0.6, 2.44) | 9th | 1.32 (0.77, 2.27) | 1.73 (1.01, 2.98) | 2.36 (1.36, 4.13) | 1.9 (1.09, 3.32) | 1.71 (0.98, 3.03) | 0.76 (0.51, 1.14) |
| 0.73 (0.18, 3.23) | 0.91 (0.46, 1.79) | 0.76 (0.44, 1.3) | 12th | 1.31 (0.78, 2.19) | 1.79 (1.06, 3.01) | 1.44 (0.85, 2.44) | 1.3 (0.77, 2.21) | 0.58 (0.4, 0.83) |
| 0.55 (0.14, 2.46) | 0.69 (0.35, 1.37) | 0.58 (0.34, 0.99) | 0.76 (0.46, 1.27) | 15th | 1.36 (0.81, 2.31) | 1.09 (0.64, 1.86) | 0.99 (0.58, 1.69) | 0.44 (0.3, 0.63) |
| 0.41 (0.1, 1.8) | 0.51 (0.26, 1.02) | 0.42 (0.24, 0.73) | 0.56 (0.33, 0.94) | 0.73 (0.43, 1.24) | 18th | 0.8 (0.47, 1.38) | 0.73 (0.42, 1.25) | 0.32 (0.22, 0.47) |
| 0.51 (0.12, 2.26) | 0.63 (0.32, 1.27) | 0.53 (0.3, 0.92) | 0.7 (0.41, 1.18) | 0.91 (0.54, 1.55) | 1.25 (0.72, 2.14) | 21st | 0.91 (0.52, 1.57) | 0.4 (0.27, 0.59) |
| 0.56 (0.13, 2.5) | 0.7 (0.35, 1.41) | 0.58 (0.33, 1.03) | 0.77 (0.45, 1.3) | 1.01 (0.59, 1.72) | 1.37 (0.8, 2.38) | 1.1 (0.64, 1.91) | 24th | 0.44 (0.3, 0.65) |
| 1.27 (0.32, 5.36) | 1.57 (0.89, 2.83) | 1.31 (0.88, 1.97) | 1.74 (1.21, 2.5) | 2.28 (1.58, 3.29) | 3.1 (2.14, 4.55) | 2.49 (1.7, 3.68) | 2.25 (1.53, 3.34) | chemo |

Matrix of pairwise comparisons of Tis + chemo (shown as odds ratios and 95% confidence intervals).

| 3rd | 1.97 (0.7, 5.95) | 2.2 (0.85, 6.19) | 2.4 (0.93, 6.57) | 2.92 (1.14, 8.04) | 2.92 (1.15, 8.06) | 3.12 (1.22, 8.64) | 3.56 (1.36, 10.05) | 2 (0.85, 5.14) |
| --- | --- | --- | --- | --- | --- | --- | --- | --- |
| 0.51 (0.17, 1.42) | 6th | 1.12 (0.55, 2.26) | 1.21 (0.61, 2.41) | 1.48 (0.75, 2.91) | 1.48 (0.74, 2.95) | 1.58 (0.79, 3.18) | 1.81 (0.89, 3.7) | 1.01 (0.57, 1.8) |
| 0.45 (0.16, 1.18) | 0.9 (0.44, 1.82) | 9th | 1.09 (0.62, 1.92) | 1.33 (0.76, 2.32) | 1.32 (0.75, 2.33) | 1.42 (0.8, 2.53) | 1.62 (0.9, 2.93) | 0.91 (0.6, 1.38) |
| 0.42 (0.15, 1.07) | 0.83 (0.42, 1.63) | 0.92 (0.52, 1.62) | 12th | 1.22 (0.72, 2.06) | 1.22 (0.71, 2.09) | 1.3 (0.75, 2.26) | 1.49 (0.85, 2.62) | 0.84 (0.57, 1.22) |
| 0.34 (0.12, 0.87) | 0.68 (0.34, 1.34) | 0.75 (0.43, 1.31) | 0.82 (0.48, 1.39) | 15th | 1 (0.59, 1.7) | 1.07 (0.62, 1.84) | 1.22 (0.7, 2.13) | 0.69 (0.47, 0.99) |
| 0.34 (0.12, 0.87) | 0.68 (0.34, 1.35) | 0.76 (0.43, 1.33) | 0.82 (0.48, 1.4) | 1 (0.59, 1.7) | 18th | 1.07 (0.62, 1.86) | 1.22 (0.7, 2.15) | 0.69 (0.47, 1) |
| 0.32 (0.12, 0.82) | 0.63 (0.31, 1.27) | 0.71 (0.39, 1.25) | 0.77 (0.44, 1.33) | 0.94 (0.54, 1.61) | 0.93 (0.54, 1.62) | 21st | 1.14 (0.64, 2.03) | 0.64 (0.43, 0.95) |
| 0.28 (0.1, 0.73) | 0.55 (0.27, 1.12) | 0.62 (0.34, 1.11) | 0.67 (0.38, 1.18) | 0.82 (0.47, 1.43) | 0.82 (0.46, 1.44) | 0.87 (0.49, 1.55) | 24th | 0.56 (0.37, 0.85) |
| 0.5 (0.19, 1.18) | 0.99 (0.55, 1.75) | 1.1 (0.73, 1.67) | 1.19 (0.82, 1.75) | 1.46 (1.01, 2.11) | 1.45 (1, 2.14) | 1.56 (1.05, 2.33) | 1.78 (1.17, 2.73) | chemo |

Matrix of pairwise comparisons of Tor + chemo (shown as odds ratios and 95% confidence intervals)

| 3rd | 1.8 (0.46, 7.33) | 1.64 (0.45, 6.34) | 1.73 (0.49, 6.53) | 1.74 (0.49, 6.54) | 1.67 (0.46, 6.31) | 1.76 (0.48, 6.69) | 1.71 (0.47, 6.6) | 1.22 (0.36, 4.36) |
| --- | --- | --- | --- | --- | --- | --- | --- | --- |
| 0.56 (0.14, 2.19) | 6th | 0.92 (0.44, 1.92) | 0.97 (0.47, 1.95) | 0.97 (0.47, 1.95) | 0.93 (0.45, 1.9) | 0.98 (0.47, 2.04) | 0.96 (0.45, 2.02) | 0.68 (0.37, 1.23) |
| 0.61 (0.16, 2.24) | 1.09 (0.52, 2.29) | 9th | 1.05 (0.59, 1.89) | 1.06 (0.59, 1.88) | 1.01 (0.56, 1.84) | 1.07 (0.58, 1.96) | 1.05 (0.55, 1.95) | 0.74 (0.47, 1.15) |
| 0.58 (0.15, 2.05) | 1.03 (0.51, 2.12) | 0.95 (0.53, 1.71) | 12th | 1 (0.59, 1.71) | 0.96 (0.56, 1.66) | 1.02 (0.58, 1.79) | 0.99 (0.55, 1.79) | 0.7 (0.48, 1.03) |
| 0.58 (0.15, 2.06) | 1.03 (0.51, 2.11) | 0.95 (0.53, 1.7) | 1 (0.59, 1.71) | 15th | 0.96 (0.56, 1.65) | 1.01 (0.58, 1.77) | 0.99 (0.55, 1.78) | 0.7 (0.48, 1.02) |
| 0.6 (0.16, 2.16) | 1.08 (0.53, 2.21) | 0.99 (0.54, 1.79) | 1.04 (0.6, 1.79) | 1.04 (0.6, 1.79) | 18th | 1.05 (0.59, 1.87) | 1.03 (0.57, 1.87) | 0.73 (0.49, 1.08) |
| 0.57 (0.15, 2.08) | 1.02 (0.49, 2.13) | 0.94 (0.51, 1.72) | 0.98 (0.56, 1.73) | 0.99 (0.56, 1.73) | 0.95 (0.53, 1.68) | 21st | 0.98 (0.53, 1.81) | 0.69 (0.45, 1.05) |
| 0.58 (0.15, 2.14) | 1.04 (0.5, 2.22) | 0.96 (0.51, 1.81) | 1.01 (0.56, 1.81) | 1.01 (0.56, 1.82) | 0.97 (0.53, 1.76) | 1.02 (0.55, 1.9) | 24th | 0.71 (0.45, 1.11) |
| 0.82 (0.23, 2.79) | 1.47 (0.81, 2.7) | 1.35 (0.87, 2.11) | 1.42 (0.97, 2.08) | 1.43 (0.98, 2.08) | 1.37 (0.93, 2.03) | 1.44 (0.95, 2.2) | 1.41 (0.9, 2.22) | chemo |

Matrix of pairwise comparisons of Anl + chemo (shown as odds ratios and 95% confidence intervals)

| 3rd | 1.38 (0.46, 4.17) | 1.2 (0.43, 3.4) | 1.21 (0.43, 3.35) | 1.37 (0.49, 3.81) | 1.52 (0.54, 4.27) | 1.22 (0.43, 3.46) | 0.93 (0.32, 2.63) | 1.03 (0.39, 2.68) |
| --- | --- | --- | --- | --- | --- | --- | --- | --- |
| 0.73 (0.24, 2.18) | 6th | 0.88 (0.45, 1.7) | 0.88 (0.46, 1.67) | 0.99 (0.51, 1.91) | 1.11 (0.57, 2.15) | 0.89 (0.45, 1.75) | 0.67 (0.34, 1.34) | 0.75 (0.43, 1.28) |
| 0.83 (0.29, 2.35) | 1.14 (0.59, 2.25) | 9th | 1 (0.59, 1.69) | 1.13 (0.66, 1.93) | 1.27 (0.73, 2.18) | 1.02 (0.58, 1.78) | 0.77 (0.44, 1.35) | 0.85 (0.58, 1.25) |
| 0.83 (0.3, 2.32) | 1.14 (0.6, 2.19) | 1 (0.59, 1.69) | 12th | 1.13 (0.68, 1.88) | 1.26 (0.75, 2.14) | 1.02 (0.6, 1.74) | 0.77 (0.44, 1.32) | 0.85 (0.6, 1.21) |
| 0.73 (0.26, 2.06) | 1.01 (0.52, 1.95) | 0.88 (0.52, 1.51) | 0.88 (0.53, 1.47) | 15th | 1.12 (0.66, 1.9) | 0.9 (0.52, 1.55) | 0.68 (0.39, 1.18) | 0.75 (0.52, 1.08) |
| 0.66 (0.23, 1.85) | 0.9 (0.46, 1.77) | 0.79 (0.46, 1.37) | 0.79 (0.47, 1.33) | 0.9 (0.53, 1.52) | 18th | 0.81 (0.46, 1.41) | 0.61 (0.35, 1.07) | 0.67 (0.46, 0.99) |
| 0.82 (0.29, 2.31) | 1.12 (0.57, 2.22) | 0.98 (0.56, 1.72) | 0.98 (0.57, 1.68) | 1.11 (0.64, 1.92) | 1.24 (0.71, 2.17) | 21st | 0.75 (0.42, 1.35) | 0.83 (0.56, 1.25) |
| 1.08 (0.38, 3.09) | 1.49 (0.75, 2.95) | 1.3 (0.74, 2.29) | 1.31 (0.76, 2.25) | 1.47 (0.85, 2.56) | 1.65 (0.93, 2.9) | 1.33 (0.74, 2.36) | 24th | 1.11 (0.73, 1.67) |
| 0.97 (0.37, 2.56) | 1.34 (0.78, 2.32) | 1.18 (0.8, 1.73) | 1.18 (0.83, 1.68) | 1.33 (0.93, 1.91) | 1.49 (1.01, 2.19) | 1.2 (0.8, 1.79) | 0.9 (0.6, 1.37) | chemo |

Matrix of pairwise comparisons of Bev + chemo (shown as odds ratios and 95% confidence intervals)

| 3rd | 1.09 (0.4, 3) | 1.07 (0.41, 2.78) | 1.38 (0.51, 3.74) | 1.6 (0.57, 4.55) | 1.42 (0.47, 4.3) | 1.4 (0.44, 4.42) | 1.56 (0.47, 5.43) | 0.87 (0.39, 1.91) |
| --- | --- | --- | --- | --- | --- | --- | --- | --- |
| 0.92 (0.33, 2.5) | 6th | 0.98 (0.42, 2.23) | 1.26 (0.53, 3.03) | 1.46 (0.59, 3.71) | 1.3 (0.48, 3.57) | 1.28 (0.45, 3.7) | 1.43 (0.47, 4.51) | 0.8 (0.43, 1.5) |
| 0.94 (0.36, 2.45) | 1.02 (0.45, 2.37) | 9th | 1.3 (0.57, 2.97) | 1.5 (0.64, 3.64) | 1.33 (0.52, 3.5) | 1.31 (0.48, 3.62) | 1.47 (0.5, 4.47) | 0.82 (0.47, 1.42) |
| 0.72 (0.27, 1.96) | 0.79 (0.33, 1.87) | 0.77 (0.34, 1.75) | 12th | 1.16 (0.47, 2.91) | 1.03 (0.39, 2.76) | 1.01 (0.36, 2.86) | 1.13 (0.38, 3.54) | 0.63 (0.34, 1.16) |
| 0.62 (0.22, 1.76) | 0.68 (0.27, 1.71) | 0.67 (0.27, 1.57) | 0.86 (0.34, 2.13) | 15th | 0.88 (0.32, 2.49) | 0.87 (0.3, 2.55) | 0.97 (0.31, 3.15) | 0.55 (0.27, 1.06) |
| 0.7 (0.23, 2.13) | 0.77 (0.28, 2.08) | 0.75 (0.29, 1.93) | 0.97 (0.36, 2.59) | 1.13 (0.4, 3.14) | 18th | 0.98 (0.31, 3.08) | 1.1 (0.33, 3.76) | 0.62 (0.28, 1.33) |
| 0.72 (0.23, 2.27) | 0.78 (0.27, 2.22) | 0.77 (0.28, 2.07) | 0.99 (0.35, 2.8) | 1.15 (0.39, 3.35) | 1.02 (0.33, 3.23) | 21st | 1.12 (0.32, 4) | 0.63 (0.27, 1.43) |
| 0.64 (0.18, 2.15) | 0.7 (0.22, 2.14) | 0.68 (0.22, 1.98) | 0.88 (0.28, 2.65) | 1.03 (0.32, 3.23) | 0.91 (0.27, 3.06) | 0.89 (0.25, 3.09) | 24th | 0.56 (0.21, 1.39) |
| 1.14 (0.52, 2.54) | 1.25 (0.67, 2.35) | 1.22 (0.7, 2.12) | 1.58 (0.86, 2.92) | 1.83 (0.94, 3.65) | 1.62 (0.75, 3.6) | 1.59 (0.7, 3.72) | 1.78 (0.72, 4.73) | chemo |

Matrix of pairwise comparisons of Soc + chemo (shown as odds ratios and 95% confidence intervals)

| 3rd | 0.98 (0.32, 2.9) | 0.85 (0.29, 2.34) | 0.98 (0.34, 2.68) | 1.23 (0.43, 3.37) | 1.01 (0.35, 2.8) | 0.82 (0.28, 2.3) | 2.99 (0.94, 9.18) | 0.71 (0.26, 1.81) |
| --- | --- | --- | --- | --- | --- | --- | --- | --- |
| 1.02 (0.35, 3.08) | 6th | 0.86 (0.45, 1.65) | 1 (0.53, 1.88) | 1.25 (0.65, 2.37) | 1.03 (0.53, 1.99) | 0.83 (0.42, 1.64) | 3.04 (1.37, 6.91) | 0.72 (0.42, 1.22) |
| 1.18 (0.43, 3.41) | 1.16 (0.61, 2.23) | 9th | 1.16 (0.68, 1.97) | 1.45 (0.85, 2.48) | 1.2 (0.69, 2.07) | 0.97 (0.54, 1.72) | 3.52 (1.74, 7.43) | 0.84 (0.57, 1.24) |
| 1.02 (0.37, 2.91) | 1 (0.53, 1.9) | 0.86 (0.51, 1.46) | 12th | 1.25 (0.75, 2.08) | 1.03 (0.61, 1.75) | 0.83 (0.48, 1.45) | 3.03 (1.52, 6.27) | 0.72 (0.51, 1.03) |
| 0.81 (0.3, 2.33) | 0.8 (0.42, 1.53) | 0.69 (0.4, 1.17) | 0.8 (0.48, 1.33) | 15th | 0.82 (0.48, 1.41) | 0.67 (0.38, 1.17) | 2.42 (1.21, 5.06) | 0.58 (0.4, 0.83) |
| 0.99 (0.36, 2.84) | 0.97 (0.5, 1.87) | 0.84 (0.48, 1.44) | 0.97 (0.57, 1.64) | 1.21 (0.71, 2.07) | 18th | 0.81 (0.45, 1.44) | 2.95 (1.45, 6.23) | 0.7 (0.47, 1.03) |
| 1.22 (0.43, 3.57) | 1.2 (0.61, 2.38) | 1.04 (0.58, 1.85) | 1.2 (0.69, 2.09) | 1.5 (0.85, 2.64) | 1.24 (0.69, 2.2) | 21st | 3.64 (1.75, 7.86) | 0.87 (0.57, 1.33) |
| 0.33 (0.11, 1.06) | 0.33 (0.14, 0.73) | 0.28 (0.13, 0.58) | 0.33 (0.16, 0.66) | 0.41 (0.2, 0.83) | 0.34 (0.16, 0.69) | 0.27 (0.13, 0.57) | 24th | 0.24 (0.13, 0.43) |
| 1.41 (0.55, 3.78) | 1.38 (0.82, 2.36) | 1.19 (0.81, 1.76) | 1.38 (0.97, 1.98) | 1.73 (1.2, 2.5) | 1.43 (0.97, 2.11) | 1.15 (0.75, 1.77) | 4.19 (2.33, 7.98) | chemo |

| Table S6 Comparison of the PFS rate of combined therapy to standard chemotherapy at t the 1st, 2nd, 3rd, 4th, 5th, 6th, 7th, 8th, 9th, 10th, 11th and 12th month, ORs and 95%CI. | | | | | | | | | | | | | |
| --- | --- | --- | --- | --- | --- | --- | --- | --- | --- | --- | --- | --- | --- |
| Ti me (months) | Ade + chemo | Ate + chemo | Dur + Tre +chemo | Dur + chemo | Ipi + chemo | Pem + chemo | Ser + chemo | Ben + Anl +chemo | Tis + chemo | Tor + chemo | Anl + chemo | Soc + chemo | Bev + chemo |
| 1st | 2.32 (0.18, 70.51) | 0.8  (0.31, 2) | 1 (0.4, 2.5) | 0.8 (0.31, 2) | - | 0.77 (0.27, 2.16) | 1.58  (0.37, 6.23) | 1.41 (0.55,3.74) | 0.99 (0.17, 5.83) | 3.92 (0.43,113.15) | 0.99 (0.22, 4.44) | 5.26 (0.65, 148.52) | 1.18 (0.34, 4.27) |
| 2nd | 0.56 (0.26, 1.12) | 0.75  (0.38, 1.45) | 1.1  (0.66, 1.84) | 1.29  (0.76, 2.2) | - | 0.92 (0.49, 1.7) | 1.94 (0.95, 3.97) | 1.57  (0.87, 2.9) | 0.66  (0.32, 1.34) | 0.79  (0.34, 1.79) | 2.24  (0.97, 5.64) | 2.76  (1.17, 7.33) | 1.28 (0.55, 3.08) |
| 3rd | 0.81  (0.46, 1.42) | 0.77  (0.47, 1.26) | 1.04  (0.69, 1.57) | 1.47  (0.95, 2.29) | 1  (0.66, 1.53) | 0.82  (0.5, 1.32) | 2.14  (1.29, 3.53) | 1.56  (0.98, 2.51) | 0.83  (0.49, 1.39) | 1.26  (0.72, 2.25) | 1.74  (0.98, 3.18) | 2.8  (1.39, 6.06) | 1.39  (0.71, 2.76) |
| 4th | 0.99  (0.61, 1.63) | 1.02  (0.66, 1.59) | 0.89  (0.6, 1.3) | 1.15 (0.77, 1.71) | 1.26 (0.93, 1.71) | 1.04  (0.68, 1.58) | 2.03  (1.29, 3.17) | 1.74 (1.15, 2.68) | 0.81  (0.52, 1.25) | 1.06 (0.63, 1.79) | 1.69  (1.03, 2.8) | 2.15  (1.29, 3.67) | 1.43  (0.75, 2.77) |
| 5th | 1.29  (0.87, 1.92) | 1.45 (0.98, 2.17) | 0.86  (0.61, 1.2) | 0.92 (0.66, 1.29) | 1.28  (0.99, 1.66) | 1.35  (0.93, 1.97) | 2.28  (1.6, 3.26) | 4.08  (2.8, 5.95) | 1.73  (1.19, 2.51) | 1.21  (0.81, 1.79) | 3.61 (2.5, 5.26) | 2.02  (1.42, 2.89) | 1.43 (0.81, 2.54) |
| 6th | 1.64 (1.13, 2.38) | 1.46 (0.93, 2.31) | 0.89 (0.63, 1.25) | 0.98 (0.7, 1.37) | 1.28  (0.96, 1.71) | 1.69 (1.12, 2.56) | 2.6 (1.82, 3.72) | 6.22  (3.98, 10) | 2.65  (1.72, 4.13) | 1.26 (0.86, 1.86) | 4.93  (3.26, 7.59) | 2.57 (1.74, 3.8) | 1.55  (0.89, 2.71) |
| 7th | 1.91  (1.29, 2.84) | 1.81 (1.09, 3.03) | 1.06 (0.73, 1.55) | 1.16  (0.8, 1.7) | 1.17 (0.85, 1.62) | 2.08 (1.32, 3.35) | 2.74 (1.9, 3.98) | 6.68  (3.84, 12.33) | 3.24 (2.02, 5.33) | 1.68 (1.12, 2.55) | 4.63 (2.95, 7.4) | 2.73  (1.77, 4.28) | 1.62 (0.92, 2.89) |
| 8th | 2.23 (1.45, 3.45) | 1.83  (1.07, 3.2) | 1.33 (0.89, 2.01) | 1.65 (1.11, 2.49) | 1.26 (0.89, 1.79) | 2.5 (1.51, 4.27) | 2.62 (1.8, 3.82) | 7.78  (4.3, 15.12) | 3.92 (2.34, 6.79) | 1.93 (1.24, 3.04) | 5.37  (3.26, 9.34) | 2.47  (1.56, 3.96) | 1.59 (0.87, 2.96) |
| 9th | 2.7 (1.65, 4.49) | 1.48  (0.83, 2.65) | 1.64 (1.05, 2.6) | 1.62 (1.04, 2.54) | 1.61 (1.07, 2.47) | 2.4  (1.34, 4.45) | 2.35  (1.62, 3.46) | 15.2 (6.4, 45.68) | 4.78 (2.66, 9.1) | 3.19 (1.88, 5.57) | 6.35 (3.34, 13.25) | 2.4  (1.45, 4.05) | 1.64 (0.84, 3.27) |
| 10th | 3.26  (1.86, 5.92) | 1.99 (1.05, 3.88) | 1.71 (1.05, 2.79) | 1.79  (1.12, 2.91) | 1.79 (1.15, 2.84) | 2.66  (1.43, 5.2) | 2.28 (1.56, 3.38) | 18.12 (6.25, 82.3) | 4.8 (2.59, 9.54) | 3.3 (1.86, 6.13) | 5.31  (2.61, 12.12) | 3.79 (2.1, 7.24) | 1.53 (0.75, 3.18) |
| 11th | 3.39 (1.91, 6.28) | 1.87  (0.96, 3.75) | 2.41  (1.42, 4.21) | 2.54 (1.5, 4.39) | 1.53 (0.96, 2.5) | 3.61 (1.83, 7.74) | 2.37 (1.61, 3.54) | 28.34 (7.99, 200.28) | 4.67 (2.52, 9.34) | 3.26 (1.81, 6.17) | 7.73 (3.42, 21.09) | 4.19 (2.26, 8.29) | 1.48 (0.7, 3.22) |
| 12th | 3.85 (2.1, 7.54) | 2.55  (1.16, 6.02) | 3.84 (2.09, 7.48) | 4.02  (2.21, 7.83) | 1.47 (0.85, 2.58) | 5.08  (2.3, 12.98) | 2.1 (1.42, 3.16) | 44.9  (7.93, 1249) | 5.91  (3.01, 12.93) | 4.45 (2.36, 9.14) | 6.08 (2.63, 16.86) | 4.2 (2.26, 8.34) | 1.66 (0.75, 3.8) |
| PFS, progression-free survival; OR, Odds ratio; HR, Hazard ratio; CI, confidence interval; Ade + chemo, adebrelimab + chemotherapy; Ate + chemo, atezolizumab + chemotherapy; Dur + Tre + chemo, durvalumab + tremelimuamb + chemotherapy; Dur + chemo, durvalumab + chemotherapy; Ipi + chemo, ipilimumab + chemotherapy; Pem + chemo, pembrolizumab + chemotherapy; Ser + chemo, serplulimab + chemotherapy; Ben + Anl + chemo, benmelstobart + anlotinib + chemotherapy; Tis + chemo, tislelizumab + chemotherapy; Tor + chemo, toripalimab + chemotherapy; Anl + chemo, anlotinib + chemotherapy; Soc + chemo, socazolimab + chemotherapy; Bev + chemo, bevacizumab + chemotherapy. | | | | | | | | | | | | | |

Table S7 Matrix of pairwise comparisons of regimens on PFS.

Matrix of pairwise comparisons of Ade + chemo (shown as odds ratios and 95% confidence intervals).

| 1st | 0.24 (0.01, 3.32) | 0.35 (0.01, 4.69) | 0.42 (0.01, 5.71) | 0.55 (0.02, 7.22) | 0.7 (0.02, 9.24) | 0.82 (0.03, 10.76) | 0.96 (0.03, 12.72) | 1.15 (0.04, 15.69) | 1.4 (0.04, 19.05) | 1.46 (0.05, 20.02) | 1.66 (0.05, 23.07) | 0.43 (0.01, 5.49) |
| --- | --- | --- | --- | --- | --- | --- | --- | --- | --- | --- | --- | --- |
| 4.25 (0.3, 134.64) | 2nd | 1.46 (0.59, 3.68) | 1.79 (0.75, 4.35) | 2.32 (1.04, 5.38) | 2.96 (1.33, 6.78) | 3.43 (1.53, 7.98) | 4.01 (1.76, 9.47) | 4.87 (2.05, 11.92) | 5.9 (2.37, 15.2) | 6.12 (2.45, 15.89) | 6.97 (2.74, 18.6) | 1.8 (0.89, 3.8) |
| 2.89 (0.21, 91.04) | 0.69 (0.27, 1.7) | 3rd | 1.22 (0.58, 2.6) | 1.59 (0.8, 3.18) | 2.03 (1.04, 3.98) | 2.36 (1.19, 4.71) | 2.75 (1.36, 5.61) | 3.34 (1.58, 7.13) | 4.04 (1.81, 9.16) | 4.19 (1.89, 9.59) | 4.78 (2.08, 11.41) | 1.24 (0.7, 2.18) |
| 2.37 (0.17, 73.57) | 0.56 (0.23, 1.34) | 0.82 (0.38, 1.73) | 4th | 1.3 (0.69, 2.45) | 1.66 (0.89, 3.08) | 1.93 (1.03, 3.63) | 2.25 (1.17, 4.36) | 2.72 (1.36, 5.59) | 3.31 (1.56, 7.12) | 3.43 (1.6, 7.41) | 3.89 (1.77, 8.95) | 1.01 (0.61, 1.65) |
| 1.81 (0.14, 55.79) | 0.43 (0.19, 0.96) | 0.63 (0.31, 1.25) | 0.77 (0.41, 1.45) | 5th | 1.27 (0.74, 2.2) | 1.48 (0.85, 2.6) | 1.73 (0.96, 3.12) | 2.09 (1.11, 4) | 2.54 (1.27, 5.14) | 2.63 (1.31, 5.48) | 2.99 (1.44, 6.54) | 0.78 (0.52, 1.16) |
| 1.42 (0.11, 43.65) | 0.34 (0.15, 0.75) | 0.49 (0.25, 0.96) | 0.6 (0.32, 1.12) | 0.78 (0.46, 1.35) | 6th | 1.16 (0.68, 2) | 1.36 (0.77, 2.42) | 1.64 (0.88, 3.09) | 1.99 (1.02, 3.99) | 2.06 (1.04, 4.2) | 2.34 (1.15, 5.06) | 0.61 (0.42, 0.88) |
| 1.23 (0.09, 38.29) | 0.29 (0.13, 0.65) | 0.42 (0.21, 0.84) | 0.52 (0.28, 0.97) | 0.67 (0.38, 1.18) | 0.86 (0.5, 1.48) | 7th | 1.17 (0.65, 2.09) | 1.41 (0.75, 2.71) | 1.71 (0.86, 3.47) | 1.78 (0.88, 3.66) | 2.02 (0.98, 4.38) | 0.52 (0.35, 0.78) |
| 1.04 (0.08, 32.73) | 0.25 (0.11, 0.57) | 0.36 (0.18, 0.73) | 0.44 (0.23, 0.86) | 0.58 (0.32, 1.04) | 0.74 (0.41, 1.3) | 0.86 (0.48, 1.54) | 8th | 1.21 (0.63, 2.36) | 1.47 (0.71, 3.04) | 1.52 (0.74, 3.21) | 1.73 (0.81, 3.82) | 0.45 (0.29, 0.69) |
| 0.87 (0.06, 26.67) | 0.21 (0.08, 0.49) | 0.3 (0.14, 0.63) | 0.37 (0.18, 0.74) | 0.48 (0.25, 0.9) | 0.61 (0.32, 1.13) | 0.71 (0.37, 1.33) | 0.83 (0.42, 1.59) | 9th | 1.21 (0.57, 2.61) | 1.26 (0.58, 2.75) | 1.43 (0.64, 3.28) | 0.37 (0.22, 0.6) |
| 0.71 (0.05, 22.44) | 0.17 (0.07, 0.42) | 0.25 (0.11, 0.55) | 0.3 (0.14, 0.64) | 0.39 (0.19, 0.79) | 0.5 (0.25, 0.99) | 0.59 (0.29, 1.16) | 0.68 (0.33, 1.4) | 0.83 (0.38, 1.77) | 10th | 1.04 (0.45, 2.39) | 1.18 (0.5, 2.85) | 0.31 (0.17, 0.54) |
| 0.69 (0.05, 21.85) | 0.16 (0.06, 0.41) | 0.24 (0.1, 0.53) | 0.29 (0.13, 0.63) | 0.38 (0.18, 0.77) | 0.48 (0.24, 0.96) | 0.56 (0.27, 1.14) | 0.66 (0.31, 1.35) | 0.79 (0.36, 1.71) | 0.96 (0.42, 2.21) | 11th | 1.14 (0.48, 2.73) | 0.3 (0.16, 0.52) |
| 0.6 (0.04, 19.32) | 0.14 (0.05, 0.37) | 0.21 (0.09, 0.48) | 0.26 (0.11, 0.56) | 0.33 (0.15, 0.69) | 0.43 (0.2, 0.87) | 0.5 (0.23, 1.03) | 0.58 (0.26, 1.23) | 0.7 (0.3, 1.56) | 0.85 (0.35, 2.01) | 0.88 (0.37, 2.09) | 12th | 0.26 (0.13, 0.48) |
| 2.32 (0.18, 70.51) | 0.56 (0.26, 1.12) | 0.81 (0.46, 1.42) | 0.99 (0.61, 1.63) | 1.29 (0.87, 1.92) | 1.64 (1.13, 2.38) | 1.91 (1.29, 2.84) | 2.23 (1.45, 3.45) | 2.7 (1.65, 4.49) | 3.26 (1.86, 5.92) | 3.39 (1.91, 6.28) | 3.85 (2.1, 7.54) | chemo |

Matrix of pairwise comparisons of Ate + chemo (shown as odds ratios and 95% confidence intervals).

| 1st | 0.94 (0.3, 2.96) | 0.96 (0.34, 2.8) | 1.28 (0.47, 3.6) | 1.82 (0.67, 5.04) | 1.83 (0.66, 5.24) | 2.26 (0.8, 6.67) | 2.3 (0.79, 6.81) | 1.85 (0.63, 5.58) | 2.5 (0.82, 7.84) | 2.35 (0.75, 7.58) | 3.21 (0.95, 11.39) | 1.25 (0.5, 3.21) |
| --- | --- | --- | --- | --- | --- | --- | --- | --- | --- | --- | --- | --- |
| 1.06 (0.34, 3.28) | 2nd | 1.02 (0.45, 2.33) | 1.36 (0.62, 3.03) | 1.93 (0.9, 4.23) | 1.94 (0.88, 4.38) | 2.4 (1.05, 5.6) | 2.43 (1.04, 5.8) | 1.96 (0.82, 4.76) | 2.64 (1.06, 6.78) | 2.49 (0.97, 6.52) | 3.41 (1.23, 10.04) | 1.33 (0.69, 2.61) |
| 1.04 (0.36, 2.94) | 0.98 (0.43, 2.22) | 3rd | 1.33 (0.69, 2.59) | 1.9 (1.01, 3.6) | 1.9 (0.97, 3.76) | 2.35 (1.16, 4.82) | 2.38 (1.15, 5.02) | 1.93 (0.9, 4.14) | 2.59 (1.16, 5.98) | 2.44 (1.07, 5.7) | 3.34 (1.32, 8.98) | 1.3 (0.79, 2.15) |
| 0.78 (0.28, 2.13) | 0.74 (0.33, 1.62) | 0.75 (0.39, 1.46) | 4th | 1.43 (0.79, 2.57) | 1.43 (0.76, 2.7) | 1.77 (0.91, 3.5) | 1.79 (0.9, 3.64) | 1.45 (0.7, 3.01) | 1.95 (0.9, 4.36) | 1.84 (0.83, 4.2) | 2.51 (1.01, 6.57) | 0.98 (0.63, 1.52) |
| 0.55 (0.2, 1.49) | 0.52 (0.24, 1.11) | 0.53 (0.28, 1) | 0.7 (0.39, 1.27) | 5th | 1 (0.55, 1.84) | 1.24 (0.65, 2.39) | 1.26 (0.64, 2.48) | 1.02 (0.5, 2.06) | 1.37 (0.65, 2.96) | 1.29 (0.59, 2.87) | 1.76 (0.72, 4.52) | 0.69 (0.46, 1.02) |
| 0.55 (0.19, 1.52) | 0.51 (0.23, 1.14) | 0.53 (0.27, 1.03) | 0.7 (0.37, 1.31) | 1 (0.54, 1.82) | 6th | 1.23 (0.63, 2.47) | 1.25 (0.62, 2.56) | 1.01 (0.49, 2.11) | 1.36 (0.62, 3.03) | 1.28 (0.57, 2.94) | 1.75 (0.71, 4.62) | 0.68 (0.43, 1.08) |
| 0.44 (0.15, 1.26) | 0.42 (0.18, 0.95) | 0.42 (0.21, 0.87) | 0.56 (0.29, 1.1) | 0.8 (0.42, 1.53) | 0.81 (0.41, 1.59) | 7th | 1.01 (0.48, 2.14) | 0.82 (0.38, 1.77) | 1.1 (0.48, 2.54) | 1.04 (0.45, 2.46) | 1.42 (0.55, 3.82) | 0.55 (0.33, 0.92) |
| 0.44 (0.15, 1.26) | 0.41 (0.17, 0.96) | 0.42 (0.2, 0.87) | 0.56 (0.27, 1.12) | 0.8 (0.4, 1.55) | 0.8 (0.39, 1.62) | 0.99 (0.47, 2.08) | 8th | 0.81 (0.37, 1.78) | 1.09 (0.47, 2.55) | 1.02 (0.43, 2.48) | 1.4 (0.53, 3.84) | 0.55 (0.31, 0.94) |
| 0.54 (0.18, 1.59) | 0.51 (0.21, 1.21) | 0.52 (0.24, 1.11) | 0.69 (0.33, 1.42) | 0.98 (0.49, 1.98) | 0.99 (0.47, 2.06) | 1.22 (0.56, 2.65) | 1.24 (0.56, 2.73) | 9th | 1.35 (0.56, 3.25) | 1.27 (0.52, 3.12) | 1.73 (0.65, 4.86) | 0.68 (0.38, 1.21) |
| 0.4 (0.13, 1.22) | 0.38 (0.15, 0.95) | 0.39 (0.17, 0.86) | 0.51 (0.23, 1.11) | 0.73 (0.34, 1.55) | 0.74 (0.33, 1.62) | 0.91 (0.39, 2.07) | 0.92 (0.39, 2.14) | 0.74 (0.31, 1.77) | 10th | 0.94 (0.37, 2.41) | 1.29 (0.46, 3.75) | 0.5 (0.26, 0.95) |
| 0.43 (0.13, 1.33) | 0.4 (0.15, 1.03) | 0.41 (0.18, 0.94) | 0.54 (0.24, 1.21) | 0.78 (0.35, 1.68) | 0.78 (0.34, 1.75) | 0.97 (0.41, 2.24) | 0.98 (0.4, 2.32) | 0.79 (0.32, 1.91) | 1.06 (0.41, 2.71) | 11th | 1.37 (0.48, 4.03) | 0.53 (0.27, 1.04) |
| 0.31 (0.09, 1.05) | 0.29 (0.1, 0.82) | 0.3 (0.11, 0.76) | 0.4 (0.15, 0.99) | 0.57 (0.22, 1.38) | 0.57 (0.22, 1.42) | 0.71 (0.26, 1.82) | 0.71 (0.26, 1.87) | 0.58 (0.21, 1.53) | 0.78 (0.27, 2.19) | 0.73 (0.25, 2.1) | 12th | 0.39 (0.17, 0.86) |
| 0.8 (0.31, 2) | 0.75 (0.38, 1.45) | 0.77 (0.47, 1.26) | 1.02 (0.66, 1.59) | 1.45 (0.98, 2.17) | 1.46 (0.93, 2.31) | 1.81 (1.09, 3.03) | 1.83 (1.07, 3.2) | 1.48 (0.83, 2.65) | 1.99 (1.05, 3.88) | 1.87 (0.96, 3.75) | 2.55 (1.16, 6.02) | chemo |

Matrix of pairwise comparisons of Dur + Tre + chemo (shown as odds ratios and 95% confidence intervals).

| 1st | 1.11 (0.39, 3.12) | 1.04 (0.38, 2.81) | 0.89 (0.33, 2.39) | 0.86 (0.32, 2.27) | 0.9 (0.34, 2.38) | 1.07 (0.4, 2.85) | 1.34 (0.49, 3.63) | 1.65 (0.6, 4.59) | 1.71 (0.61, 4.84) | 2.43 (0.84, 7) | 3.87 (1.28, 11.97) | 1 (0.4, 2.49) |
| --- | --- | --- | --- | --- | --- | --- | --- | --- | --- | --- | --- | --- |
| 0.9 (0.32, 2.59) | 2nd | 0.94 (0.49, 1.82) | 0.8 (0.42, 1.52) | 0.78 (0.42, 1.42) | 0.81 (0.44, 1.49) | 0.96 (0.51, 1.82) | 1.21 (0.63, 2.32) | 1.49 (0.76, 2.93) | 1.55 (0.76, 3.13) | 2.19 (1.05, 4.63) | 3.49 (1.58, 8.01) | 0.91 (0.54, 1.51) |
| 0.96 (0.36, 2.63) | 1.06 (0.55, 2.03) | 3rd | 0.85 (0.49, 1.49) | 0.82 (0.48, 1.4) | 0.86 (0.51, 1.46) | 1.02 (0.59, 1.79) | 1.28 (0.72, 2.29) | 1.58 (0.87, 2.92) | 1.64 (0.87, 3.1) | 2.32 (1.19, 4.64) | 3.71 (1.78, 8.05) | 0.96 (0.64, 1.45) |
| 1.12 (0.42, 3.07) | 1.24 (0.66, 2.37) | 1.17 (0.67, 2.06) | 4th | 0.96 (0.58, 1.62) | 1.01 (0.6, 1.68) | 1.2 (0.7, 2.07) | 1.51 (0.86, 2.65) | 1.86 (1.03, 3.38) | 1.93 (1.04, 3.58) | 2.72 (1.41, 5.38) | 4.35 (2.12, 9.34) | 1.13 (0.77, 1.67) |
| 1.17 (0.44, 3.1) | 1.29 (0.7, 2.38) | 1.22 (0.72, 2.07) | 1.04 (0.62, 1.72) | 5th | 1.04 (0.65, 1.69) | 1.24 (0.74, 2.07) | 1.56 (0.92, 2.65) | 1.92 (1.1, 3.4) | 2 (1.11, 3.62) | 2.82 (1.5, 5.4) | 4.5 (2.25, 9.42) | 1.17 (0.83, 1.64) |
| 1.12 (0.42, 2.98) | 1.24 (0.67, 2.28) | 1.17 (0.68, 1.98) | 0.99 (0.6, 1.66) | 0.96 (0.59, 1.55) | 6th | 1.19 (0.72, 1.98) | 1.5 (0.88, 2.55) | 1.84 (1.05, 3.27) | 1.91 (1.06, 3.46) | 2.7 (1.44, 5.19) | 4.31 (2.15, 9.13) | 1.12 (0.8, 1.58) |
| 0.94 (0.35, 2.53) | 1.04 (0.55, 1.95) | 0.98 (0.56, 1.7) | 0.83 (0.48, 1.43) | 0.8 (0.48, 1.34) | 0.84 (0.51, 1.4) | 7th | 1.26 (0.72, 2.2) | 1.55 (0.85, 2.81) | 1.6 (0.87, 2.97) | 2.26 (1.18, 4.44) | 3.61 (1.77, 7.8) | 0.94 (0.64, 1.37) |
| 0.74 (0.28, 2.05) | 0.83 (0.43, 1.6) | 0.78 (0.44, 1.38) | 0.66 (0.38, 1.16) | 0.64 (0.38, 1.08) | 0.67 (0.39, 1.13) | 0.8 (0.45, 1.39) | 8th | 1.23 (0.67, 2.27) | 1.28 (0.68, 2.42) | 1.81 (0.92, 3.62) | 2.88 (1.38, 6.24) | 0.75 (0.5, 1.12) |
| 0.6 (0.22, 1.67) | 0.67 (0.34, 1.32) | 0.63 (0.34, 1.15) | 0.54 (0.3, 0.97) | 0.52 (0.29, 0.91) | 0.54 (0.31, 0.95) | 0.65 (0.36, 1.17) | 0.81 (0.44, 1.49) | 9th | 1.04 (0.53, 2.03) | 1.47 (0.73, 3.01) | 2.34 (1.09, 5.18) | 0.61 (0.38, 0.95) |
| 0.59 (0.21, 1.65) | 0.65 (0.32, 1.31) | 0.61 (0.32, 1.15) | 0.52 (0.28, 0.96) | 0.5 (0.28, 0.9) | 0.52 (0.29, 0.94) | 0.62 (0.34, 1.15) | 0.78 (0.41, 1.47) | 0.97 (0.49, 1.88) | 10th | 1.41 (0.68, 2.96) | 2.26 (1.03, 5.14) | 0.59 (0.36, 0.95) |
| 0.41 (0.14, 1.19) | 0.46 (0.22, 0.96) | 0.43 (0.22, 0.84) | 0.37 (0.19, 0.71) | 0.35 (0.19, 0.67) | 0.37 (0.19, 0.7) | 0.44 (0.23, 0.84) | 0.55 (0.28, 1.09) | 0.68 (0.33, 1.37) | 0.71 (0.34, 1.46) | 11th | 1.6 (0.7, 3.73) | 0.42 (0.24, 0.71) |
| 0.26 (0.08, 0.78) | 0.29 (0.12, 0.63) | 0.27 (0.12, 0.56) | 0.23 (0.11, 0.47) | 0.22 (0.11, 0.44) | 0.23 (0.11, 0.47) | 0.28 (0.13, 0.57) | 0.35 (0.16, 0.72) | 0.43 (0.19, 0.91) | 0.44 (0.19, 0.97) | 0.63 (0.27, 1.43) | 12th | 0.26 (0.13, 0.48) |
| 1 (0.4, 2.5) | 1.1 (0.66, 1.84) | 1.04 (0.69, 1.57) | 0.89 (0.6, 1.3) | 0.86 (0.61, 1.2) | 0.89 (0.63, 1.25) | 1.06 (0.73, 1.55) | 1.33 (0.89, 2.01) | 1.64 (1.05, 2.6) | 1.71 (1.05, 2.79) | 2.41 (1.42, 4.21) | 3.84 (2.09, 7.48) | chemo |

Matrix of pairwise comparisons of Dur + chemo (shown as odds ratios and 95% confidence intervals).

| 1st | 1.61 (0.56, 4.71) | 1.83 (0.67, 5.18) | 1.43 (0.53, 3.99) | 1.15 (0.43, 3.12) | 1.22 (0.46, 3.31) | 1.46 (0.54, 4) | 2.07 (0.76, 5.8) | 2.02 (0.73, 5.75) | 2.24 (0.8, 6.48) | 3.18 (1.1, 9.43) | 5.06 (1.68, 15.94) | 1.25 (0.5, 3.2) |
| --- | --- | --- | --- | --- | --- | --- | --- | --- | --- | --- | --- | --- |
| 0.62 (0.21, 1.79) | 2nd | 1.15 (0.57, 2.28) | 0.89 (0.46, 1.74) | 0.72 (0.38, 1.35) | 0.76 (0.4, 1.43) | 0.91 (0.47, 1.73) | 1.29 (0.66, 2.51) | 1.26 (0.63, 2.54) | 1.4 (0.68, 2.87) | 1.98 (0.93, 4.25) | 3.14 (1.4, 7.34) | 0.78 (0.45, 1.32) |
| 0.55 (0.19, 1.49) | 0.87 (0.44, 1.75) | 3rd | 0.78 (0.43, 1.41) | 0.63 (0.36, 1.09) | 0.66 (0.38, 1.15) | 0.79 (0.44, 1.4) | 1.12 (0.62, 2.04) | 1.1 (0.59, 2.06) | 1.22 (0.64, 2.36) | 1.72 (0.87, 3.47) | 2.75 (1.29, 6.05) | 0.68 (0.44, 1.05) |
| 0.7 (0.25, 1.9) | 1.12 (0.58, 2.18) | 1.28 (0.71, 2.31) | 4th | 0.8 (0.47, 1.36) | 0.85 (0.5, 1.44) | 1.01 (0.59, 1.76) | 1.44 (0.82, 2.55) | 1.41 (0.78, 2.57) | 1.56 (0.84, 2.92) | 2.21 (1.14, 4.36) | 3.52 (1.71, 7.59) | 0.87 (0.58, 1.3) |
| 0.87 (0.32, 2.31) | 1.39 (0.74, 2.64) | 1.59 (0.92, 2.78) | 1.25 (0.73, 2.11) | 5th | 1.06 (0.65, 1.71) | 1.26 (0.76, 2.1) | 1.79 (1.06, 3.06) | 1.76 (1.01, 3.08) | 1.94 (1.09, 3.52) | 2.75 (1.47, 5.25) | 4.39 (2.19, 9.22) | 1.08 (0.77, 1.53) |
| 0.82 (0.3, 2.18) | 1.32 (0.7, 2.48) | 1.51 (0.87, 2.63) | 1.18 (0.69, 1.99) | 0.94 (0.58, 1.53) | 6th | 1.19 (0.72, 1.98) | 1.69 (1, 2.88) | 1.66 (0.95, 2.91) | 1.84 (1.03, 3.33) | 2.6 (1.39, 4.99) | 4.13 (2.06, 8.75) | 1.02 (0.73, 1.44) |
| 0.69 (0.25, 1.84) | 1.1 (0.58, 2.12) | 1.27 (0.71, 2.25) | 0.99 (0.57, 1.7) | 0.79 (0.48, 1.31) | 0.84 (0.51, 1.4) | 7th | 1.42 (0.82, 2.48) | 1.39 (0.78, 2.5) | 1.54 (0.84, 2.86) | 2.18 (1.15, 4.23) | 3.47 (1.7, 7.39) | 0.86 (0.59, 1.25) |
| 0.48 (0.17, 1.31) | 0.78 (0.4, 1.51) | 0.89 (0.49, 1.61) | 0.69 (0.39, 1.22) | 0.56 (0.33, 0.94) | 0.59 (0.35, 1) | 0.7 (0.4, 1.22) | 8th | 0.98 (0.54, 1.79) | 1.08 (0.58, 2.03) | 1.54 (0.79, 3.02) | 2.44 (1.18, 5.32) | 0.61 (0.4, 0.9) |
| 0.49 (0.17, 1.37) | 0.79 (0.39, 1.58) | 0.91 (0.49, 1.69) | 0.71 (0.39, 1.29) | 0.57 (0.32, 0.99) | 0.6 (0.34, 1.05) | 0.72 (0.4, 1.29) | 1.02 (0.56, 1.87) | 9th | 1.1 (0.58, 2.13) | 1.56 (0.79, 3.15) | 2.49 (1.17, 5.53) | 0.62 (0.39, 0.96) |
| 0.45 (0.15, 1.25) | 0.72 (0.35, 1.46) | 0.82 (0.42, 1.57) | 0.64 (0.34, 1.19) | 0.51 (0.28, 0.92) | 0.54 (0.3, 0.97) | 0.65 (0.35, 1.19) | 0.92 (0.49, 1.71) | 0.9 (0.47, 1.74) | 10th | 1.42 (0.69, 2.91) | 2.25 (1.04, 5.05) | 0.56 (0.34, 0.89) |
| 0.31 (0.11, 0.91) | 0.51 (0.24, 1.08) | 0.58 (0.29, 1.15) | 0.45 (0.23, 0.87) | 0.36 (0.19, 0.68) | 0.39 (0.2, 0.72) | 0.46 (0.24, 0.87) | 0.65 (0.33, 1.27) | 0.64 (0.32, 1.27) | 0.71 (0.34, 1.44) | 11th | 1.59 (0.7, 3.71) | 0.39 (0.23, 0.67) |
| 0.2 (0.06, 0.6) | 0.32 (0.14, 0.72) | 0.36 (0.17, 0.77) | 0.28 (0.13, 0.59) | 0.23 (0.11, 0.46) | 0.24 (0.11, 0.49) | 0.29 (0.14, 0.59) | 0.41 (0.19, 0.85) | 0.4 (0.18, 0.85) | 0.44 (0.2, 0.96) | 0.63 (0.27, 1.42) | 12th | 0.25 (0.13, 0.45) |
| 0.8 (0.31, 2) | 1.29 (0.76, 2.2) | 1.47 (0.95, 2.29) | 1.15 (0.77, 1.71) | 0.92 (0.66, 1.29) | 0.98 (0.7, 1.37) | 1.16 (0.8, 1.7) | 1.65 (1.11, 2.49) | 1.62 (1.04, 2.54) | 1.79 (1.12, 2.91) | 2.54 (1.5, 4.39) | 4.02 (2.21, 7.83) | chemo |

Matrix of pairwise comparisons of Ipi + chemo (shown as odds ratios and 95% confidence intervals).

| 3rd | 1.25 (0.75, 2.1) | 1.27 (0.78, 2.08) | 1.28 (0.77, 2.12) | 1.17 (0.69, 1.98) | 1.26 (0.73, 2.17) | 1.61 (0.89, 2.91) | 1.78 (0.97, 3.32) | 1.53 (0.81, 2.9) | 1.46 (0.73, 2.95) | 1 (0.65, 1.51) |
| --- | --- | --- | --- | --- | --- | --- | --- | --- | --- | --- |
| 0.8 (0.48, 1.34) | 4th | 1.02 (0.68, 1.52) | 1.02 (0.67, 1.56) | 0.93 (0.6, 1.45) | 1 (0.63, 1.6) | 1.29 (0.77, 2.18) | 1.43 (0.83, 2.48) | 1.22 (0.7, 2.17) | 1.17 (0.62, 2.21) | 0.8 (0.58, 1.08) |
| 0.79 (0.48, 1.28) | 0.98 (0.66, 1.47) | 5th | 1 (0.68, 1.48) | 0.92 (0.61, 1.38) | 0.99 (0.64, 1.53) | 1.27 (0.78, 2.07) | 1.4 (0.84, 2.38) | 1.2 (0.7, 2.09) | 1.15 (0.62, 2.14) | 0.78 (0.6, 1.01) |
| 0.78 (0.47, 1.31) | 0.98 (0.64, 1.49) | 1 (0.67, 1.47) | 6th | 0.91 (0.59, 1.41) | 0.98 (0.63, 1.56) | 1.26 (0.76, 2.1) | 1.4 (0.82, 2.42) | 1.2 (0.69, 2.11) | 1.15 (0.62, 2.15) | 0.78 (0.59, 1.04) |
| 0.86 (0.51, 1.46) | 1.07 (0.69, 1.67) | 1.09 (0.72, 1.64) | 1.09 (0.71, 1.69) | 7th | 1.08 (0.67, 1.74) | 1.38 (0.82, 2.34) | 1.53 (0.89, 2.68) | 1.31 (0.74, 2.34) | 1.26 (0.66, 2.39) | 0.86 (0.62, 1.18) |
| 0.8 (0.46, 1.37) | 1 (0.63, 1.58) | 1.01 (0.65, 1.56) | 1.02 (0.64, 1.6) | 0.93 (0.58, 1.49) | 8th | 1.28 (0.74, 2.21) | 1.42 (0.8, 2.52) | 1.21 (0.68, 2.21) | 1.16 (0.61, 2.25) | 0.79 (0.56, 1.12) |
| 0.62 (0.34, 1.12) | 0.78 (0.46, 1.3) | 0.79 (0.48, 1.29) | 0.79 (0.48, 1.31) | 0.72 (0.43, 1.22) | 0.78 (0.45, 1.34) | 9th | 1.11 (0.6, 2.06) | 0.95 (0.5, 1.8) | 0.91 (0.45, 1.83) | 0.62 (0.4, 0.93) |
| 0.56 (0.3, 1.03) | 0.7 (0.4, 1.21) | 0.71 (0.42, 1.19) | 0.72 (0.41, 1.22) | 0.65 (0.37, 1.13) | 0.7 (0.4, 1.24) | 0.9 (0.48, 1.66) | 10th | 0.86 (0.44, 1.66) | 0.82 (0.4, 1.67) | 0.56 (0.35, 0.87) |
| 0.65 (0.35, 1.23) | 0.82 (0.46, 1.44) | 0.83 (0.48, 1.42) | 0.83 (0.47, 1.45) | 0.76 (0.43, 1.35) | 0.82 (0.45, 1.48) | 1.05 (0.55, 1.99) | 1.17 (0.6, 2.25) | 11th | 0.96 (0.46, 1.99) | 0.65 (0.4, 1.04) |
| 0.68 (0.34, 1.36) | 0.85 (0.45, 1.61) | 0.87 (0.47, 1.6) | 0.87 (0.47, 1.62) | 0.8 (0.42, 1.51) | 0.86 (0.44, 1.64) | 1.1 (0.55, 2.2) | 1.22 (0.6, 2.48) | 1.05 (0.5, 2.18) | 12th | 0.68 (0.39, 1.18) |
| 1 (0.66, 1.53) | 1.26 (0.93, 1.71) | 1.28 (0.99, 1.66) | 1.28 (0.96, 1.71) | 1.17 (0.85, 1.62) | 1.26 (0.89, 1.79) | 1.61 (1.07, 2.47) | 1.79 (1.15, 2.84) | 1.53 (0.96, 2.5) | 1.47 (0.85, 2.58) | chemo |

Matrix of pairwise comparisons of Pem + chemo (shown as odds ratios and 95% confidence intervals).

| 1st | 1.2 (0.36, 4.08) | 1.06 (0.34, 3.39) | 1.35 (0.44, 4.19) | 1.76 (0.59, 5.38) | 2.2 (0.72, 6.87) | 2.71 (0.88, 8.54) | 3.26 (1.03, 10.5) | 3.13 (0.96, 10.55) | 3.48 (1.03, 11.99) | 4.73 (1.36, 16.98) | 6.66 (1.81, 26.97) | 1.3 (0.46, 3.72) |
| --- | --- | --- | --- | --- | --- | --- | --- | --- | --- | --- | --- | --- |
| 0.84 (0.24, 2.79) | 2nd | 0.89 (0.4, 1.95) | 1.13 (0.54, 2.4) | 1.47 (0.72, 3.04) | 1.84 (0.87, 3.88) | 2.26 (1.06, 4.95) | 2.73 (1.23, 6.15) | 2.61 (1.11, 6.25) | 2.9 (1.21, 7.21) | 3.93 (1.56, 10.42) | 5.57 (2.01, 17) | 1.09 (0.59, 2.03) |
| 0.94 (0.3, 2.94) | 1.12 (0.51, 2.48) | 3rd | 1.27 (0.67, 2.42) | 1.66 (0.9, 3.07) | 2.07 (1.1, 3.93) | 2.55 (1.31, 5.06) | 3.07 (1.51, 6.36) | 2.94 (1.38, 6.5) | 3.27 (1.49, 7.47) | 4.43 (1.91, 10.85) | 6.26 (2.45, 17.97) | 1.22 (0.76, 2) |
| 0.74 (0.24, 2.26) | 0.89 (0.42, 1.86) | 0.78 (0.41, 1.49) | 4th | 1.3 (0.74, 2.29) | 1.62 (0.9, 2.94) | 2 (1.07, 3.77) | 2.4 (1.24, 4.76) | 2.31 (1.12, 4.86) | 2.56 (1.2, 5.64) | 3.47 (1.55, 8.26) | 4.89 (1.99, 13.68) | 0.96 (0.63, 1.46) |
| 0.57 (0.19, 1.7) | 0.68 (0.33, 1.38) | 0.6 (0.33, 1.11) | 0.77 (0.44, 1.35) | 5th | 1.25 (0.71, 2.19) | 1.54 (0.85, 2.81) | 1.85 (0.98, 3.56) | 1.77 (0.88, 3.66) | 1.97 (0.95, 4.25) | 2.67 (1.22, 6.2) | 3.77 (1.55, 10.38) | 0.74 (0.51, 1.07) |
| 0.45 (0.15, 1.39) | 0.54 (0.26, 1.14) | 0.48 (0.25, 0.91) | 0.62 (0.34, 1.11) | 0.8 (0.46, 1.41) | 6th | 1.23 (0.66, 2.31) | 1.48 (0.77, 2.93) | 1.42 (0.69, 2.98) | 1.58 (0.74, 3.45) | 2.14 (0.96, 5.05) | 3.02 (1.22, 8.31) | 0.59 (0.39, 0.89) |
| 0.37 (0.12, 1.14) | 0.44 (0.2, 0.95) | 0.39 (0.2, 0.76) | 0.5 (0.27, 0.93) | 0.65 (0.36, 1.18) | 0.81 (0.43, 1.51) | 7th | 1.2 (0.6, 2.43) | 1.15 (0.54, 2.5) | 1.28 (0.58, 2.87) | 1.73 (0.75, 4.2) | 2.46 (0.97, 6.96) | 0.48 (0.3, 0.76) |
| 0.31 (0.1, 0.97) | 0.37 (0.16, 0.81) | 0.33 (0.16, 0.66) | 0.42 (0.21, 0.81) | 0.54 (0.28, 1.02) | 0.68 (0.34, 1.3) | 0.83 (0.41, 1.67) | 8th | 0.96 (0.44, 2.13) | 1.06 (0.47, 2.46) | 1.44 (0.61, 3.58) | 2.04 (0.78, 5.9) | 0.4 (0.23, 0.66) |
| 0.32 (0.09, 1.05) | 0.38 (0.16, 0.9) | 0.34 (0.15, 0.73) | 0.43 (0.21, 0.89) | 0.56 (0.27, 1.13) | 0.71 (0.34, 1.44) | 0.87 (0.4, 1.85) | 1.04 (0.47, 2.29) | 9th | 1.11 (0.46, 2.69) | 1.51 (0.6, 3.9) | 2.12 (0.77, 6.4) | 0.42 (0.22, 0.75) |
| 0.29 (0.08, 0.97) | 0.34 (0.14, 0.82) | 0.31 (0.13, 0.67) | 0.39 (0.18, 0.83) | 0.51 (0.24, 1.05) | 0.63 (0.29, 1.35) | 0.78 (0.35, 1.72) | 0.94 (0.41, 2.13) | 0.9 (0.37, 2.16) | 10th | 1.36 (0.52, 3.62) | 1.91 (0.68, 5.86) | 0.38 (0.19, 0.7) |
| 0.21 (0.06, 0.73) | 0.25 (0.1, 0.64) | 0.23 (0.09, 0.52) | 0.29 (0.12, 0.65) | 0.37 (0.16, 0.82) | 0.47 (0.2, 1.04) | 0.58 (0.24, 1.33) | 0.69 (0.28, 1.65) | 0.66 (0.26, 1.67) | 0.74 (0.28, 1.92) | 11th | 1.42 (0.46, 4.5) | 0.28 (0.13, 0.55) |
| 0.15 (0.04, 0.55) | 0.18 (0.06, 0.5) | 0.16 (0.06, 0.41) | 0.2 (0.07, 0.5) | 0.27 (0.1, 0.64) | 0.33 (0.12, 0.82) | 0.41 (0.14, 1.03) | 0.49 (0.17, 1.28) | 0.47 (0.16, 1.29) | 0.52 (0.17, 1.47) | 0.71 (0.22, 2.16) | 12th | 0.2 (0.08, 0.44) |
| 0.77 (0.27, 2.16) | 0.92 (0.49, 1.7) | 0.82 (0.5, 1.32) | 1.04 (0.68, 1.58) | 1.35 (0.93, 1.97) | 1.69 (1.12, 2.56) | 2.08 (1.32, 3.35) | 2.5 (1.51, 4.27) | 2.4 (1.34, 4.45) | 2.66 (1.43, 5.2) | 3.61 (1.83, 7.74) | 5.08 (2.3, 12.98) | chemo |

Matrix of pairwise comparisons of Ser + chemo (shown as odds ratios and 95% confidence intervals).

| 1st | 1.23 (0.26, 6.17) | 1.35 (0.31, 6.31) | 1.28 (0.3, 5.84) | 1.45 (0.35, 6.44) | 1.65 (0.4, 7.3) | 1.74 (0.42, 7.83) | 1.66 (0.4, 7.49) | 1.49 (0.36, 6.67) | 1.44 (0.35, 6.53) | 1.51 (0.36, 6.77) | 1.34 (0.32, 6) | 0.63 (0.16, 2.73) |
| --- | --- | --- | --- | --- | --- | --- | --- | --- | --- | --- | --- | --- |
| 0.81 (0.16, 3.83) | 2nd | 1.1 (0.46, 2.63) | 1.04 (0.45, 2.42) | 1.18 (0.53, 2.61) | 1.34 (0.6, 2.98) | 1.41 (0.63, 3.15) | 1.35 (0.6, 3.03) | 1.21 (0.54, 2.73) | 1.18 (0.52, 2.63) | 1.22 (0.54, 2.76) | 1.08 (0.47, 2.47) | 0.52 (0.25, 1.05) |
| 0.74 (0.16, 3.18) | 0.91 (0.38, 2.18) | 3rd | 0.95 (0.48, 1.85) | 1.07 (0.58, 1.98) | 1.21 (0.66, 2.27) | 1.28 (0.69, 2.42) | 1.22 (0.66, 2.3) | 1.1 (0.59, 2.07) | 1.07 (0.57, 2.03) | 1.11 (0.59, 2.11) | 0.98 (0.52, 1.88) | 0.47 (0.28, 0.78) |
| 0.78 (0.17, 3.28) | 0.96 (0.41, 2.21) | 1.05 (0.54, 2.06) | 4th | 1.13 (0.64, 1.99) | 1.28 (0.73, 2.28) | 1.35 (0.76, 2.42) | 1.29 (0.72, 2.32) | 1.16 (0.65, 2.08) | 1.12 (0.63, 2.04) | 1.17 (0.65, 2.13) | 1.04 (0.57, 1.9) | 0.49 (0.32, 0.77) |
| 0.69 (0.16, 2.84) | 0.85 (0.38, 1.89) | 0.94 (0.51, 1.73) | 0.89 (0.5, 1.56) | 5th | 1.14 (0.69, 1.89) | 1.2 (0.72, 2.01) | 1.15 (0.68, 1.92) | 1.03 (0.61, 1.74) | 1 (0.59, 1.69) | 1.04 (0.61, 1.77) | 0.92 (0.54, 1.58) | 0.44 (0.31, 0.62) |
| 0.61 (0.14, 2.49) | 0.75 (0.34, 1.66) | 0.82 (0.44, 1.52) | 0.78 (0.44, 1.37) | 0.88 (0.53, 1.45) | 6th | 1.05 (0.63, 1.76) | 1.01 (0.6, 1.69) | 0.9 (0.54, 1.53) | 0.88 (0.52, 1.49) | 0.91 (0.54, 1.55) | 0.81 (0.47, 1.38) | 0.39 (0.27, 0.55) |
| 0.57 (0.13, 2.37) | 0.71 (0.32, 1.58) | 0.78 (0.41, 1.45) | 0.74 (0.41, 1.32) | 0.83 (0.5, 1.39) | 0.95 (0.57, 1.59) | 7th | 0.95 (0.56, 1.62) | 0.86 (0.5, 1.46) | 0.83 (0.49, 1.42) | 0.86 (0.5, 1.49) | 0.77 (0.44, 1.32) | 0.36 (0.25, 0.53) |
| 0.6 (0.13, 2.49) | 0.74 (0.33, 1.67) | 0.82 (0.44, 1.53) | 0.77 (0.43, 1.38) | 0.87 (0.52, 1.46) | 0.99 (0.59, 1.67) | 1.05 (0.62, 1.77) | 8th | 0.9 (0.53, 1.53) | 0.87 (0.51, 1.49) | 0.91 (0.53, 1.56) | 0.8 (0.47, 1.39) | 0.38 (0.26, 0.55) |
| 0.67 (0.15, 2.79) | 0.83 (0.37, 1.86) | 0.91 (0.48, 1.7) | 0.86 (0.48, 1.54) | 0.97 (0.58, 1.63) | 1.11 (0.66, 1.87) | 1.17 (0.68, 1.98) | 1.11 (0.65, 1.89) | 9th | 0.97 (0.56, 1.67) | 1.01 (0.58, 1.75) | 0.89 (0.51, 1.55) | 0.43 (0.29, 0.62) |
| 0.69 (0.15, 2.85) | 0.85 (0.38, 1.91) | 0.94 (0.49, 1.76) | 0.89 (0.49, 1.59) | 1 (0.59, 1.69) | 1.14 (0.67, 1.93) | 1.2 (0.71, 2.05) | 1.15 (0.67, 1.96) | 1.03 (0.6, 1.77) | 10th | 1.04 (0.6, 1.8) | 0.92 (0.53, 1.61) | 0.44 (0.3, 0.64) |
| 0.66 (0.15, 2.77) | 0.82 (0.36, 1.84) | 0.9 (0.47, 1.7) | 0.86 (0.47, 1.54) | 0.96 (0.57, 1.63) | 1.1 (0.64, 1.86) | 1.16 (0.67, 1.99) | 1.1 (0.64, 1.9) | 0.99 (0.57, 1.71) | 0.96 (0.56, 1.67) | 11th | 0.89 (0.51, 1.55) | 0.42 (0.28, 0.62) |
| 0.75 (0.17, 3.14) | 0.92 (0.41, 2.11) | 1.02 (0.53, 1.92) | 0.96 (0.53, 1.75) | 1.09 (0.63, 1.85) | 1.24 (0.72, 2.11) | 1.3 (0.76, 2.25) | 1.25 (0.72, 2.15) | 1.12 (0.64, 1.94) | 1.09 (0.62, 1.89) | 1.13 (0.65, 1.98) | 12th | 0.48 (0.32, 0.7) |
| 1.58 (0.37, 6.23) | 1.94 (0.95, 3.97) | 2.14 (1.29, 3.53) | 2.03 (1.29, 3.17) | 2.28 (1.6, 3.26) | 2.6 (1.82, 3.72) | 2.74 (1.9, 3.98) | 2.62 (1.8, 3.82) | 2.35 (1.62, 3.46) | 2.28 (1.56, 3.38) | 2.37 (1.61, 3.54) | 2.1 (1.42, 3.16) | chemo |

Matrix of pairwise comparisons of Ben + Anl + chemo (shown as odds ratios and 95% confidence intervals).

| 1st | 1.12(0.36, 3.41) | 1.11(0.38, 3.15) | 1.24(0.43, 3.43) | 2.89(1.02, **0**.96) | 4.42 (1.51, 12.58) | 4.78 (1.55, 14.48) | 5.54 (1.76, 17.42) | 10.9 (2.95, 45.33) | 13.1 (3, 76.19) | 20.57 (4.02, 174.28) | 32.66 (4.25, 988) | 0.71(0.27, 1.81) |
| --- | --- | --- | --- | --- | --- | --- | --- | --- | --- | --- | --- | --- |
| 0.89(0.29, 2.81) | 2nd | 0.99(0.46, 2.13) | 1.11 (0.53, 2.3) | 2.6 (1.26, 5.21) | 3.95 (1.85, 8.44) | 4.27 (1.86, 9.9) | 4.96 (2.11, 12.02) | 9.73 (3.36, 33.89) | 11.65 (3.33, 57.31) | 18.16 (4.36, 140.21) | 28.99 (4.43, 832.72) | 0.64(0.35, 1.15) |
| 0.9 (0.32, 2.65) | 1.01(0.47, 2.17) | 3rd | 1.12 (0.6, 2.11) | 2.61(1.42, 4.75) | 3.99 (2.07, 7.69) | 4.29 (2.07, 9.22) | 4.98 (2.32, 11.32) | 9.81 (3.6, 32.18) | 11.69 (3.61, 55.47) | 18.24 (4.65, 134.79) | 29.17 (4.67, 820.27) | 0.64 (0.4, 1.02) |
| 0.81(0.29, 2.32) | 0.9 (0.43, 1.89) | 0.9 (0.47, 1.68) | 4th | 2.33(1.32, 4.11) | 3.57 (1.91, 6.69) | 3.83 (1.9, 8.01) | 4.46 (2.14, 9.75) | 8.76 (3.3, 28.18) | 10.41 (3.28, 49.96) | 16.26 (4.23, 120.21) | 25.81 (4.29, 720.95) | 0.57(0.37, 0.87) |
| 0.35(0.13, 0.98) | 0.38(0.19, 0.79) | 0.38(0.21, 0.7) | 0.43(0.24, 0.76) | 5th | 1.53 (0.85, 2.78) | 1.64 (0.84, 3.36) | 1.91 (0.94, 4.1) | 3.74 (1.44, 11.97) | 4.47 (1.43, 20.92) | 6.98 (1.84, 51.08) | 11.05 (1.86, 311.97) | 0.25(0.17, 0.36) |
| 0.23(0.08, 0.66) | 0.25(0.12, 0.54) | 0.25(0.13, 0.48) | 0.28(0.15, 0.52) | 0.65(0.36, 1.18) | 6th | 1.07 (0.52, 2.3) | 1.25 (0.58, 2.79) | 2.45 (0.91, 8.03) | 2.92 (0.91, 13.95) | 4.58 (1.16, 34.05) | 7.25 (1.18, 202.75) | 0.16 (0.1, 0.25) |
| 0.21(0.07, 0.65) | 0.23 (0.1, 0.54) | 0.23(0.11, 0.48) | 0.26(0.12, 0.53) | 0.61 (0.3, 1.19) | 0.93 (0.43, 1.92) | 7th | 1.16 (0.49, 2.79) | 2.29 (0.79, 7.72) | 2.72 (0.79, 13.33) | 4.25 (1.02, 32.15) | 6.75 (1.05, 191.39) | 0.15(0.08, 0.26) |
| 0.18(0.06, 0.57) | 0.2 (0.08, 0.47) | 0.2 (0.09, 0.43) | 0.22 (0.1, 0.47) | 0.52(0.24, 1.06) | 0.8 (0.36, 1.72) | 0.86 (0.36, 2.02) | 8th | 1.97 (0.66, 6.75) | 2.35 (0.65, 11.81) | 3.65 (0.86, 27.61) | 5.8 (0.89, 169.41) | 0.13(0.07, 0.23) |
| 0.09(0.02, 0.34) | 0.1 (0.03, 0.3) | 0.1 (0.03, 0.28) | 0.11 (0.04, 0.3) | 0.27(0.08, 0.7) | 0.41 (0.12, 1.1) | 0.44 (0.13, 1.27) | 0.51 (0.15, 1.52) | 9th | 1.19 (0.26, 6.78) | 1.86 (0.34, 15.27) | 2.96 (0.37, 88.94) | 0.07(0.02, 0.16) |
| 0.08(0.01, 0.33) | 0.09(0.02, 0.3) | 0.09(0.02, 0.28) | 0.1 (0.02, 0.3) | 0.22 (0.05, 0.7) | 0.34 (0.07, 1.1) | 0.37 (0.08, 1.27) | 0.43 (0.08, 1.54) | 0.84 (0.15, 3.87) | 10th | 1.56 (0.22, 14.39) | 2.48 (0.25, 78.09) | 0.06(0.01, 0.16) |
| 0.05 (0.01, 0.25) | 0.06 (0.01, 0.23) | 0.05 (0.01, 0.22) | 0.06 (0.01, 0.24) | 0.14(0.02, 0.54) | 0.22 (0.03, 0.86) | 0.24 (0.03, 0.98) | 0.27 (0.04, 1.16) | 0.54 (0.07, 2.92) | 0.64 (0.07, 4.54) | 11th | 1.59 (0.12, 51.88) | 0.04 (0, 0.13) |
| 0.03 (0, 0.24) | 0.03 (0, 0.23) | 0.03 (0, 0.21) | 0.04 (0, 0.23) | 0.09 (0, 0.54) | 0.14 (0, 0.85) | 0.15 (0.01, 0.95) | 0.17 (0.01, 1.13) | 0.34 (0.01, 2.68) | 0.4 (0.01, 4.08) | 0.63 (0.02, 8.63) | 12th | 0.02 (0, 0.13) |
| 1.41 (0.55, 3.74) | 1.57 (0.87, 2.9) | 1.56 (0.98, 2.51) | 1.74 (1.15, 2.68) | 4.08 (2.8, 5.95) | 6.22 (3.98, 10) | 6.68 (3.84, 12.33) | 7.78 (4.3, 15.12) | 15.2 (6.4, 45.68) | 18.12 (6.25, 82.3) | 28.34 (7.99, 200.28) | 44.9 (7.93, 1249) | chemo |

Matrix of pairwise comparisons of Tis + chemo (shown as odds ratios and 95% confidence intervals).

| 1st | 0.67 (0.1, 4.55) | 0.84(0.13,5.35) | 0.81(0.13,5.11) | 1.73(0.29,10.74) | 2.68(0.44,16.77) | 3.27(0.53,20.7) | 3.97(0.63,25.55) | 4.85(0.76,32.15) | 4.88(0.75,32.95) | 4.75(0.73,31.75) | 6.05(0.91,41.97) | 1.01(0.17,6.02) |
| --- | --- | --- | --- | --- | --- | --- | --- | --- | --- | --- | --- | --- |
| 1.49 (0.22, 10.08) | 2nd | 1.25(0.52,3.05) | 1.22(0.53,2.85) | 2.6 (1.18, 5.89) | 4 (1.74, 9.42) | 4.88(2.1,11.78) | 5.91(2.46,14.76) | 7.25(2.89,18.79) | 7.28(2.83,19.6) | 7.1 (2.75, 19.3) | 8.98(3.34,25.84) | 1.51 (0.75,3.14) |
| 1.19 (0.19, 7.46) | 0.8 (0.33, 1.92) | 3rd | 0.97 (0.5, 1.94) | 2.08 (1.1, 3.97) | 3.21 (1.63, 6.34) | 3.91(1.94,8.03) | 4.73(2.28,10.13) | 5.8 (2.62, 13.16) | 5.81 (2.59, 13.71) | 5.66 (2.51, 13.4) | 7.18 (3.01, 18.24) | 1.21 (0.72, 2.04) |
| 1.23 (0.2, 7.55) | 0.82 (0.35, 1.89) | 1.03 (0.52, 2.02) | 4th | 2.14 (1.2, 3.82) | 3.28 (1.78, 6.15) | 4.02(2.11,7.8) | 4.87 (2.48, 9.79) | 5.95 (2.86, 12.87) | 5.97 (2.81, 13.43) | 5.83 (2.72, 13.09) | 7.37 (3.28, 17.92) | 1.24 (0.8, 1.93) |
| 0.58 (0.09, 3.49) | 0.38 (0.17, 0.85) | 0.48 (0.25, 0.91) | 0.47 (0.26, 0.83) | 5th | 1.54 (0.87, 2.74) | 1.88(1.03,3.51) | 2.27 (1.2, 4.41) | 2.77 (1.38, 5.84) | 2.79 (1.35, 6.04) | 2.72 (1.31, 5.92) | 3.44 (1.59, 8.16) | 0.58 (0.4, 0.84) |
| 0.37 (0.06, 2.29) | 0.25 (0.11, 0.58) | 0.31 (0.16, 0.61) | 0.3 (0.16, 0.56) | 0.65 (0.36, 1.15) | 6th | 1.22 (0.64,2.3) | 1.48 (0.75, 2.96) | 1.81 (0.87, 3.88) | 1.81 (0.85, 4.06) | 1.77 (0.83, 3.96) | 2.24 (1, 5.43) | 0.38 (0.24, 0.58) |
| 0.31 (0.05, 1.9) | 0.2 (0.08, 0.48) | 0.26 (0.12, 0.52) | 0.25 (0.13, 0.47) | 0.53 (0.28, 0.97) | 0.82 (0.42, 1.57) | 7th | 1.21 (0.59, 2.5) | 1.48 (0.69, 3.28) | 1.49 (0.68, 3.39) | 1.45 (0.65, 3.29) | 1.83 (0.79, 4.6) | 0.31 (0.19, 0.5) |
| 0.25 (0.04, 1.58) | 0.17 (0.07, 0.41) | 0.21 (0.1, 0.44) | 0.21 (0.1, 0.4) | 0.44 (0.23, 0.83) | 0.68 (0.34, 1.34) | 0.82 (0.4, 1.7) | 8th | 1.22 (0.55, 2.78) | 1.23 (0.54, 2.87) | 1.2 (0.52, 2.82) | 1.51 (0.63, 3.84) | 0.26 (0.15, 0.43) |
| 0.21 (0.03, 1.32) | 0.14 (0.05, 0.35) | 0.17 (0.08, 0.38) | 0.17 (0.08, 0.35) | 0.36 (0.17, 0.72) | 0.55 (0.26, 1.15) | 0.68 (0.31,1.4) | 0.82 (0.36, 1.83) | 9th | 1.01 (0.41, 2.48) | 0.98 (0.4, 2.42) | 1.24 (0.49, 3.27) | 0.21 (0.11, 0.38) |
| 0.2 (0.03, 1.34) | 0.14 (0.05, 0.35) | 0.17 (0.07, 0.39) | 0.17 (0.07, 0.36) | 0.36 (0.17, 0.74) | 0.55 (0.25, 1.18) | 0.67 (0.3, 1.48) | 0.81 (0.35, 1.85) | 0.99 (0.4, 2.41) | 10th | 0.97 (0.39, 2.44) | 1.23 (0.47, 3.29) | 0.21 (0.1, 0.39) |
| 0.21 (0.03, 1.37) | 0.14 (0.05, 0.36) | 0.18 (0.07, 0.4) | 0.17 (0.08, 0.37) | 0.37 (0.17, 0.76) | 0.57 (0.25, 1.21) | 0.69 (0.3, 1.54) | 0.84 (0.35, 1.92) | 1.02 (0.41, 2.48) | 1.03 (0.41, 2.58) | 11th | 1.27 (0.48, 3.41) | 0.21 (0.11, 0.4) |
| 0.17 (0.02, 1.09) | 0.11 (0.04, 0.3) | 0.14 (0.05, 0.33) | 0.14 (0.06, 0.31) | 0.29 (0.12, 0.63) | 0.45 (0.18, 1) | 0.55 (0.22,1.27) | 0.66 (0.26, 1.58) | 0.81 (0.31, 2.06) | 0.81 (0.3, 2.14) | 0.79 (0.29, 2.1) | 12th | 0.17 (0.08, 0.33) |
| 0.99 (0.17, 5.83) | 0.66 (0.32, 1.34) | 0.83 (0.49, 1.39) | 0.81 (0.52, 1.25) | 1.73 (1.19, 2.51) | 2.65 (1.72, 4.13) | 3.24 (2.02,5.3) | 3.92 (2.34, 6.79) | 4.78 (2.66, 9.1) | 4.8 (2.59, 9.54) | 4.67 (2.52, 9.34) | 5.91 (3.01, 12.93) | chemo |

Matrix of pairwise comparisons of Tor + chemo (shown as odds ratios and 95% confidence intervals).

| 1st | 0.2 (0.01, 2.15) | 0.32(0.01, 3.19) | 0.27(0.01, 2.69) | 0.31(0.01, 2.92) | 0.32(0.01, 3.06) | 0.43(0.01, 4.13) | 0.49(0.02, 4.83) | 0.81 (0.03, 8.08) | 0.84 (0.03, 8.49) | 0.83(0.03, 8.38) | 1.14 (0.04, 11.82) | 0.26(0.01, 2.34) |
| --- | --- | --- | --- | --- | --- | --- | --- | --- | --- | --- | --- | --- |
| 5.06 (0.47, 155.7) | 2nd | 1.61 (0.6, 4.44) | 1.35(0.51,3.6) | 1.54(0.62,3.9) | 1.61(0.65,4.0) | 2.14(0.86, 5.5) | 2.46(0.96,6.3) | 4.07 (1.53, 11.17) | 4.22 (1.54, 12.04) | 4.16(1.52, 11.9) | 5.7 (1.99, 17.14) | 1.27(0.56, 2.96) |
| 3.13 (0.31, 93.32) | 0.62(0.23, 1.68) | 3rd | 0.84(0.38, 1.82) | 0.96(0.48, 1.92) | 1 (0.5, 1.99) | 1.33(0.66, 2.7) | 1.53(0.74, 3.15) | 2.54 (1.15, 5.55) | 2.63 (1.16, 6.02) | 2.59(1.13, 6.07) | 3.55 (1.49, 8.83) | 0.79 (0.44, 1.4) |
| 3.72 (0.37, 111.47) | 0.74(0.28, 1.95) | 1.19(0.55, 2.6) | 4th | 1.14(0.59, 2.21) | 1.19(0.62, 2.3) | 1.59(0.81, 3.1) | 1.83(0.92, 3.65) | 3.02 (1.43, 6.43) | 3.13 (1.43, 7.04) | 3.08 (1.4, 7.03) | 4.23 (1.83, 10.22) | 0.95(0.56, 1.6) |
| 3.24 (0.34, 95) | 0.65(0.26, 1.61) | 1.04(0.52, 2.09) | 0.87(0.45, 1.69) | 5th | 1.04 (0.6, 1.82) | 1.39(0.78, 2.47) | 1.6 (0.88, 2.92) | 2.64 (1.36, 5.19) | 2.73 (1.35, 5.74) | 2.7 (1.32, 5.71) | 3.69 (1.73, 8.35) | 0.83(0.56, 1.23) |
| 3.11 (0.33, 89.91) | 0.62(0.25, 1.54) | 1 (0.5, 1.99) | 0.84(0.43, 1.61) | 0.96(0.55, 1.66) | 6th | 1.33(0.76, 2.34) | 1.53(0.85, 2.77) | 2.52 (1.31, 4.93) | 2.62 (1.31, 5.39) | 2.58 (1.27, 5.4) | 3.53 (1.67, 7.92) | 0.79(0.54, 1.16) |
| 2.34 (0.24, 68.5) | 0.47(0.18, 1.17) | 0.75(0.37, 1.52) | 0.63(0.32, 1.23) | 0.72 (0.4, 1.28) | 0.75(0.43, 1.32) | 7th | 1.15(0.63, 2.12) | 1.9 (0.97, 3.78) | 1.97 (0.97, 4.12) | 1.94(0.94, 4.14) | 2.66 (1.24, 6.08) | 0.6 (0.39, 0.89) |
| 2.04 (0.21, 59.21) | 0.41(0.16, 1.04) | 0.65(0.32, 1.36) | 0.55(0.27, 1.09) | 0.63(0.34, 1.14) | 0.66(0.36, 1.18) | 0.87(0.47, 1.59) | 8th | 1.65 (0.82, 3.35) | 1.71 (0.82, 3.69) | 1.69 (0.8, 3.67) | 2.31 (1.05, 5.36) | 0.52 (0.33, 0.8) |
| 1.24 (0.12, 36.94) | 0.25(0.09, 0.66) | 0.39(0.18, 0.87) | 0.33(0.16, 0.7) | 0.38(0.19, 0.74) | 0.4 (0.2, 0.76) | 0.53(0.26, 1.03) | 0.61 (0.3, 1.22) | 9th | 1.03 (0.46, 2.34) | 1.03(0.45, 2.34) | 1.4 (0.6, 3.39) | 0.31(0.18, 0.53) |
| 1.19 (0.12, 35.43) | 0.24(0.08, 0.65) | 0.38(0.17, 0.86) | 0.32(0.14, 0.7) | 0.37(0.17, 0.74) | 0.38(0.19, 0.77) | 0.51(0.24, 1.03) | 0.58(0.27, 1.21) | 0.97 (0.43, 2.15) | 10th | 0.98(0.42, 2.34) | 1.35 (0.55, 3.39) | 0.3 (0.16, 0.54) |
| 1.21 (0.12, 36.34) | 0.24(0.08, 0.66) | 0.39(0.16, 0.88) | 0.32(0.14, 0.72) | 0.37(0.18, 0.76) | 0.39(0.19, 0.79) | 0.52(0.24, 1.07) | 0.59(0.27, 1.25) | 0.98 (0.43, 2.2) | 1.02 (0.43, 2.39) | 11th | 1.37 (0.55, 3.47) | 0.31(0.16, 0.55) |
| 0.88 (0.08, 27.2) | 0.18 (0.06, 0.5) | 0.28(0.11, 0.67) | 0.24 (0.1, 0.55) | 0.27(0.12, 0.58) | 0.28(0.13, 0.6) | 0.38(0.16, 0.81) | 0.43(0.19, 0.95) | 0.71 (0.29, 1.67) | 0.74 (0.3, 1.81) | 0.73(0.29, 1.8) | 12th | 0.22(0.11, 0.42) |
| 3.92 (0.43, 113.15) | 0.79(0.34, 1.79) | 1.26(0.72, 2.25) | 1.06(0.63, 1.79) | 1.21(0.81, 1.79) | 1.26 (0.86, 1.86) | 1.68 (1.12, 2.55) | 1.93 (1.24, 3.04) | 3.19 (1.88, 5.57) | 3.3 (1.86, 6.13) | 3.26 (1.81, 6.17) | 4.45 (2.36, 9.14) | chemo |

Matrix of pairwise comparisons of Anl + chemo (shown as odds ratios and 95% confidence intervals).

| 1st | 2.28 (0.41, 13.02) | 1.77 (0.35, 8.82) | 1.71 (0.35, 8.36) | 3.64 (0.78, 17.06) | 5.01 (1.04, 23.61) | 4.69 (0.97, 22.59) | 5.48 (1.11, 26.72) | 6.45 (1.26, 33.75) | 5.42 (1.02, 29.71) | 7.95 (1.4, 47.48) | 6.24 (1.08, 37.67) | 1.01 (0.23, 4.53) |
| --- | --- | --- | --- | --- | --- | --- | --- | --- | --- | --- | --- | --- |
| 0.44 (0.08, 2.45) | 2nd | 0.78 (0.26, 2.21) | 0.75 (0.26, 2.02) | 1.61 (0.59, 4.07) | 2.2 (0.8, 5.66) | 2.07 (0.74, 5.42) | 2.4 (0.83, 6.59) | 2.85 (0.93, 8.6) | 2.38 (0.74, 7.64) | 3.48 (1, 12.72) | 2.74 (0.78, 10.09) | 0.45 (0.18, 1.03) |
| 0.57 (0.11, 2.83) | 1.29 (0.45, 3.82) | 3rd | 0.97 (0.44, 2.08) | 2.07 (1.03, 4.11) | 2.83 (1.36, 5.83) | 2.65 (1.25, 5.59) | 3.08 (1.4, 6.89) | 3.66 (1.51, 9.28) | 3.05 (1.19, 8.37) | 4.46 (1.6, 14.1) | 3.5 (1.25, 11.23) | 0.57 (0.31, 1.02) |
| 0.58 (0.12, 2.85) | 1.33 (0.5, 3.79) | 1.03 (0.48, 2.26) | 4th | 2.13 (1.14, 3.99) | 2.92 (1.51, 5.62) | 2.74 (1.38, 5.42) | 3.18 (1.54, 6.68) | 3.76 (1.66, 9.09) | 3.15 (1.3, 8.19) | 4.59 (1.74, 13.98) | 3.62 (1.34, 11.2) | 0.59 (0.36, 0.98) |
| 0.27 (0.06, 1.28) | 0.62 (0.25, 1.68) | 0.48 (0.24, 0.98) | 0.47 (0.25, 0.88) | 5th | 1.37 (0.78, 2.41) | 1.28 (0.71, 2.34) | 1.49 (0.79, 2.88) | 1.77 (0.83, 3.97) | 1.47 (0.66, 3.64) | 2.14 (0.87, 6.17) | 1.69 (0.67, 4.98) | 0.28 (0.19, 0.4) |
| 0.2 (0.04, 0.96) | 0.45 (0.18, 1.26) | 0.35 (0.17, 0.73) | 0.34 (0.18, 0.66) | 0.73 (0.41, 1.28) | 6th | 0.94 (0.5, 1.75) | 1.09 (0.56, 2.17) | 1.29 (0.6, 2.98) | 1.08 (0.47, 2.7) | 1.57 (0.62, 4.64) | 1.24 (0.48, 3.66) | 0.2 (0.13, 0.31) |
| 0.21 (0.04, 1.03) | 0.48 (0.18, 1.36) | 0.38 (0.18, 0.8) | 0.37 (0.18, 0.72) | 0.78 (0.43, 1.4) | 1.06 (0.57, 1.99) | 7th | 1.16 (0.58, 2.36) | 1.37 (0.62, 3.23) | 1.15 (0.49, 2.93) | 1.67 (0.64, 5.03) | 1.32 (0.5, 4.01) | 0.22 (0.14, 0.34) |
| 0.18 (0.04, 0.9) | 0.42 (0.15, 1.2) | 0.32 (0.15, 0.71) | 0.31 (0.15, 0.65) | 0.67 (0.35, 1.27) | 0.92 (0.46, 1.79) | 0.86 (0.42, 1.72) | 8th | 1.18 (0.51, 2.87) | 0.99 (0.4, 2.59) | 1.44 (0.53, 4.45) | 1.13 (0.41, 3.51) | 0.19 (0.11, 0.31) |
| 0.16 (0.03, 0.8) | 0.35 (0.12, 1.08) | 0.27 (0.11, 0.66) | 0.27 (0.11, 0.6) | 0.57 (0.25, 1.2) | 0.78 (0.34, 1.67) | 0.73 (0.31, 1.61) | 0.85 (0.35, 1.97) | 9th | 0.84 (0.3, 2.36) | 1.22 (0.41, 3.98) | 0.96 (0.31, 3.21) | 0.16 (0.08, 0.3) |
| 0.18 (0.03, 0.98) | 0.42 (0.13, 1.36) | 0.33 (0.12, 0.84) | 0.32 (0.12, 0.77) | 0.68 (0.27, 1.52) | 0.93 (0.37, 2.14) | 0.87 (0.34, 2.05) | 1.01 (0.39, 2.5) | 1.19 (0.42, 3.33) | 10th | 1.46 (0.45, 4.98) | 1.15 (0.35, 3.96) | 0.19 (0.08, 0.38) |
| 0.13 (0.02, 0.71) | 0.29 (0.08, 1) | 0.22 (0.07, 0.62) | 0.22 (0.07, 0.58) | 0.47 (0.16, 1.15) | 0.64 (0.22, 1.61) | 0.6 (0.2, 1.55) | 0.69 (0.22, 1.88) | 0.82 (0.25, 2.46) | 0.69 (0.2, 2.21) | 11th | 0.79 (0.21, 2.9) | 0.13 (0.05, 0.29) |
| 0.16 (0.03, 0.92) | 0.37 (0.1, 1.28) | 0.29 (0.09, 0.8) | 0.28 (0.09, 0.75) | 0.59 (0.2, 1.49) | 0.81 (0.27, 2.09) | 0.76 (0.25, 2.01) | 0.88 (0.28, 2.42) | 1.05 (0.31, 3.19) | 0.87 (0.25, 2.82) | 1.27 (0.34, 4.69) | 12th | 0.16 (0.06, 0.38) |
| 0.99 (0.22, 4.44) | 2.24 (0.97, 5.64) | 1.74 (0.98, 3.18) | 1.69 (1.03, 2.8) | 3.61 (2.5, 5.26) | 4.93 (3.26, 7.59) | 4.63 (2.95, 7.4) | 5.37 (3.26, 9.34) | 6.35 (3.34, 13.25) | 5.31 (2.61, 12.12) | 7.73 (3.42, 21.09) | 6.08 (2.63, 16.86) | chemo |

Matrix of pairwise comparisons of Bev + chemo (shown as odds ratios and 95% confidence intervals).

| 1st | 1.09 (0.24, 5) | 1.17 (0.27, 4.89) | 1.21 (0.28, 4.94) | 1.2 (0.3, 4.75) | 1.32 (0.32, 5.09) | 1.38 (0.34, 5.44) | 1.35 (0.32, 5.45) | 1.4 (0.33, 5.73) | 1.29 (0.29, 5.46) | 1.25 (0.29, 5.48) | 1.41 (0.31, 6.22) | 0.85 (0.23, 2.96) |
| --- | --- | --- | --- | --- | --- | --- | --- | --- | --- | --- | --- | --- |
| 0.92 (0.2, 4.25) | 2nd | 1.08 (0.36, 3.19) | 1.11 (0.38, 3.24) | 1.11 (0.4, 3.07) | 1.21 (0.43, 3.3) | 1.27 (0.45, 3.48) | 1.24 (0.43, 3.54) | 1.28 (0.43, 3.77) | 1.19 (0.39, 3.61) | 1.15 (0.37, 3.59) | 1.29 (0.4, 4.17) | 0.78 (0.32, 1.8) |
| 0.85 (0.2, 3.67) | 0.93 (0.31, 2.79) | 3rd | 1.03 (0.4, 2.66) | 1.03 (0.42, 2.5) | 1.11 (0.47, 2.67) | 1.17 (0.48, 2.84) | 1.15 (0.46, 2.87) | 1.19 (0.45, 3.1) | 1.1 (0.41, 2.95) | 1.07 (0.39, 2.97) | 1.2 (0.42, 3.46) | 0.72 (0.36, 1.41) |
| 0.83 (0.2, 3.53) | 0.9 (0.31, 2.66) | 0.97 (0.38, 2.5) | 4th | 1 (0.42, 2.37) | 1.09 (0.46, 2.54) | 1.14 (0.47, 2.72) | 1.12 (0.46, 2.75) | 1.16 (0.45, 2.96) | 1.07 (0.4, 2.85) | 1.04 (0.38, 2.84) | 1.16 (0.42, 3.33) | 0.7 (0.36, 1.33) |
| 0.83 (0.21, 3.38) | 0.9 (0.33, 2.53) | 0.97 (0.4, 2.36) | 1 (0.42, 2.39) | 5th | 1.08 (0.49, 2.42) | 1.14 (0.51, 2.57) | 1.12 (0.48, 2.6) | 1.15 (0.48, 2.83) | 1.07 (0.42, 2.71) | 1.04 (0.4, 2.72) | 1.16 (0.44, 3.18) | 0.7 (0.39, 1.24) |
| 0.76 (0.2, 3.09) | 0.83 (0.3, 2.31) | 0.9 (0.37, 2.14) | 0.92 (0.39, 2.18) | 0.92 (0.41, 2.05) | 6th | 1.05 (0.47, 2.35) | 1.03 (0.45, 2.36) | 1.07 (0.44, 2.57) | 0.98 (0.4, 2.47) | 0.96 (0.38, 2.47) | 1.07 (0.41, 2.9) | 0.65 (0.37, 1.12) |
| 0.73 (0.18, 2.96) | 0.79 (0.29, 2.22) | 0.85 (0.35, 2.07) | 0.88 (0.37, 2.11) | 0.88 (0.39, 1.98) | 0.95 (0.43, 2.13) | 7th | 0.98 (0.42, 2.25) | 1.01 (0.42, 2.46) | 0.94 (0.37, 2.36) | 0.91 (0.36, 2.38) | 1.02 (0.38, 2.76) | 0.62 (0.35, 1.08) |
| 0.74 (0.18, 3.1) | 0.81 (0.28, 2.34) | 0.87 (0.35, 2.17) | 0.9 (0.36, 2.19) | 0.89 (0.38, 2.07) | 0.97 (0.42, 2.21) | 1.02 (0.44, 2.36) | 8th | 1.03 (0.41, 2.6) | 0.96 (0.37, 2.48) | 0.93 (0.35, 2.5) | 1.04 (0.38, 2.9) | 0.63 (0.34, 1.15) |
| 0.72 (0.17, 3.06) | 0.78 (0.26, 2.34) | 0.84 (0.32, 2.21) | 0.86 (0.34, 2.22) | 0.87 (0.35, 2.09) | 0.94 (0.39, 2.25) | 0.99 (0.41, 2.38) | 0.97 (0.38, 2.43) | 9th | 0.92 (0.34, 2.51) | 0.9 (0.32, 2.51) | 1.01 (0.35, 2.94) | 0.61 (0.31, 1.19) |
| 0.77 (0.18, 3.41) | 0.84 (0.28, 2.59) | 0.91 (0.34, 2.42) | 0.93 (0.35, 2.49) | 0.94 (0.37, 2.35) | 1.02 (0.4, 2.52) | 1.07 (0.42, 2.67) | 1.05 (0.4, 2.69) | 1.08 (0.4, 2.9) | 10th | 0.97 (0.34, 2.78) | 1.09 (0.37, 3.2) | 0.66 (0.31, 1.33) |
| 0.8 (0.18, 3.48) | 0.87 (0.28, 2.72) | 0.93 (0.34, 2.59) | 0.96 (0.35, 2.6) | 0.96 (0.37, 2.48) | 1.04 (0.4, 2.65) | 1.1 (0.42, 2.8) | 1.08 (0.4, 2.84) | 1.11 (0.4, 3.09) | 1.03 (0.36, 2.92) | 11th | 1.12 (0.37, 3.4) | 0.67 (0.31, 1.43) |
| 0.71 (0.16, 3.22) | 0.77 (0.24, 2.49) | 0.83 (0.29, 2.37) | 0.86 (0.3, 2.38) | 0.86 (0.31, 2.29) | 0.93 (0.34, 2.45) | 0.98 (0.36, 2.6) | 0.96 (0.35, 2.6) | 0.99 (0.34, 2.82) | 0.91 (0.31, 2.71) | 0.89 (0.29, 2.68) | 12th | 0.6 (0.26, 1.32) |
| 1.18 (0.34, 4.27) | 1.28 (0.55, 3.08) | 1.39 (0.71, 2.76) | 1.43 (0.75, 2.77) | 1.43 (0.81, 2.54) | 1.55 (0.89, 2.71) | 1.62 (0.92, 2.89) | 1.59 (0.87, 2.96) | 1.64 (0.84, 3.27) | 1.53 (0.75, 3.18) | 1.48 (0.7, 3.22) | 1.66 (0.75, 3.8) | chemo |

Matrix of pairwise comparisons of Soc + chemo (shown as odds ratios and 95% confidence intervals).

| 1st | 0.52 (0.02, 5.3) | 0.53 (0.02, 4.92) | 0.41 (0.01, 3.54) | 0.38 (0.01, 3.19) | 0.49 (0.02, 4.11) | 0.52 (0.02, 4.36) | 0.47 (0.02, 3.99) | 0.45 (0.02, 3.95) | 0.72 (0.02, 6.35) | 0.8 (0.03, 7.16) | 0.8 (0.03, 7.21) | 0.19 (0.01, 1.53) |
| --- | --- | --- | --- | --- | --- | --- | --- | --- | --- | --- | --- | --- |
| 1.92 (0.19, 57.92) | 2nd | 1.01 (0.3, 3.21) | 0.78 (0.26, 2.15) | 0.73 (0.26, 1.85) | 0.93 (0.33, 2.4) | 0.99 (0.34, 2.59) | 0.89 (0.31, 2.39) | 0.87 (0.29, 2.37) | 1.37 (0.44, 4.02) | 1.52 (0.48, 4.58) | 1.52 (0.48, 4.55) | 0.36 (0.14, 0.86) |
| 1.88 (0.2, 54.59) | 0.99 (0.31, 3.3) | 3rd | 0.77 (0.31, 1.85) | 0.72 (0.31, 1.6) | 0.92 (0.39, 2.06) | 0.98 (0.4, 2.25) | 0.88 (0.36, 2.07) | 0.86 (0.34, 2.08) | 1.35 (0.51, 3.54) | 1.5 (0.56, 4) | 1.5 (0.56, 3.99) | 0.36 (0.16, 0.72) |
| 2.45 (0.28, 71.36) | 1.28 (0.47, 3.85) | 1.3 (0.54, 3.28) | 4th | 0.94 (0.49, 1.76) | 1.19 (0.61, 2.28) | 1.27 (0.64, 2.51) | 1.14 (0.57, 2.3) | 1.12 (0.53, 2.3) | 1.76 (0.79, 4.02) | 1.95 (0.86, 4.6) | 1.95 (0.85, 4.57) | 0.46 (0.27, 0.77) |
| 2.61 (0.31, 73.72) | 1.37 (0.54, 3.85) | 1.38 (0.62, 3.24) | 1.07 (0.57, 2.02) | 5th | 1.27 (0.75, 2.16) | 1.35 (0.77, 2.4) | 1.22 (0.68, 2.21) | 1.19 (0.64, 2.24) | 1.87 (0.94, 3.91) | 2.08 (1.01, 4.47) | 2.08 (1.01, 4.51) | 0.49 (0.35, 0.71) |
| 2.05 (0.24, 59.51) | 1.08 (0.42, 3.07) | 1.09 (0.49, 2.58) | 0.84 (0.44, 1.63) | 0.79 (0.46, 1.34) | 6th | 1.06 (0.59, 1.93) | 0.96 (0.52, 1.77) | 0.93 (0.49, 1.79) | 1.47 (0.72, 3.13) | 1.63 (0.78, 3.58) | 1.64 (0.79, 3.59) | 0.39 (0.26, 0.57) |
| 1.94 (0.23, 55.62) | 1.01 (0.39, 2.95) | 1.02 (0.45, 2.48) | 0.79 (0.4, 1.57) | 0.74 (0.42, 1.3) | 0.94 (0.52, 1.69) | 7th | 0.9 (0.47, 1.72) | 0.88 (0.45, 1.73) | 1.39 (0.66, 3.02) | 1.54 (0.72, 3.47) | 1.54 (0.71, 3.47) | 0.37 (0.23, 0.57) |
| 2.14 (0.25, 61.33) | 1.12 (0.42, 3.28) | 1.13 (0.48, 2.79) | 0.88 (0.43, 1.76) | 0.82 (0.45, 1.47) | 1.04 (0.57, 1.91) | 1.11 (0.58, 2.11) | 8th | 0.98 (0.49, 1.96) | 1.54 (0.71, 3.4) | 1.7 (0.78, 3.86) | 1.71 (0.78, 3.88) | 0.41 (0.25, 0.64) |
| 2.2 (0.25, 64.34) | 1.15 (0.42, 3.46) | 1.17 (0.48, 2.92) | 0.9 (0.43, 1.87) | 0.84 (0.45, 1.56) | 1.07 (0.56, 2.03) | 1.14 (0.58, 2.24) | 1.02 (0.51, 2.06) | 9th | 1.58 (0.72, 3.6) | 1.75 (0.78, 4.05) | 1.75 (0.77, 4.08) | 0.42 (0.25, 0.69) |
| 1.38 (0.16, 40.09) | 0.73 (0.25, 2.25) | 0.74 (0.28, 1.95) | 0.57 (0.25, 1.26) | 0.53 (0.26, 1.07) | 0.68 (0.32, 1.38) | 0.72 (0.33, 1.51) | 0.65 (0.29, 1.4) | 0.63 (0.28, 1.39) | 10th | 1.11 (0.45, 2.74) | 1.11 (0.45, 2.74) | 0.26 (0.14, 0.48) |
| 1.25 (0.14, 37.26) | 0.66 (0.22, 2.09) | 0.67 (0.25, 1.79) | 0.51 (0.22, 1.17) | 0.48 (0.22, 0.99) | 0.61 (0.28, 1.28) | 0.65 (0.29, 1.4) | 0.59 (0.26, 1.28) | 0.57 (0.25, 1.28) | 0.9 (0.36, 2.2) | 11th | 1 (0.4, 2.52) | 0.24 (0.12, 0.44) |
| 1.25 (0.14, 37.06) | 0.66 (0.22, 2.09) | 0.67 (0.25, 1.79) | 0.51 (0.22, 1.17) | 0.48 (0.22, 0.99) | 0.61 (0.28, 1.27) | 0.65 (0.29, 1.41) | 0.59 (0.26, 1.28) | 0.57 (0.24, 1.29) | 0.9 (0.37, 2.21) | 1 (0.4, 2.5) | 12th | 0.24 (0.12, 0.44) |
| 5.26 (0.65, 148.52) | 2.76 (1.17, 7.33) | 2.8 (1.39, 6.06) | 2.15 (1.29, 3.67) | 2.02 (1.42, 2.89) | 2.57 (1.74, 3.8) | 2.73 (1.77, 4.28) | 2.47 (1.56, 3.96) | 2.4 (1.45, 4.05) | 3.79 (2.1, 7.24) | 4.19 (2.26, 8.29) | 4.2 (2.26, 8.34) | chemo |

| Table S8 Incidence of Grade≥3 Adverse Events in Each Immunotherapy Combination (%). | | | | | | | | | | | | | | | |
| --- | --- | --- | --- | --- | --- | --- | --- | --- | --- | --- | --- | --- | --- | --- | --- |
|  | Ade + chemo | Ate + chemo | Dur + Tre + chemo | Dur + chemo | Ipi + chemo | Pem + chemo | Ser + chemo | Ben + Anl + chemo | Tis + chemo | Tor + chemo | Tir + Ate + chemo | Anl + chemo | Bev + Ate + chemo | Bev + chemo | Soc + chemo |
| Neutropenia | 76 | 23.2 | 32 | 24 | 14 | 43.5 | 4.4 | 69.5 | 55.9 | NR | 1.3 | 73.0 | NR | 46.3 | NR |
| Leukopenia | 46 | 5.1 | 6 | 6 | 1 | 11.7 | 2.6 | 38.2 | 0.4 | NR | NR | 30.7 | NR | 14.7 | NR |
| Thrombocytopenia | 38 | 10.1 | 9 | 6 | 4 | 13.9 | 6.2 | 49.6 | 19.3 | NR | 1.3 | 53.7 | NR | 4.2 | NR |
| Anemia | 27 | 14.1 | 13 | 9 | 8 | 15.7 | 5.4 | 24 | 16.2 | NR | 3.8 | 26.6 | NR | 3.2 | NR |
| Diarrhea | NR | 2 | 3 | 2 | 7 | 2.7 | NR | 0.8 | NR | NR | NR | 1.2 | NR | NR | NR |
| Vomiting | 1 | 1 | 2 | 0 | 1 | 0.9 | NR | 0.4 | 0.4 | NR | NR | 0.8 | NR | 3.2 | NR |
| Decreased appetite | 2 | 1 | 2 | 1 | 2 | 0.4 | NR | NR | 0.4 | NR | NR | NR | NR | NR | NR |
| Nausea | 1 | 0.5 | 2 | ＜1 | 1 | 0.9 | 0.3 | 0.4 | 0.4 | NR | NR | 0.8 | NR | 1.1 | NR |
| Fatigue | 18 | 1.5 | 1 | 2 | 2 | 2.7 | NR | 2.4 | 1.3 | NR | NR | 1.6 | NR | 8.4 | NR |
| Rash | NR | NR | 1 | 0 | 2 | 1.3 | NR | NR | 2.2 | NR | NR | NR | NR | NR | NR |
| Pruritus | NR | NR | 0 | 0 | 1 | 0 | NR | NR | NR | NR | NR | NR | NR | NR | NR |
| Alopecia | 0 | 0 | ＜1 | 1 | 0 | 0 | NR | 0 | 0 | NR | NR | 0 | NR | NR | NR |
| Constipation | 0 | 0.5 | ＜1 | 1 | NR | 0.4 | NR | 0 | 0 | NR | NR | 0 | NR | NR | NR |
| hypothyroidism | 0 | NR | NR | NR | NR | 0 | 0.3 | 0.4 | 0.4 | NR | NR | 0 | NR | NR | NR |
| hyperthyroidism | NR | NR | ＜1 | 0 | NR | NR | 0 | 0 | NR | NR | NR | 0 | NR | HR | NR |
| pneumonitis | 1 | NR | 5 | 2 | NR | 6.7 | NR | NR | NR | NR | 2.9 | NR | NR | NR | NR |
| hypertension | 3 | NR | 2 | 3 | NR | NR | NR | 15.5 | NR | NR | NR | 11.9 | NR | 6.3 | NR |
| Ipi + chemo, ipilimumab + chemotherapy; Pem + chemo, pembrolizumab + chemotherapy; Dur + Tre + chemo, durvalumab + tremelimuamb + chemotherapy; Dur + chemo, durvalumab + chemotherapy; Ade + chemo, adebrelimab + chemotherapy; Ate + chemo, atezolizumab + chemotherapy; Ser + chemo, serplulimab + chemotherapy; Tir + Ate + chemo, tiragolumab + atezolizumab + chemotherapy; Ben + Anl + chemo, benmelstobart + anlotinib + chemotherapy; Anl + chemo, anlotinib + chemotherapy; Tis + chemo, tislelizumab + chemotherapy; Tor + chemo, toripalimab + chemotherapy; Bev + Ate + chemo, bevacizumab + atezolizumab + chemotherapy; Bev + chemo, bevacizumab + chemotherapy; Soc + chemo, socazolimab + chemotherapy; | | | | | | | | | | | | | | | |

Figure S1 Results of risk of bias assessment.

1.
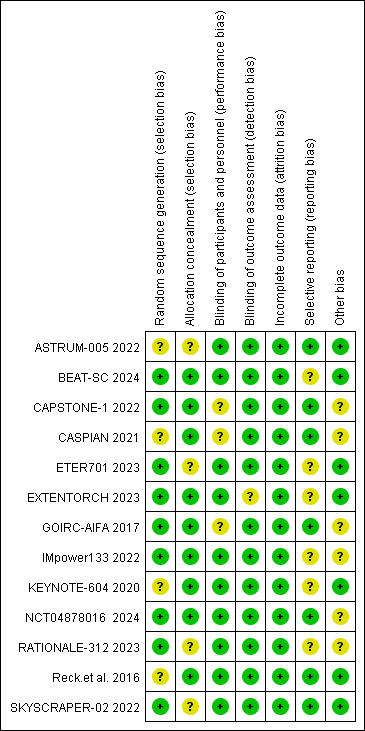
 （B）
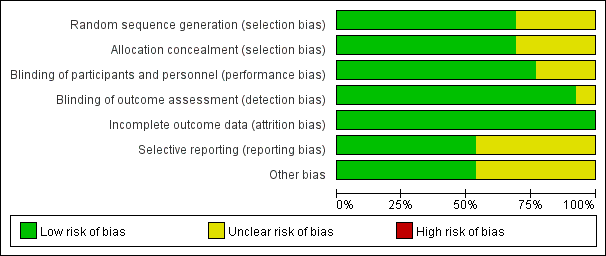


The studies’ risk of bias assessment: (A)Risk of bias summary (B) Risk of bias graph

Figure S2 The Brooks-Gelman-Rubin diagnostic and the density trace plot.

1. OS


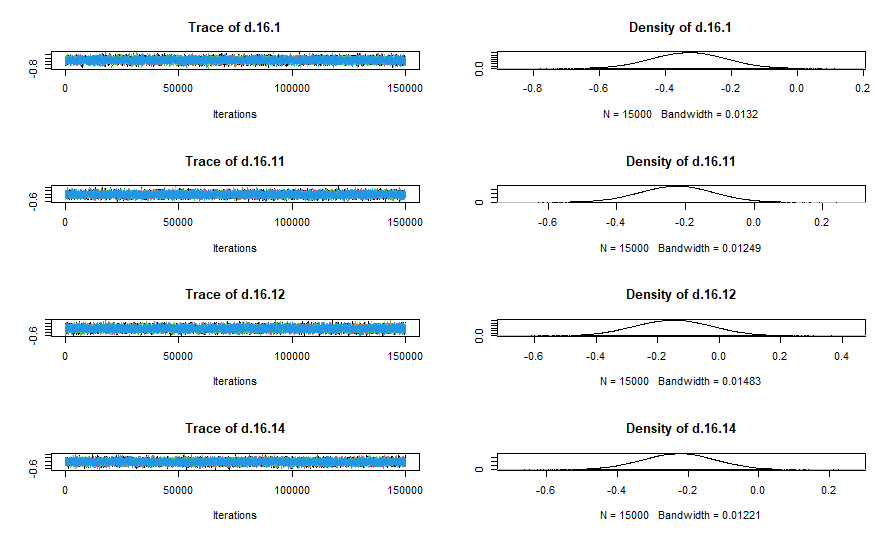

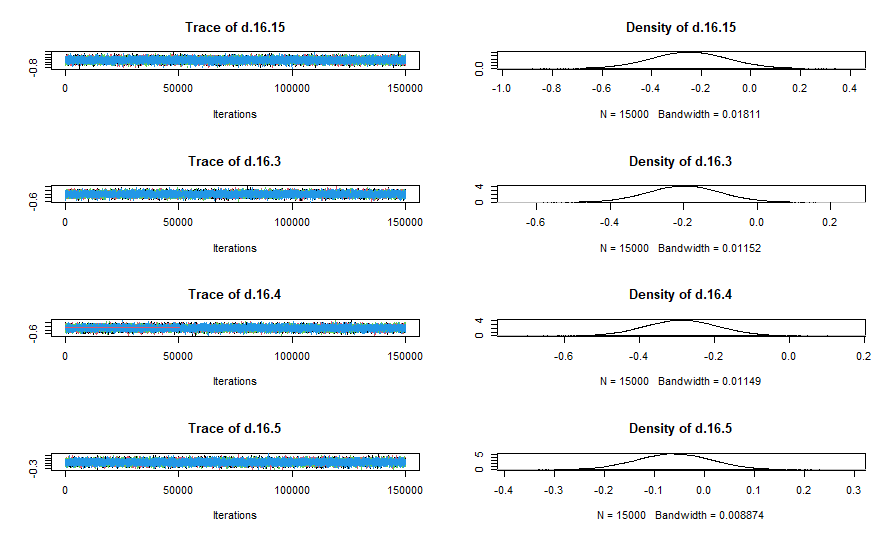


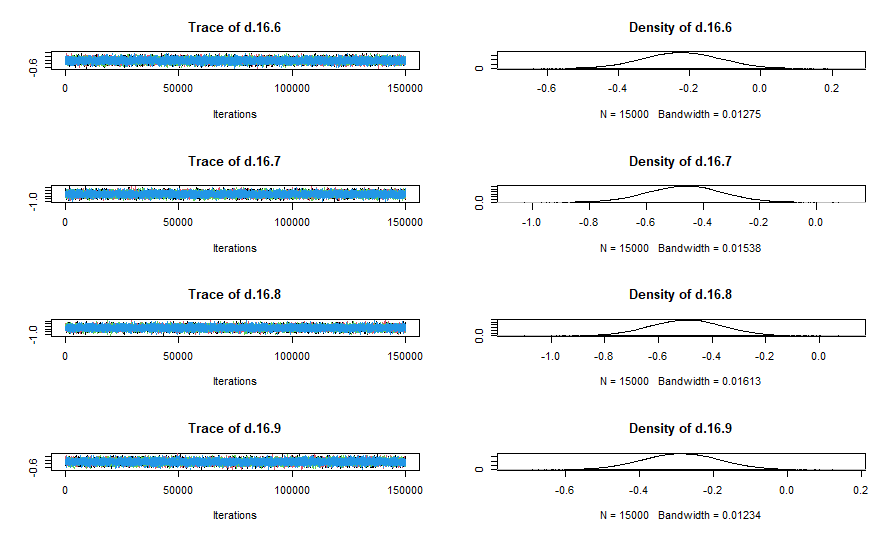

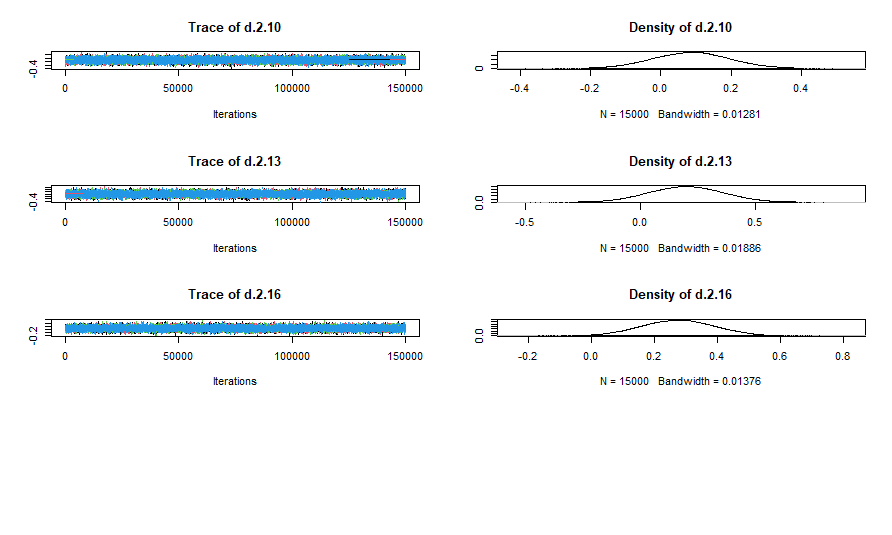


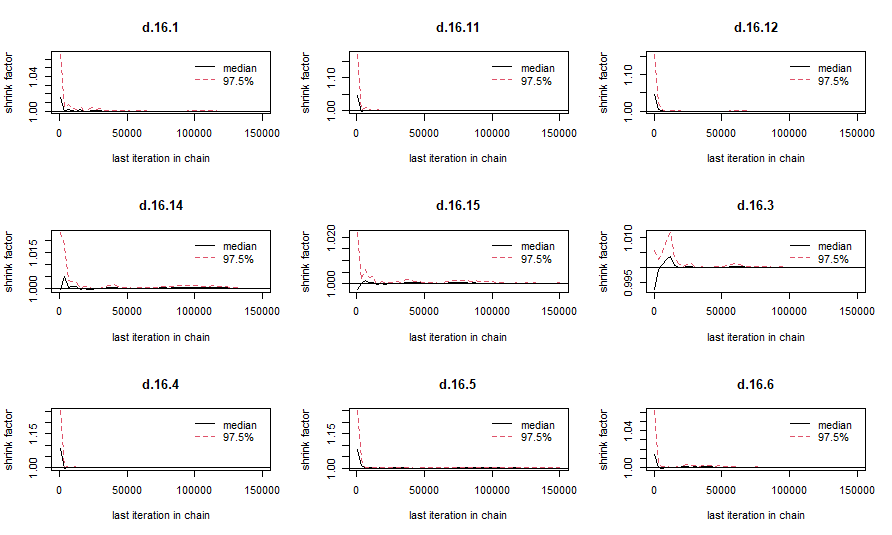

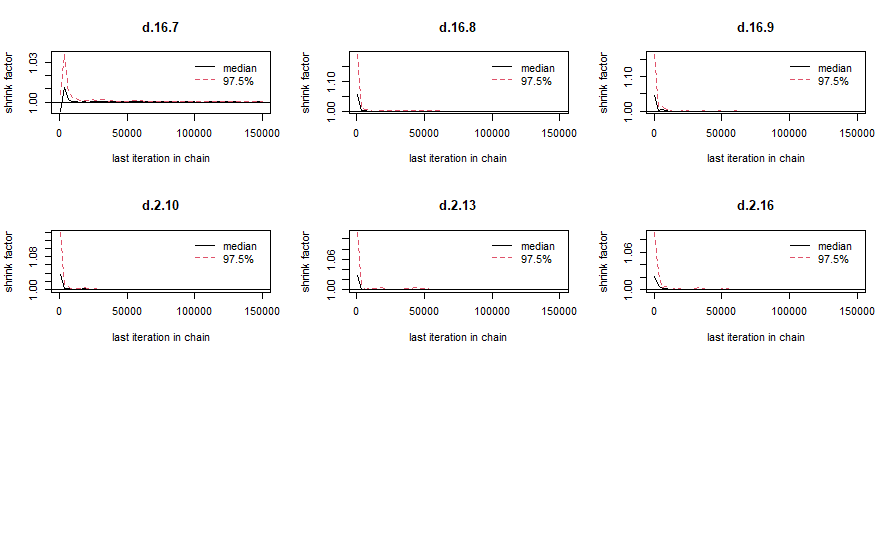


1. PFS


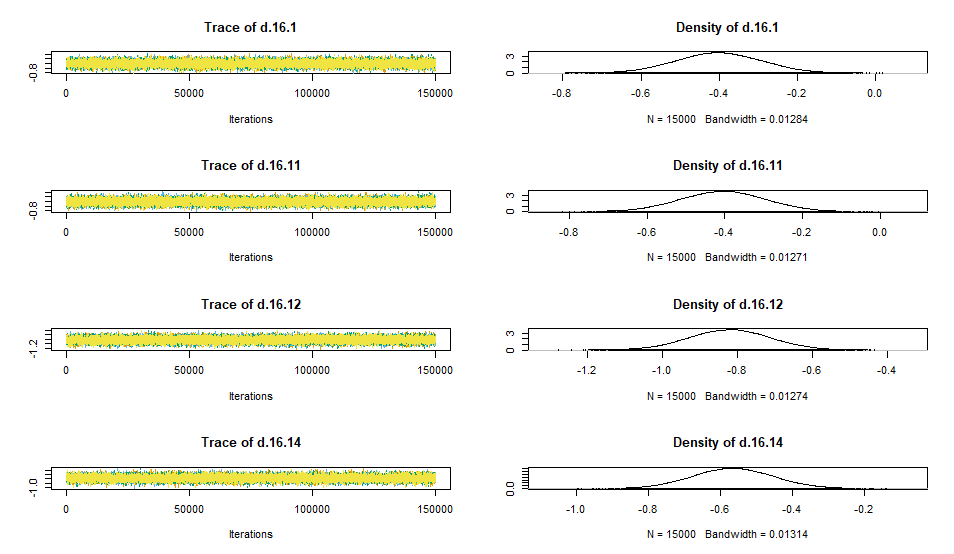

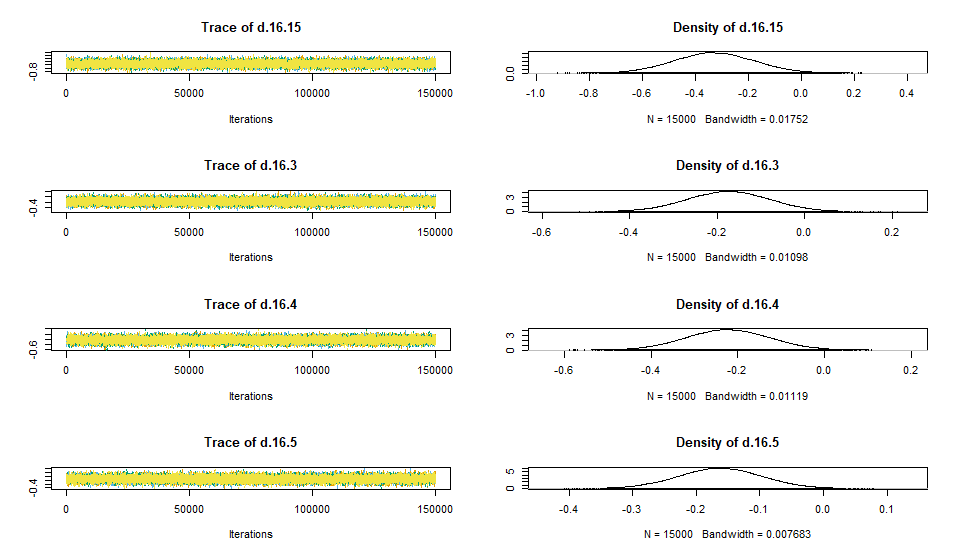


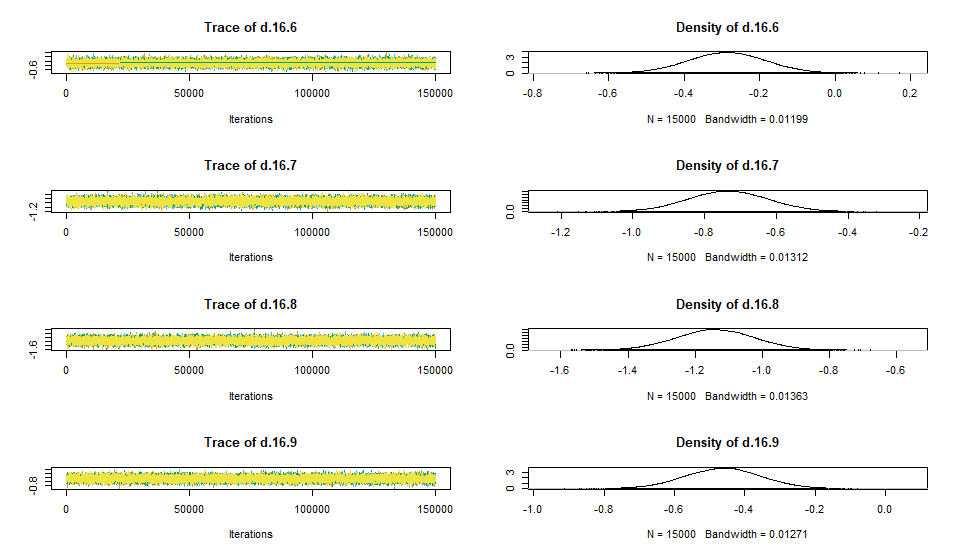

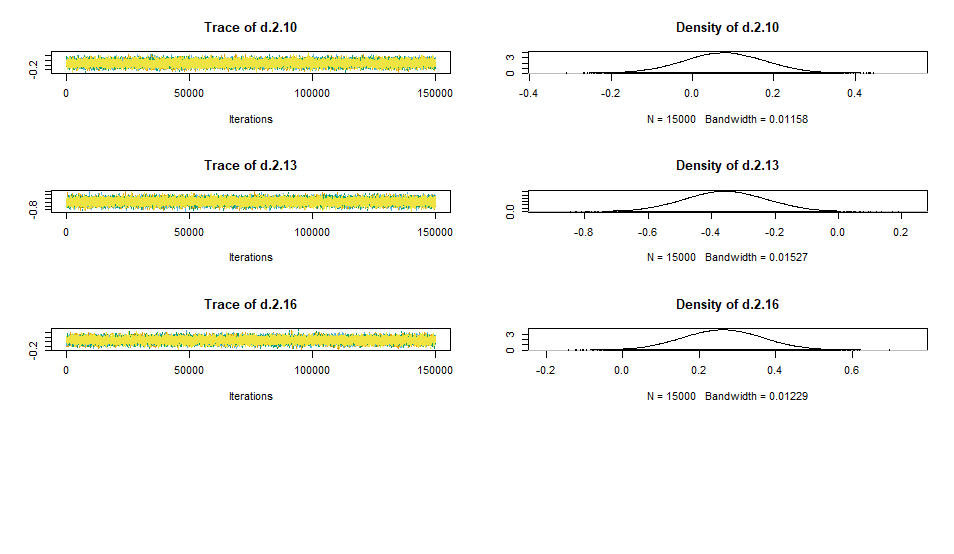


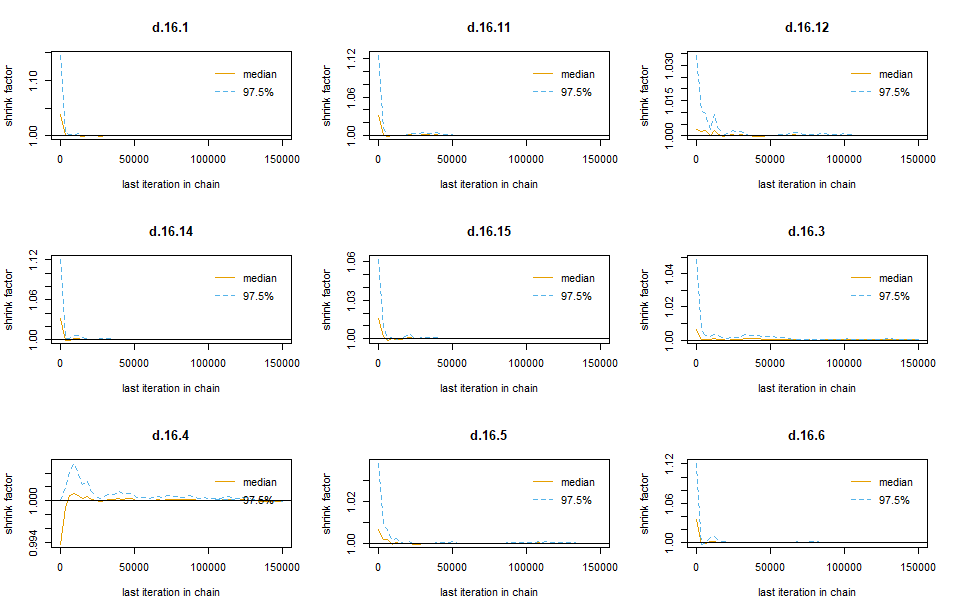

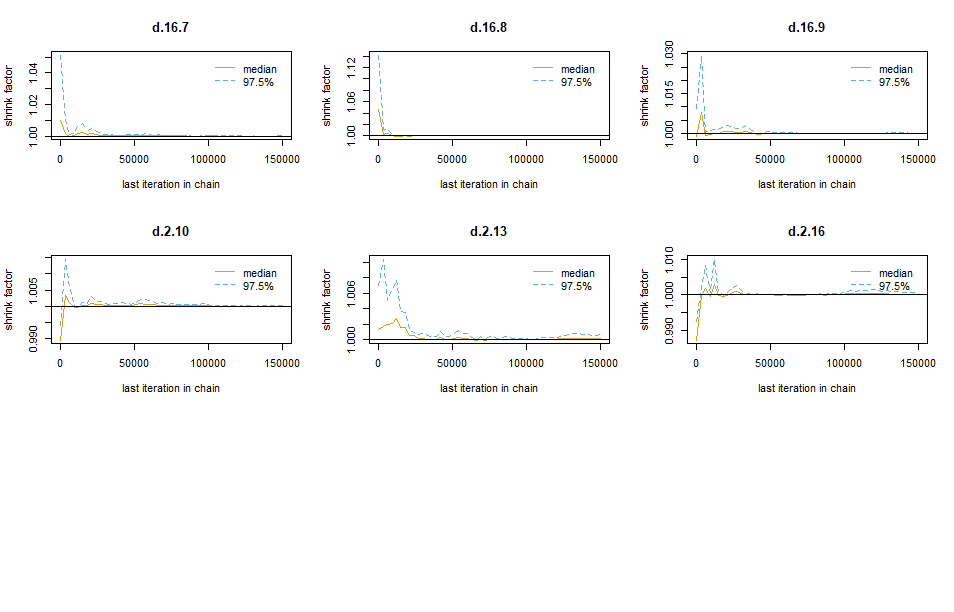


1. ORR


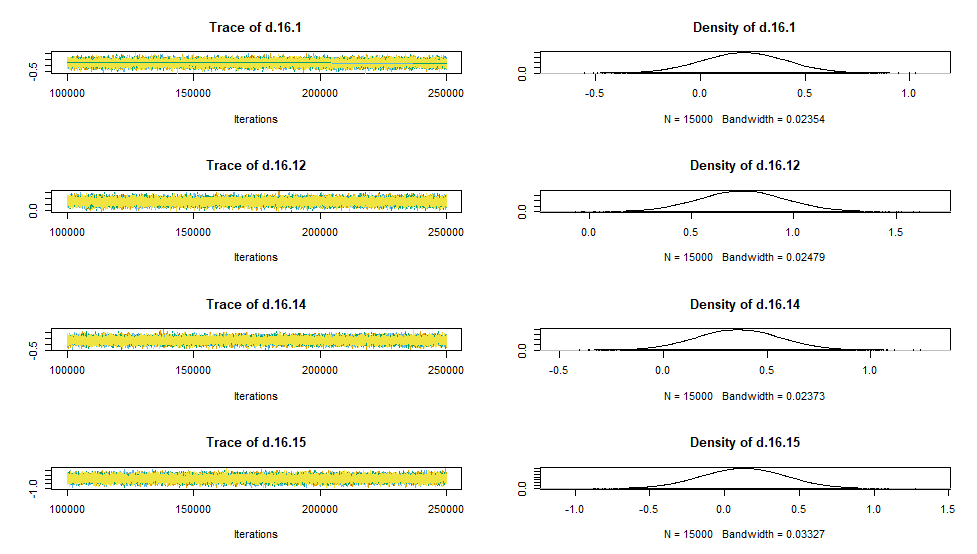

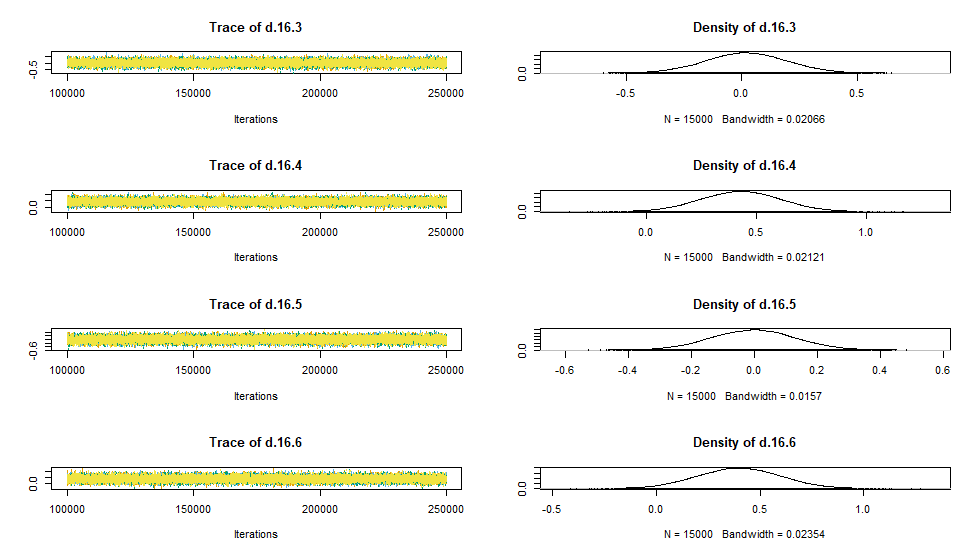


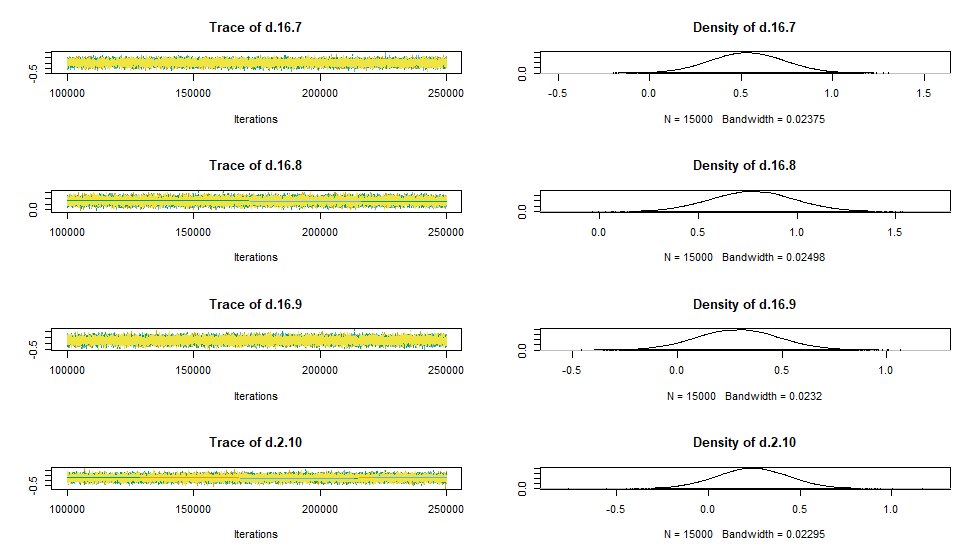

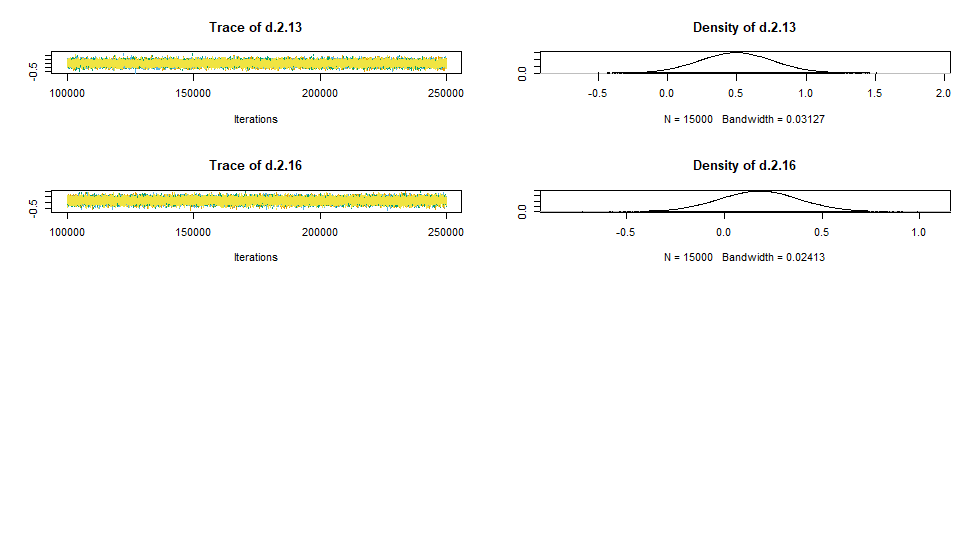


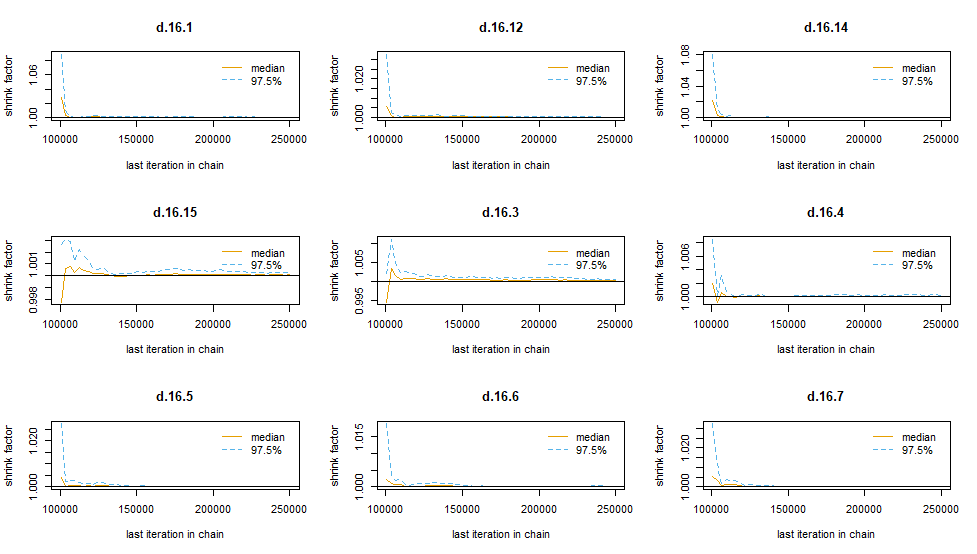

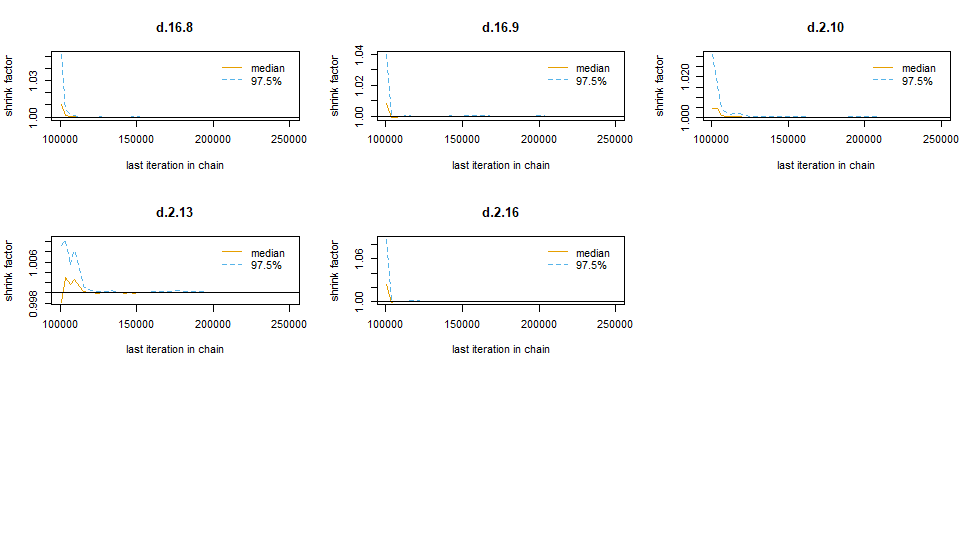


1. AEs


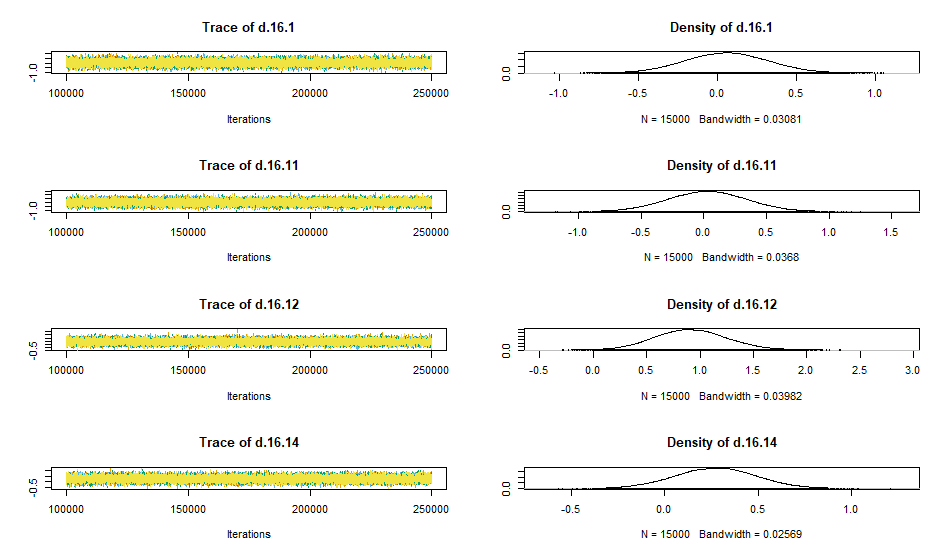

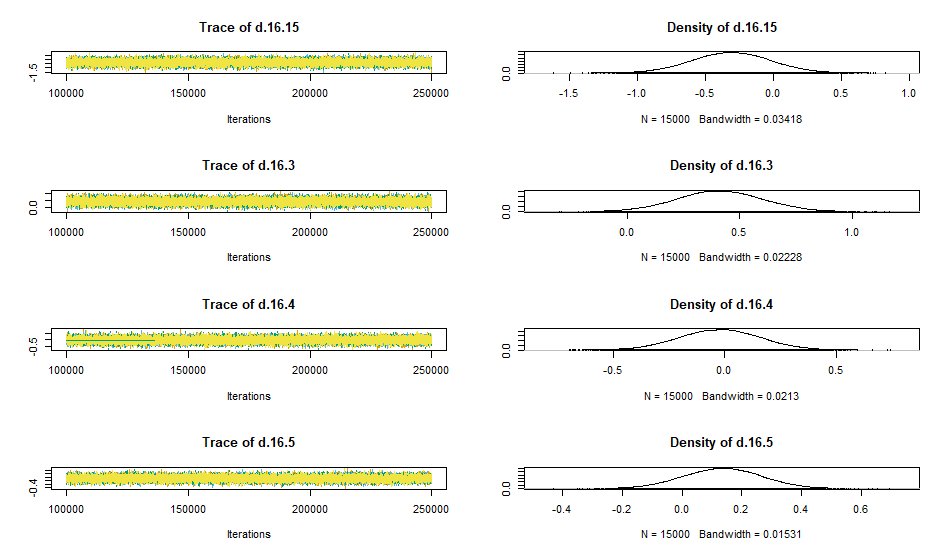


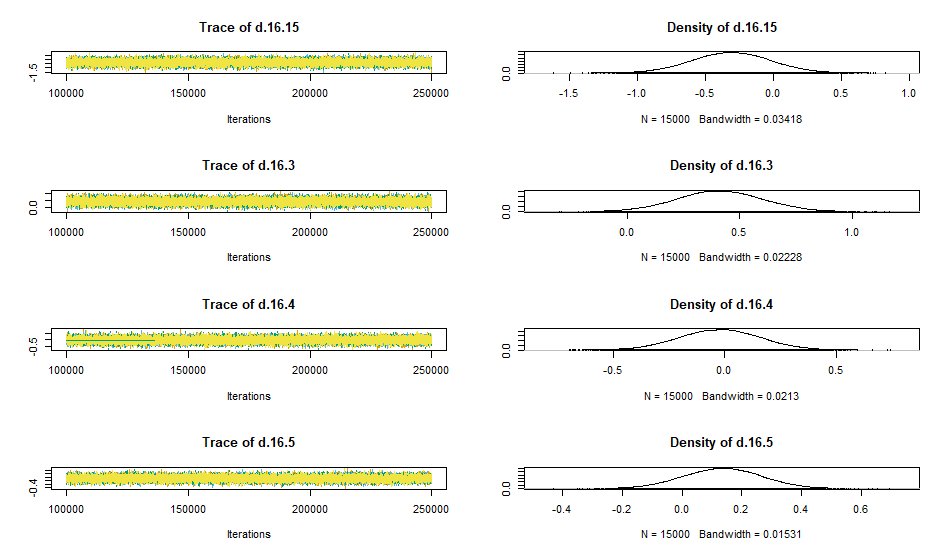

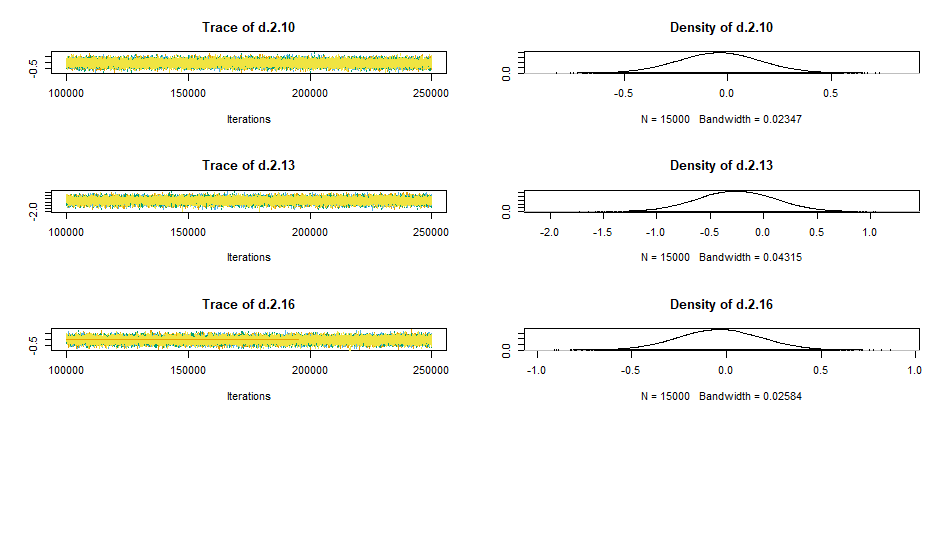


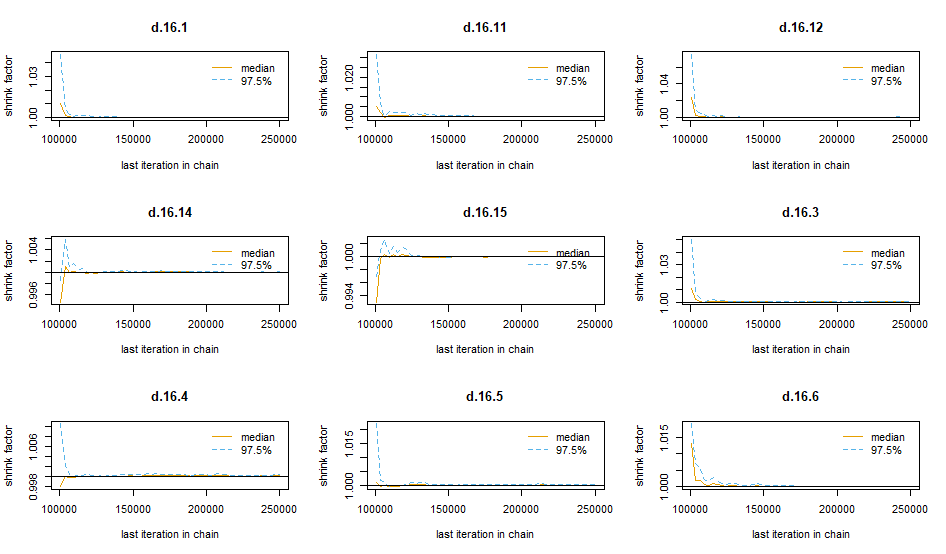

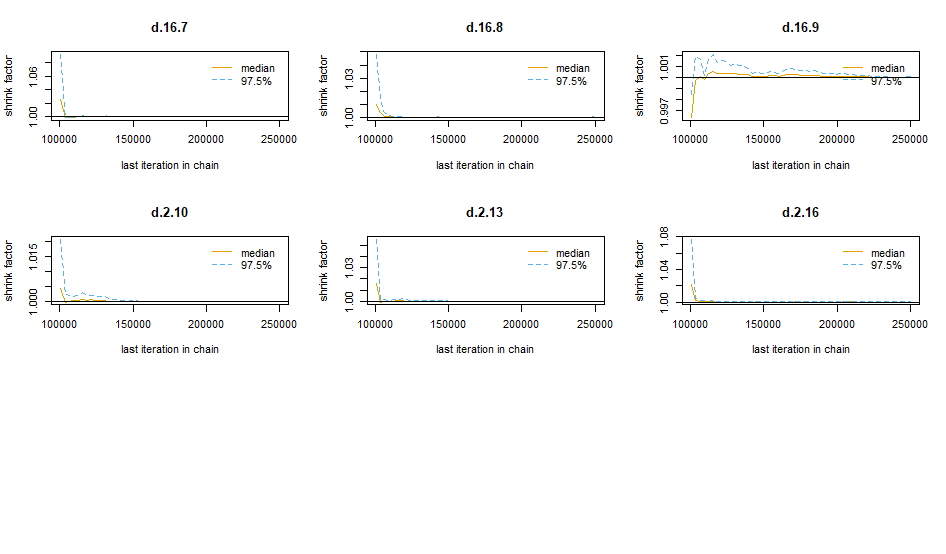


Convergence of the four chains established by inspection of the Brooks-Gelman-Rubin diagnostic and the density trace plot: (A) overall survival (B) progression-free survival, (C) objective response rate, (D) adverse events of grade 3 or higher. 1. Ade + chemo; 2. Ate + chemo; 3. Dur + Tre + chemo; 4. Dur + chemo; 5. Ipi + chemo; 6. Pem + chemo; 7. Ser + chemo; 8. Ben + Anl + chemo; 9. Tis + chemo; 10. Tir + Ate + chemo; 11. Tor + chemo; 12. Anl + chemo; 13. Bev + Ate + chemo; 14. Soc + chemo; 15. Bev + chemo; 16. Chemo; Ipi + chemo, ipilimumab + chemotherapy; Pem + chemo, pembrolizumab + chemotherapy; Dur + Tre + chemo, durvalumab + tremelimuamb + chemotherapy; Dur + chemo, durvalumab + chemotherapy; Ade + chemo, adebrelimab + chemotherapy; Ate + chemo, atezolizumab + chemotherapy; Ser + chemo, serplulimab + chemotherapy; Tir + Ate + chemo, tiragolumab + atezolizumab + chemotherapy; Ben + Anl + chemo, benmelstobart + anlotinib + chemotherapy; Anl + chemo, anlotinib + chemotherapy; Tis + chemo, tislelizumab + chemotherapy; Tor + chemo, toripalimab + chemotherapy; Bev + Ate + chemo, bevacizumab + atezolizumab + chemotherapy; Bev + chemo, bevacizumab + chemotherapy; Soc + chemo, socazolimab + chemotherapy;

Figure S3 The rank-heat plot presented in this study illustrates the evaluation of several first-line treatment regimens for patients with extensive-stage small cell lung cancer (ES-SCLC). (A) OS; (B) PFS; OS, overall survival; PFS, progression-free survival; ADE, adebrelimab; ATE, atezolizumab; DUR + TRE, durvalumab + tremelimuamb; DUR, durvalumab; IPI, ipilimumab; PEM, pembrolizumab; SER, serplulimab; BEN + ANL, benmelstobart + anlotinib; TIS, tislelizumab; TIR + ATE, tiragolumab + atezolizumab; TOR, toripalimab; ANL, anlotinib; BEV+ ATE, bevacizumab + atezolizumab; SOC, socazolimab; BEV, bevacizumab; CHEMO, chemotherapy.


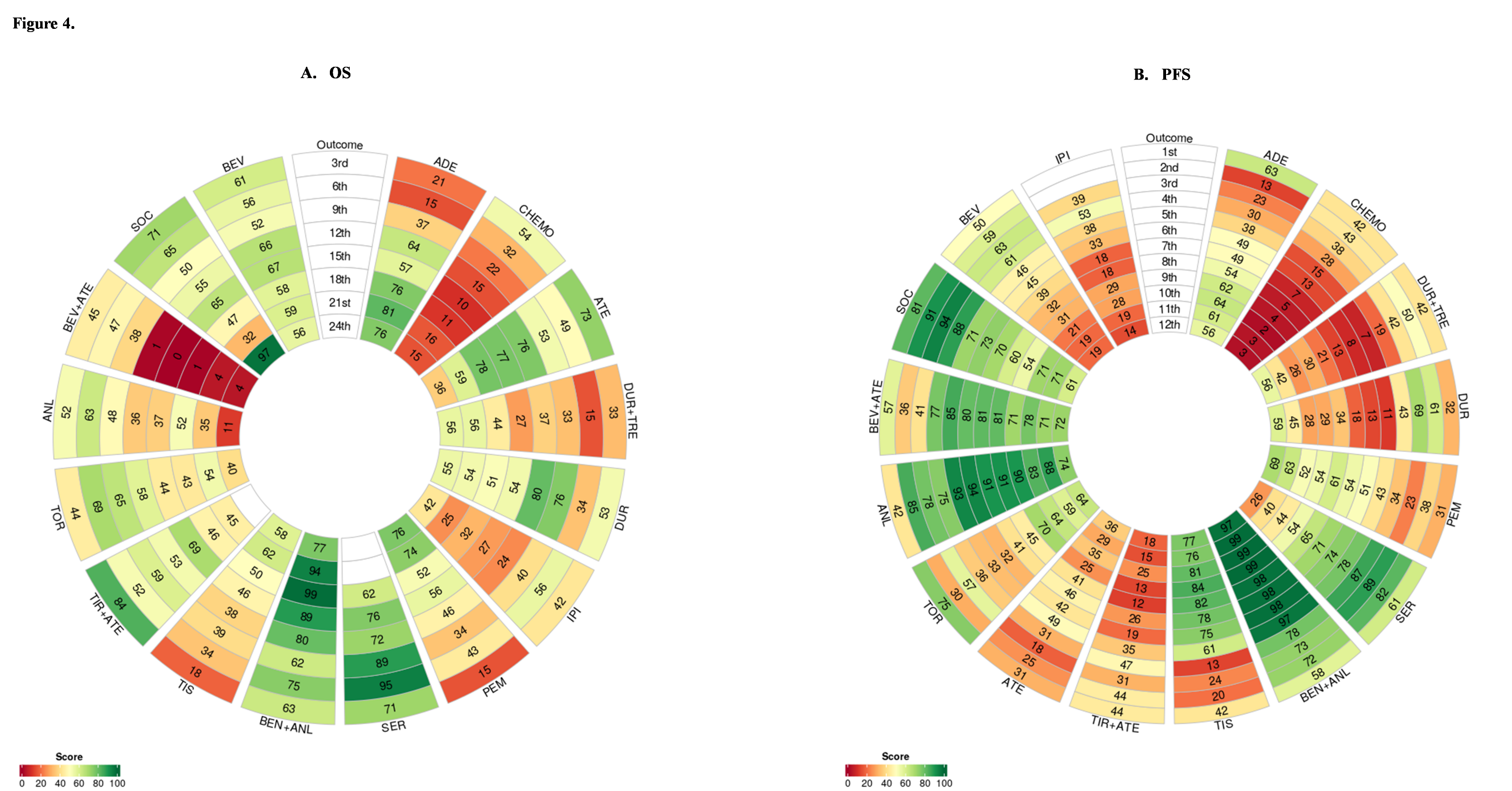


Figure S4 Efficacy and Safety summaries from Bayesian Network Meta-Analysis in SCLC patients. (A) Pooled HRs and 95%CI in patients aged＜65; (B) B. Pooled HRs and 95%CI in patients aged≥65; (C) Pooled HRs and 95%CI in female patients; (D) Pooled HRs and 95%CI in male patients; (E) Pooled HRs and 95%CI in patients with no brain metastasis; The important results are highlighted in red and bold. HRs, Hazard ratios; CI, confidence interval; y, years; Ade + chemo, adebrelimab + chemotherapy; Ate + chemo, atezolizumab + chemotherapy; Dur + Tre + chemo, durvalumab + tremelimuamb + chemotherapy; Dur + chemo, durvalumab + chemotherapy; Ipi + chemo, ipilimumab + chemotherapy; Pem + chemo, pembrolizumab + chemotherapy; Ser + chemo, serplulimab + chemotherapy; Ben + Anl + chemo, benmelstobart + anlotinib + chemotherapy; Tis + chemo, tislelizumab + chemotherapy; Tir + Ate + chemo, tiragolumab + atezolizumab + chemotherapy; Tor + chemo, toripalimab + chemotherapy; Anl + chemo, anlotinib + chemotherapy; Bev + Ate + chemo, bevacizumab + atezolizumab + chemotherapy; Soc + chemo, socazolimab + chemotherapy; Bev + chemo, bevacizumab + chemotherapy; Chemo, chemotherapy.


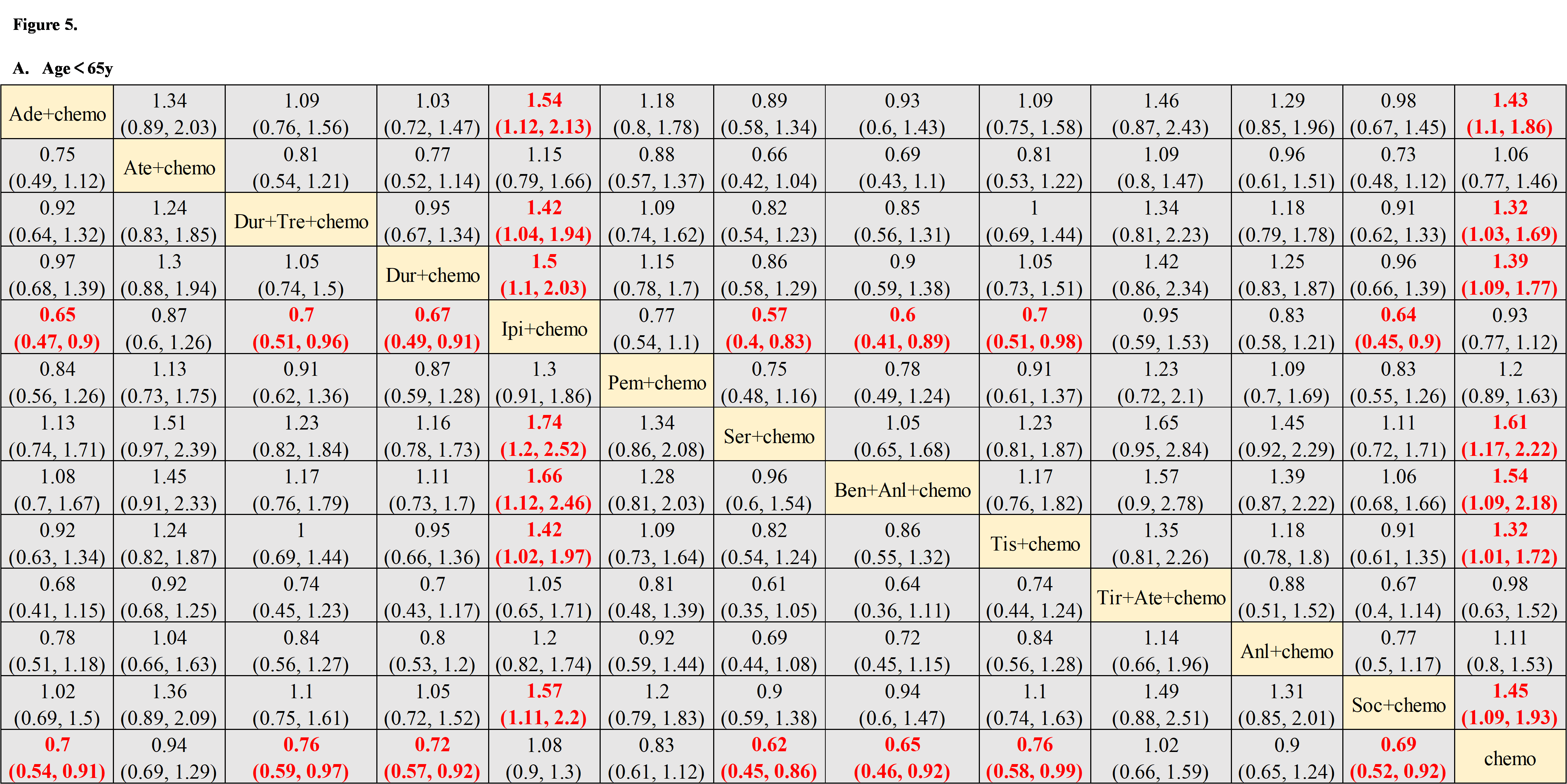


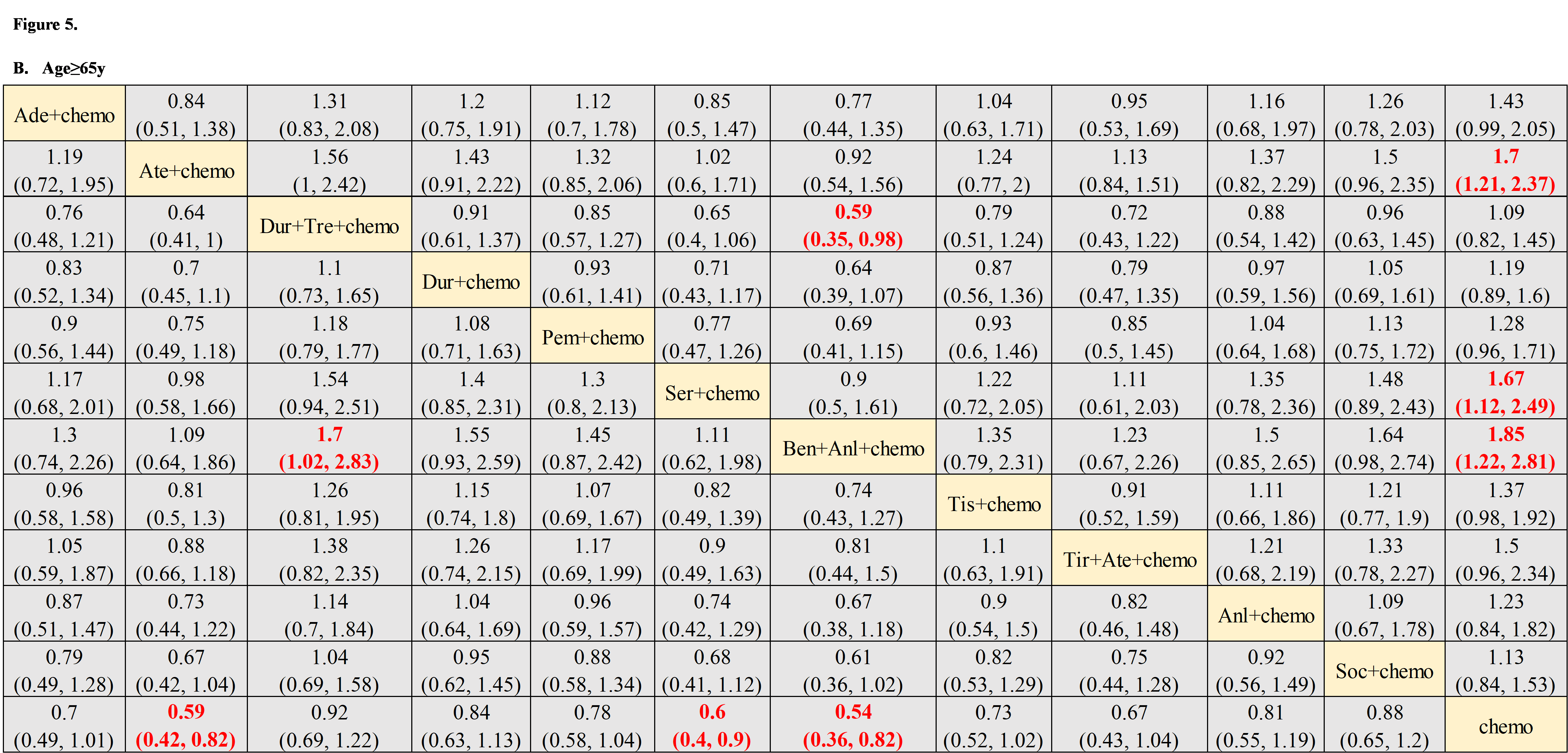


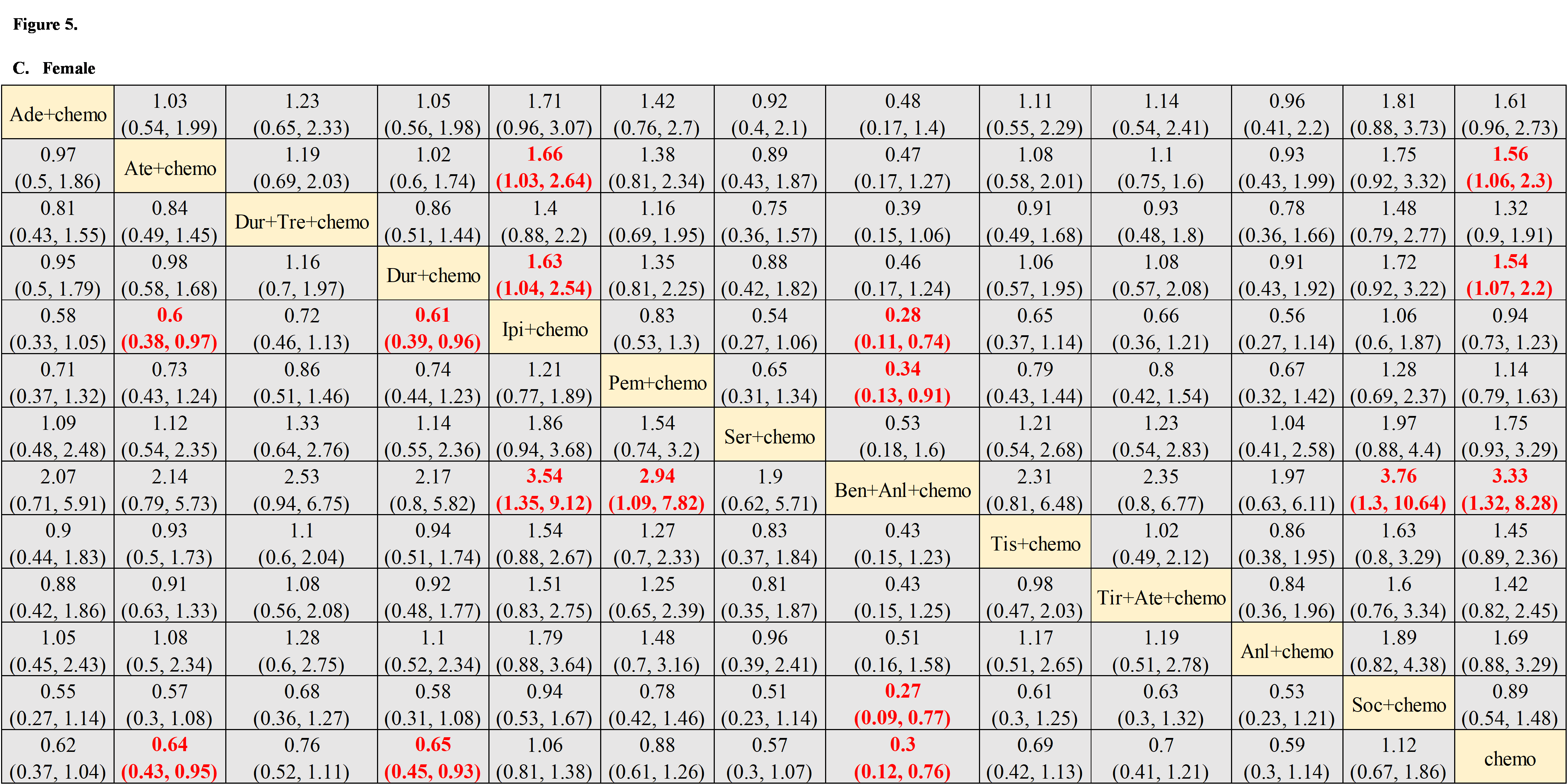

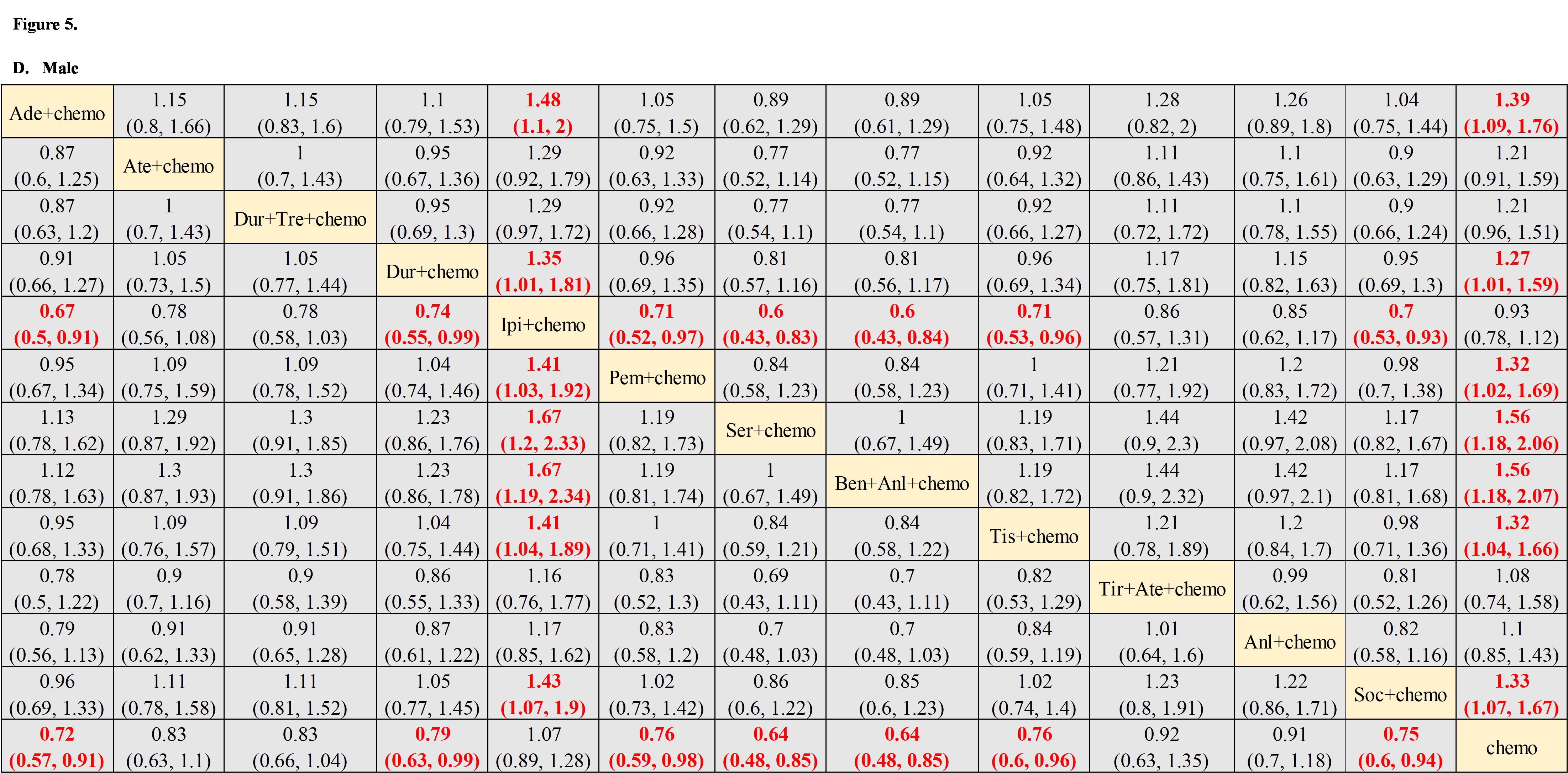

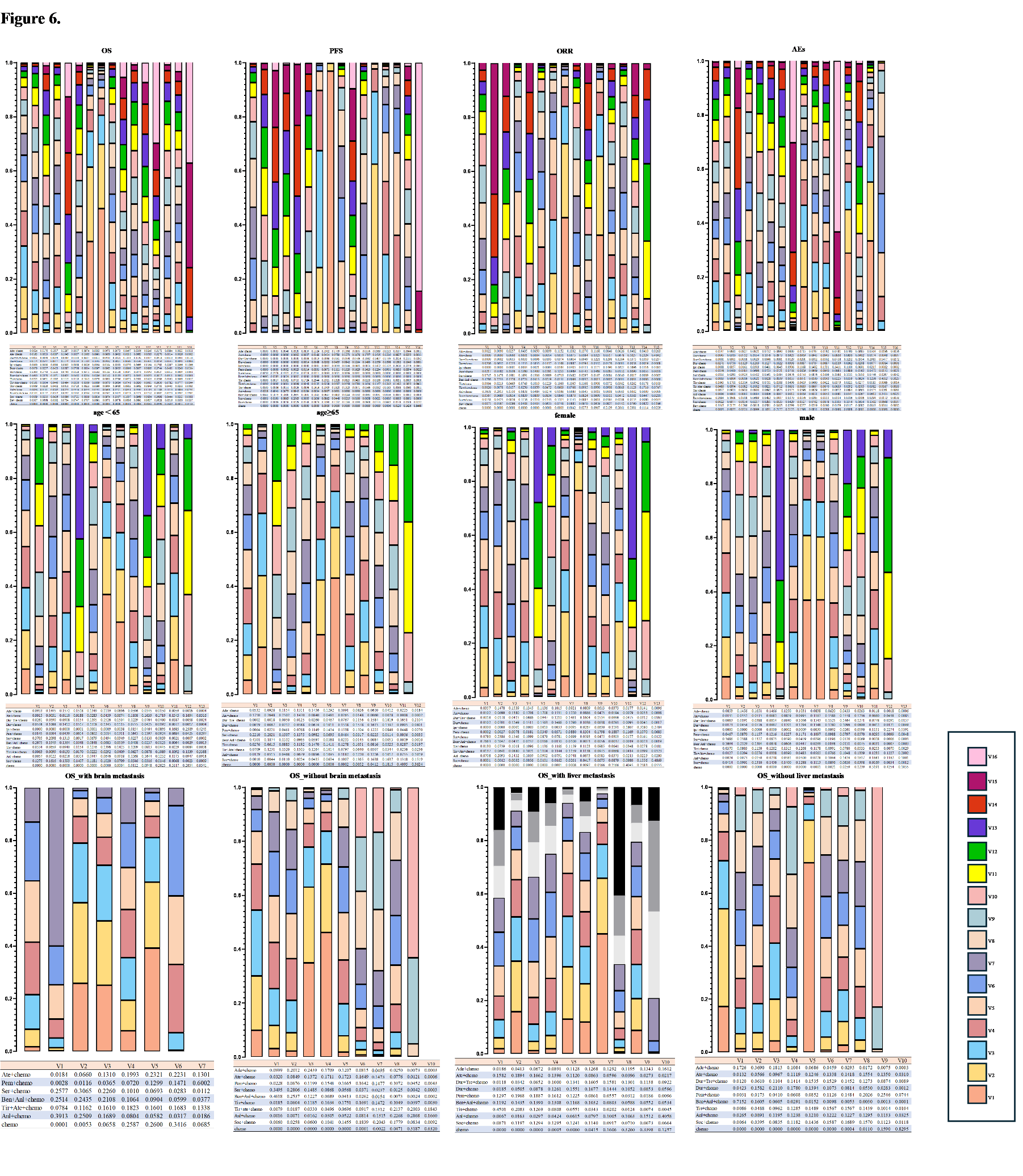
Figure S5 Bayesian ranking profiles indicate the likelihood of causing fewer grade ≥3 adverse events (AEs) or the effectiveness of immunotherapies, ranked from most likely to least likely in the overall population. The top illustrates the possibility of improving overall survival (OS), extending progression-free survival (PFS), or being the most effective at causing fewer grade ≥3 adverse events (AEs). Ade + chemo, adebrelimab + chemotherapy; Ate + chemo, atezolizumab + chemotherapy; Dur + Tre + chemo, durvalumab + tremelimuamb + chemotherapy; Dur + chemo, durvalumab + chemotherapy; Ipi + chemo, ipilimumab + chemotherapy; Pem + chemo, pembrolizumab + chemotherapy; Ser + chemo, serplulimab + chemotherapy; Ben + Anl + chemo, benmelstobart + anlotinib + chemotherapy; Tis + chemo, tislelizumab + chemotherapy; Tir + Ate + chemo, tiragolumab + atezolizumab + chemotherapy; Tor + chemo, toripalimab + chemotherapy; Anl + chemo, anlotinib + chemotherapy; Bev + Ate + chemo, bevacizumab + atezolizumab + chemotherapy; Soc + chemo, socazolimab + chemotherapy; Bev + chemo, bevacizumab + chemotherapy; Chemo, chemotherapy.

Figure S6 The Brooks-Gelman-Rubin diagnostic and the density trace plot in group meta-analysis.

1. OS


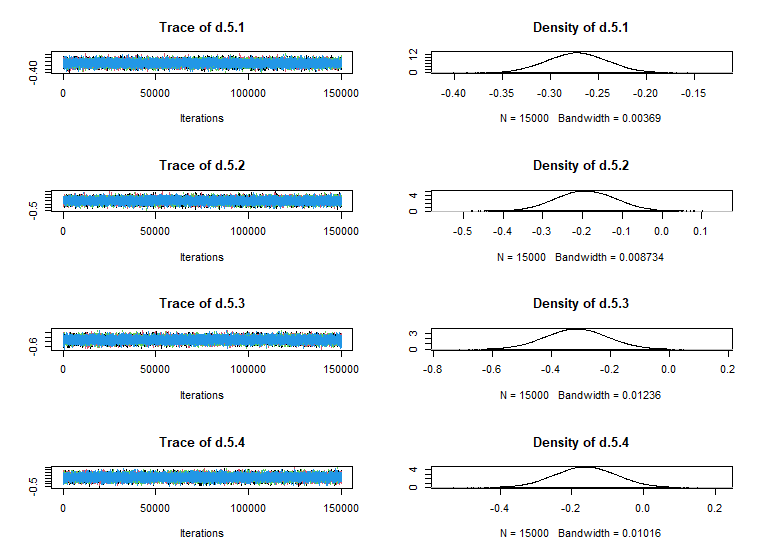

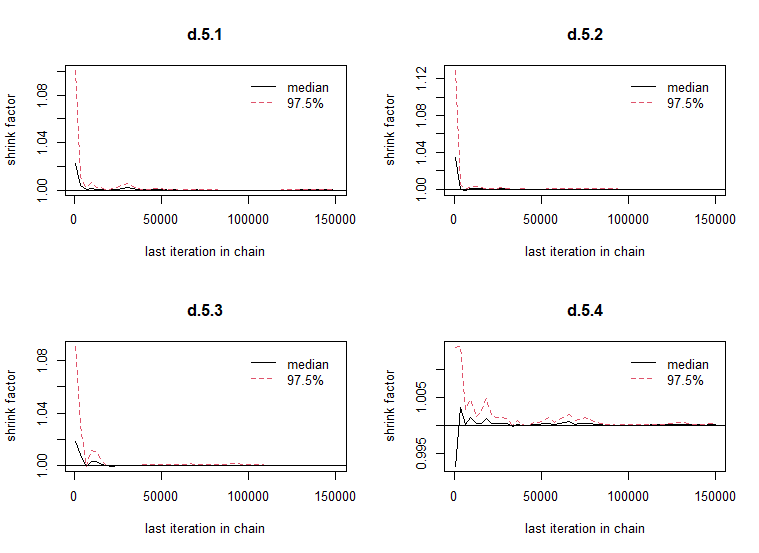


1. PFS


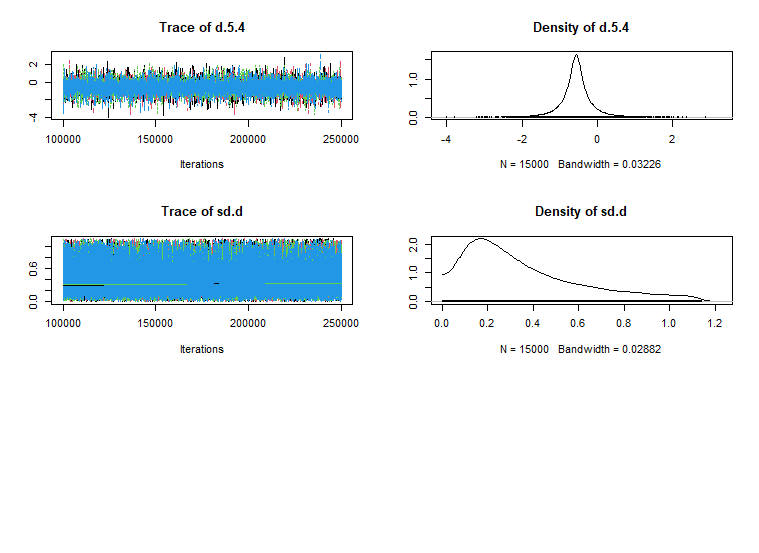

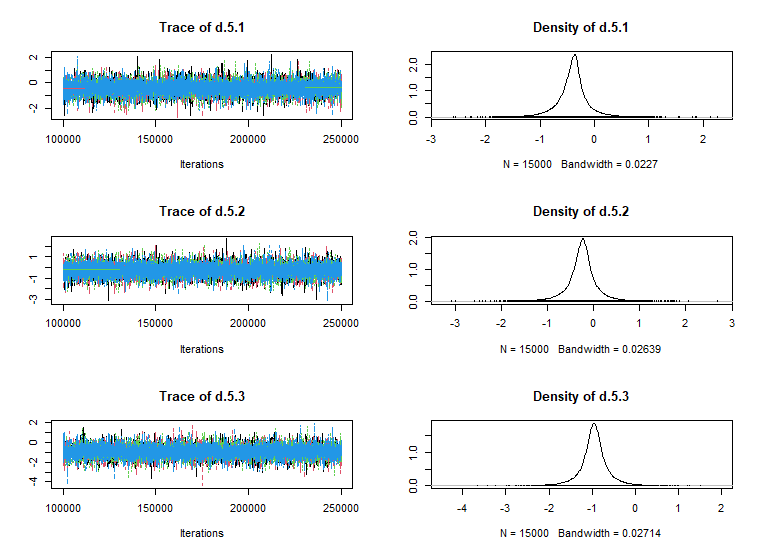

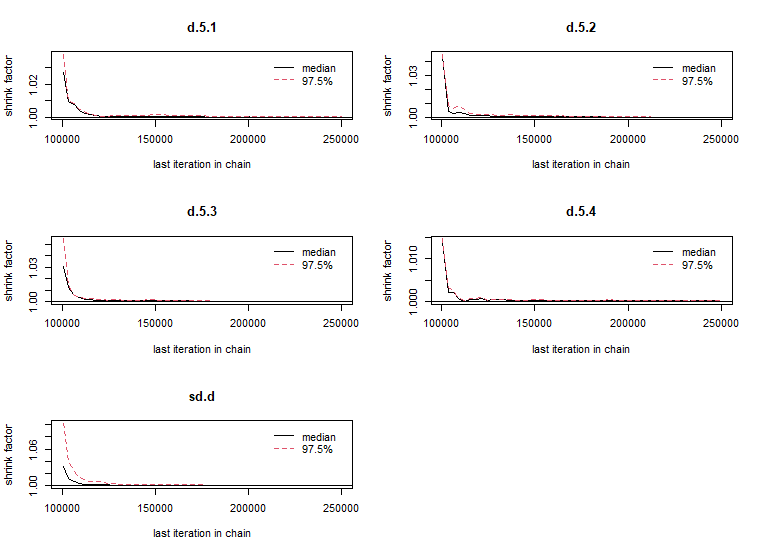


C．ORR


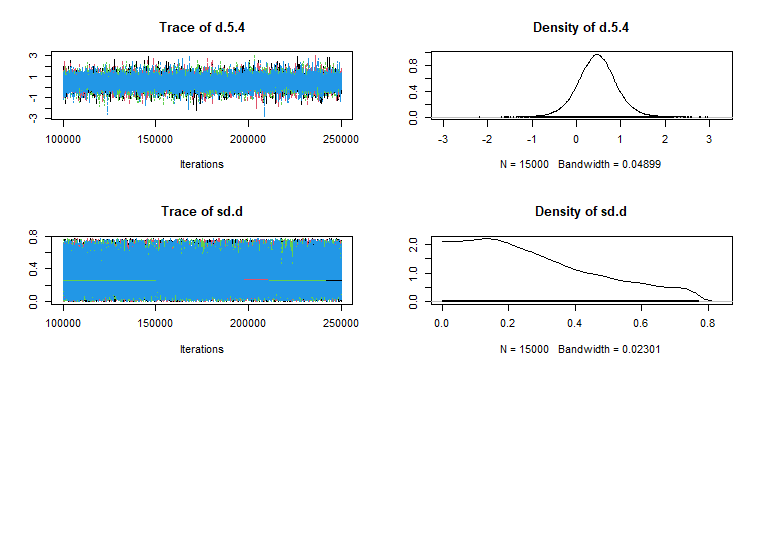

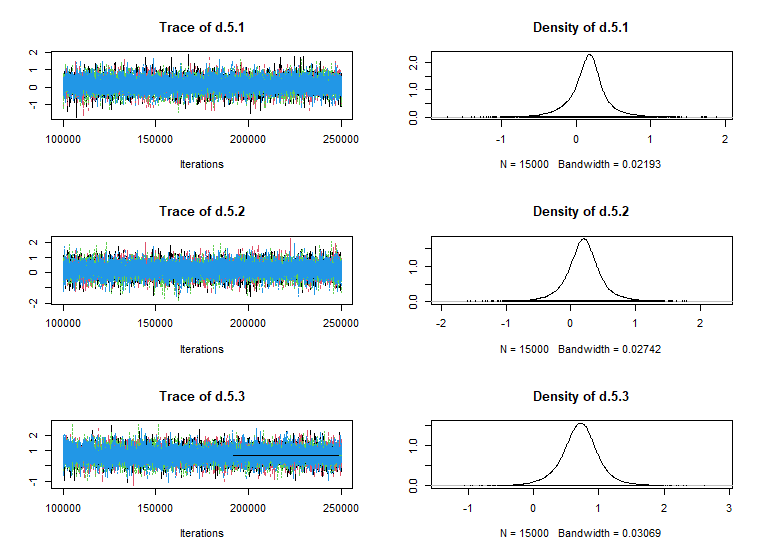

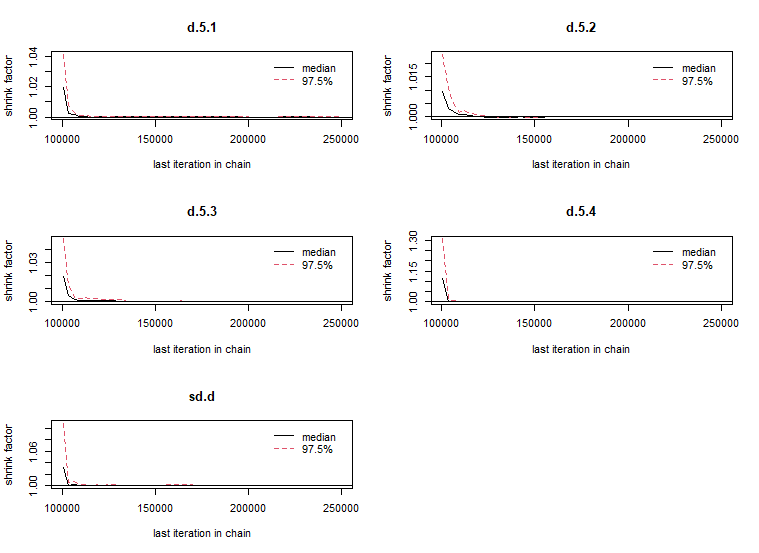


D．AEs


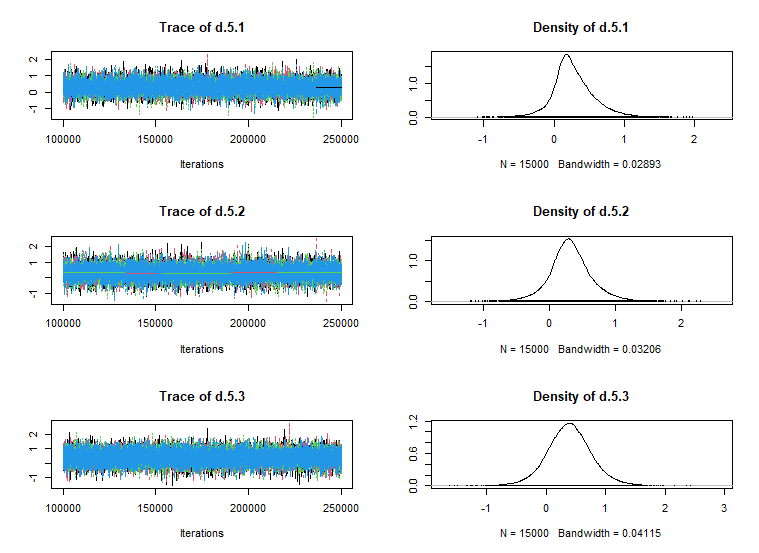

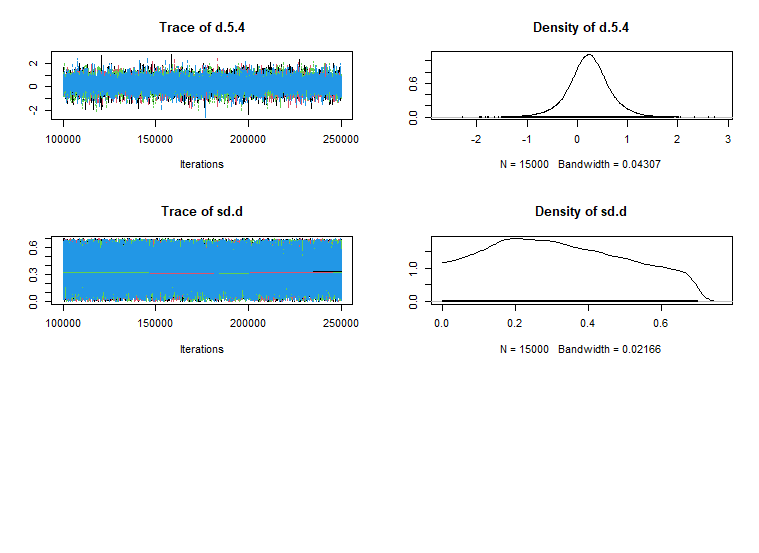


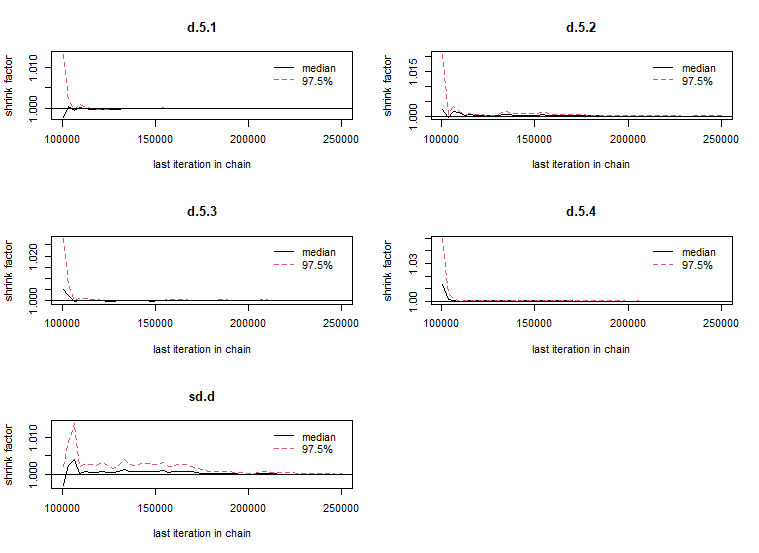


Convergence of the four chains established by inspection of the Brooks-Gelman-Rubin diagnostic and the density trace plot: (A) overall survival (B) progression-free survival, (C) objective response rate, (D) adverse events of grade 3 or higher. 1. ICI + Chemo; 2. ICI + ICI + Chemo; 3. ICI + Antiangio +.Chemo; 4. Antiangio + Chemo; 5. Chemo; ICI, Immune checkpoint inhibitors; Antiangio, Antiangiogenic agent; Chemo, Chemotherapy.

Figure S7 Forest plot of survival outcomes from integrated analysis of different therapy strategies in SCLC patients. (A) OS; (B) PFS; (C) ORR; (D) AEs; OS, overall survival; PFS, progression-free survival; ORR, objective response rate; AE, Adverse event; HR, Hazard ratio; CI, confidence interval; ICI, immune checkpoint inhibitor; ICI + Chemo, ICI + Chemotherapy; Antiangio + Chemo, Antiangiogenic agent + Chemotherapy; ICI + ICI + Chemo, ICI + ICI + Chemotherapy; ICI + Antiangio + Chemo, ICI + Antiangiogenic agent + Chemotherapy; Chemo, Chemotherapy.

A．OS


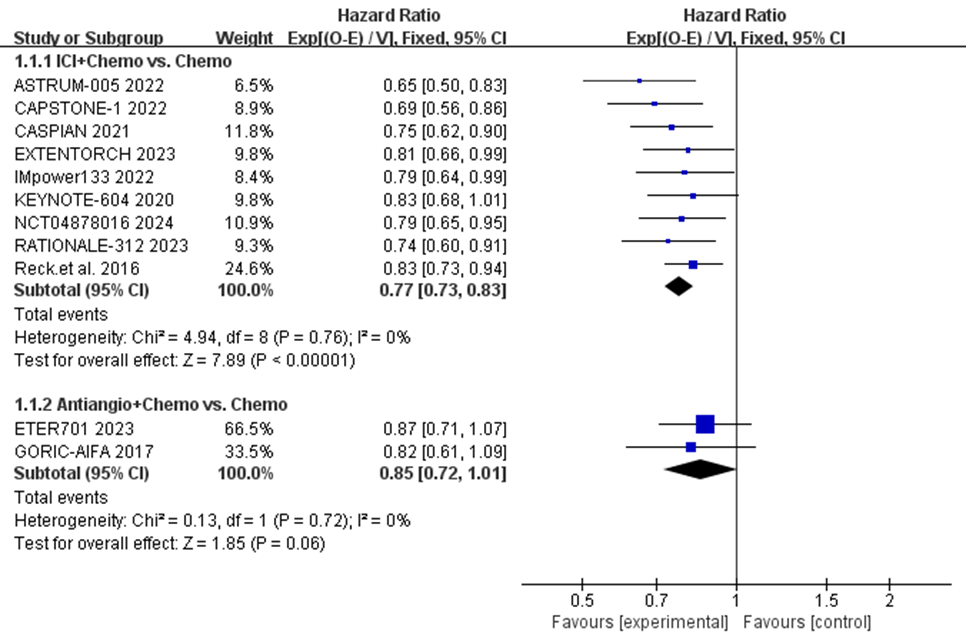


B．PFS


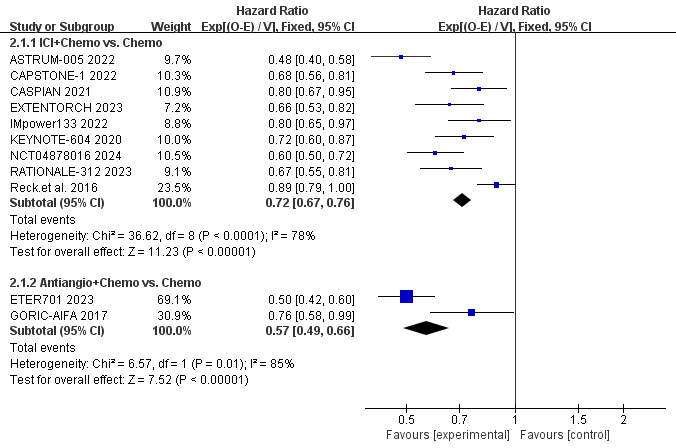


C．ORR


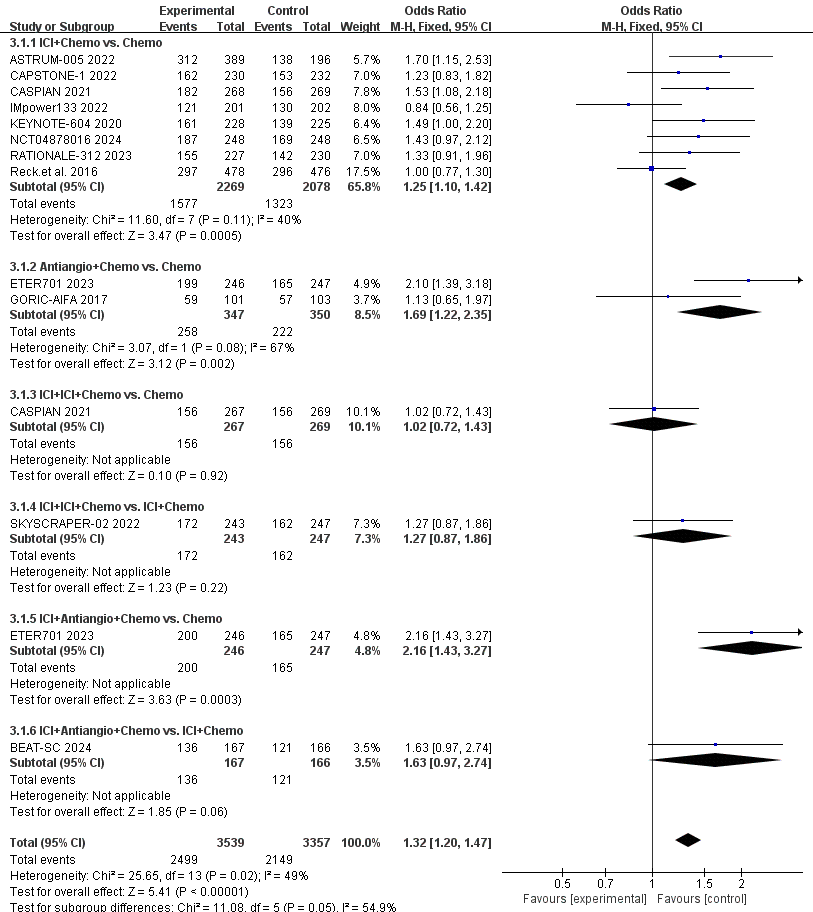


D．AEs


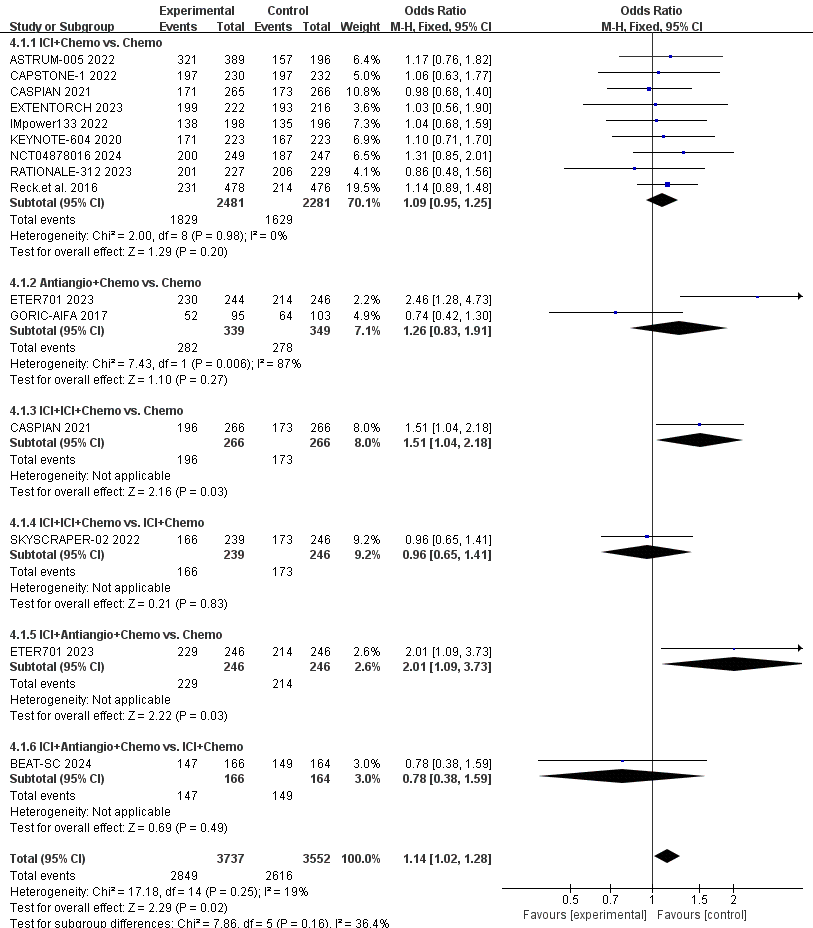


Figure S8 Network meta-analysis of comparisons on each outcome in various treatment strategies of ES-SCLC patients. Each circle represents a treatment, and the line between the two points represents a comparison between the two treatment strategies. The numbers represent the count of involved studies, with the thickness of the lines proportional to the number of studies included. ICI, immune checkpoint inhibitor; ICI + Chemo, ICI + Chemotherapy; Antiangio + Chemo, Antiangiogenic agent + Chemotherapy; ICI + ICI + Chemo, ICI + ICI + Chemotherapy; ICI + Antiangio + Chemo, ICI + Antiangiogenic agent + Chemotherapy; Chemo, Chemotherapy.


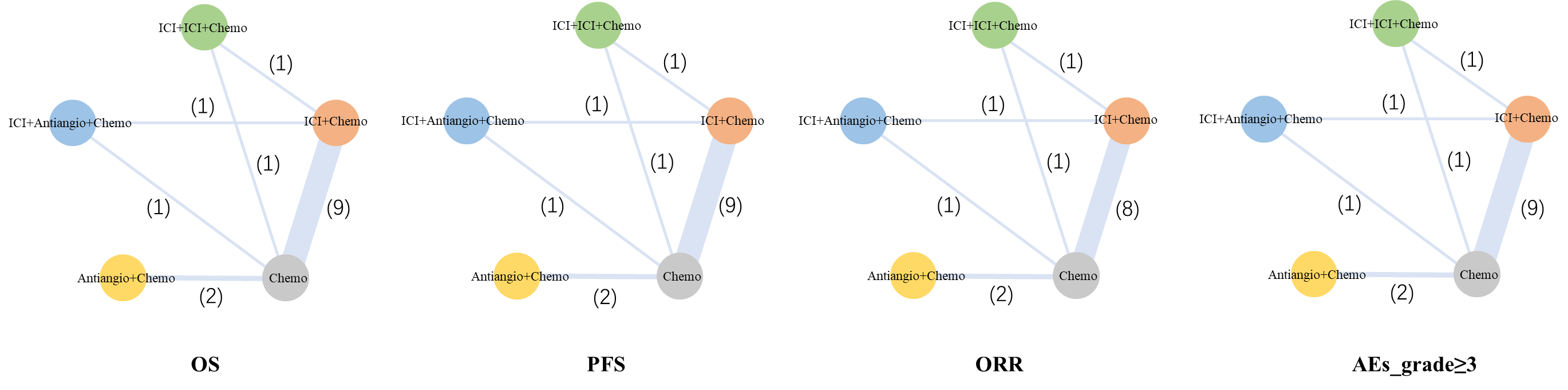


Figure S9 Efficacy and Safety summaries from Bayesian Network Meta-Analysis of different treatment strategies in SCLC patients. ICI, immune checkpoint inhibitor; ICI + Chemo, ICI + Chemotherapy; Antiangio + Chemo, Antiangiogenic agent + Chemotherapy; ICI + ICI + Chemo, ICI + ICI + Chemotherapy; ICI + Antiangio + Chemo, ICI + Antiangiogenic agent + Chemotherapy; Chemo, Chemotherapy.

A．OS

B．PFS

C．ORR

D．AEs

Figure S10 Bayesian ranking profiles indicate the likelihood of causing fewer grade ≥3 adverse events (AEs) or the effectiveness of treatment strategies, ranked from most likely to least likely in the overall population. The top illustrates the possibility of improving overall survival (OS), extending progression-free survival (PFS), or being the most effective at causing fewer grade ≥3 adverse events (AEs). PFS, progression-free survival; OS, overall survival; ICI, immune checkpoint inhibitor; ICI + Chemo, ICI + Chemotherapy; Antiangio + Chemo, Antiangiogenic agent + Chemotherapy; ICI + ICI + Chemo, ICI + ICI + Chemotherapy; ICI + Antiangio + Chemo, ICI + Antiangiogenic agent + Chemotherapy; Chemo, Chemotherapy.


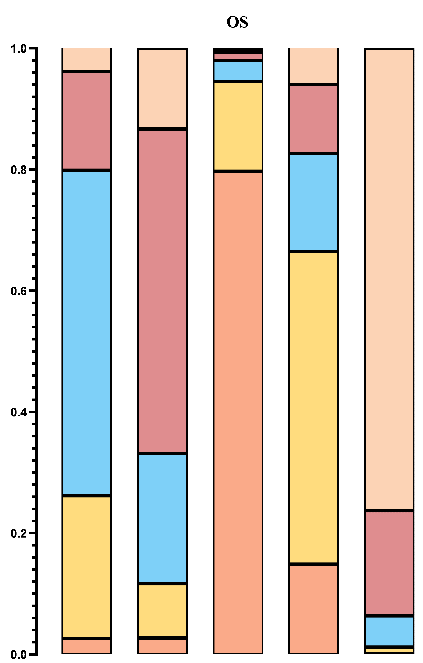

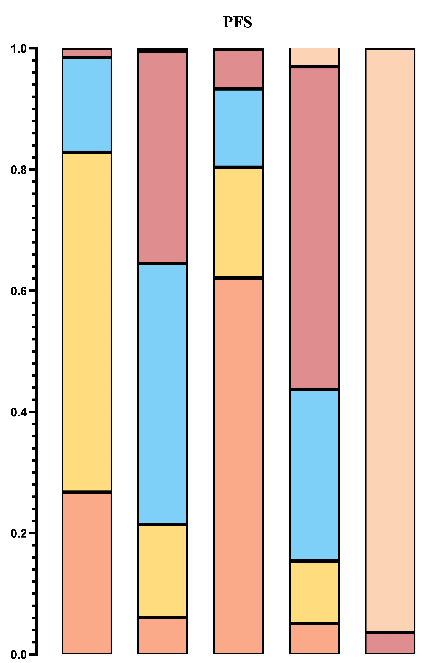

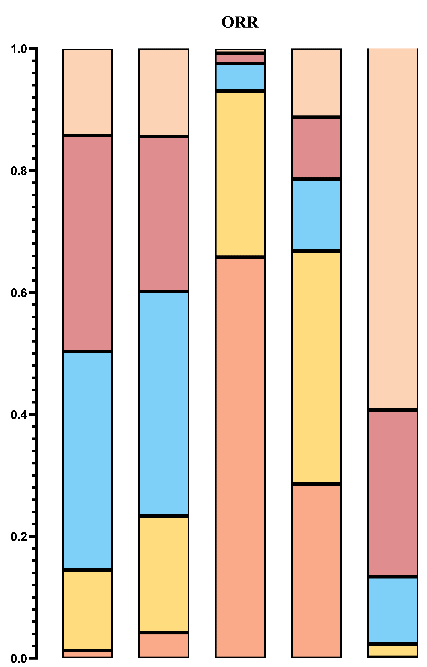

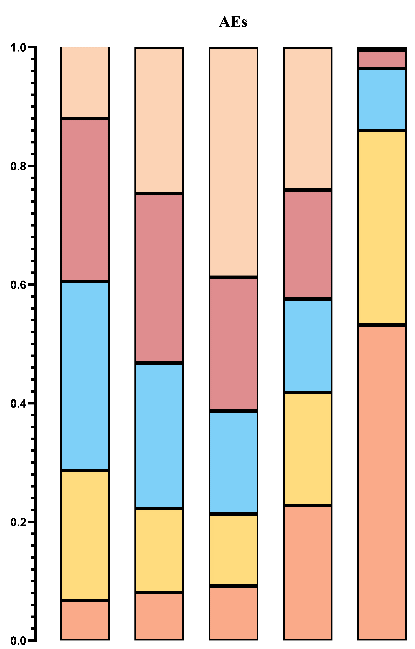

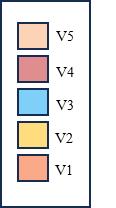

**Figure S11** The Brooks-Gelman-Rubin diagnostic and the density trace plot in group meta-analysis. (Excluding ipilimumab + chemotherapy).

1. OS


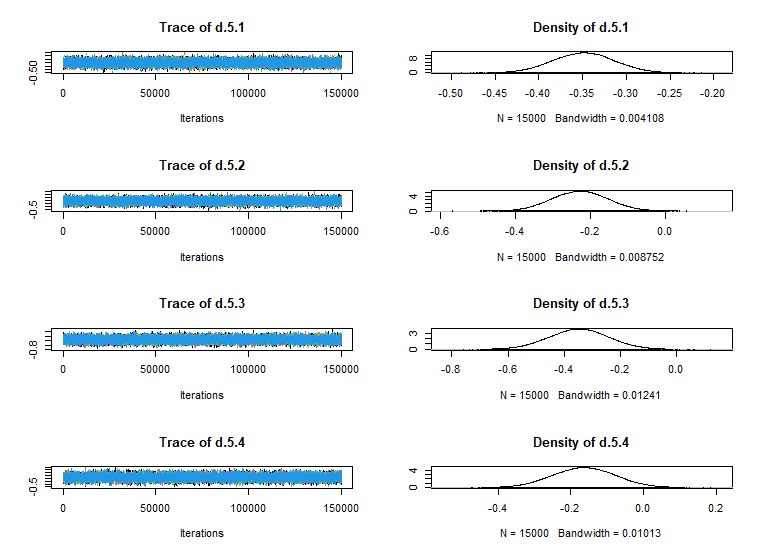

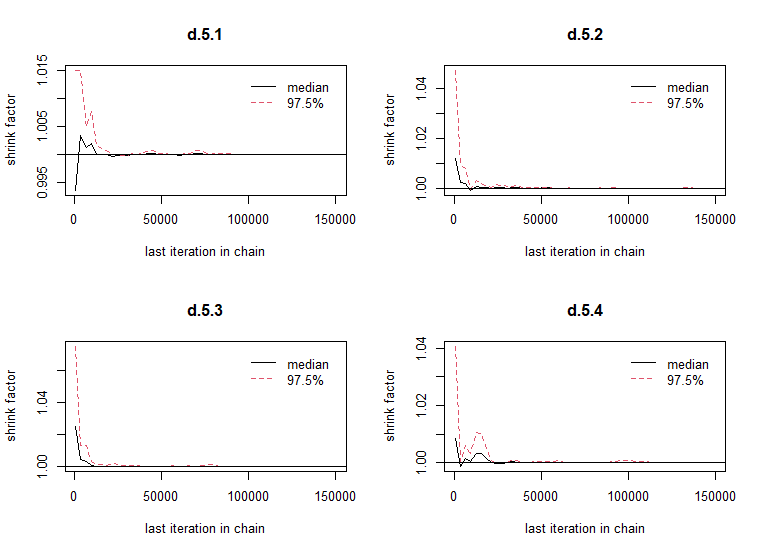


1. PFS


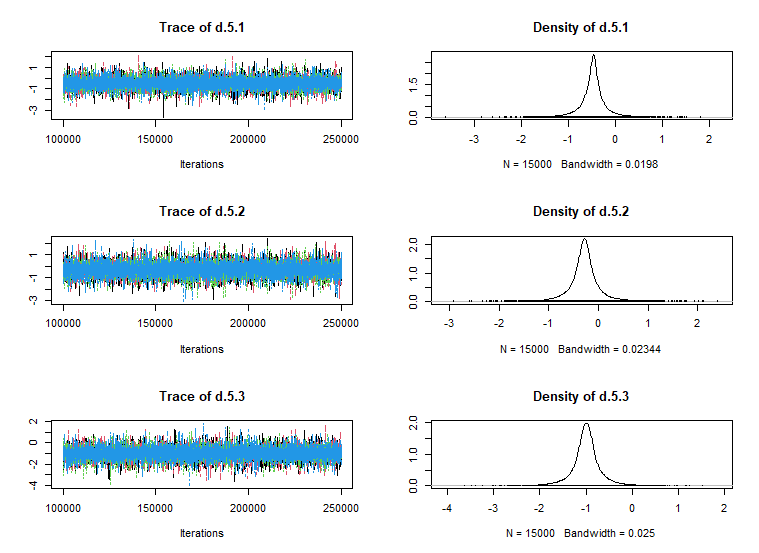

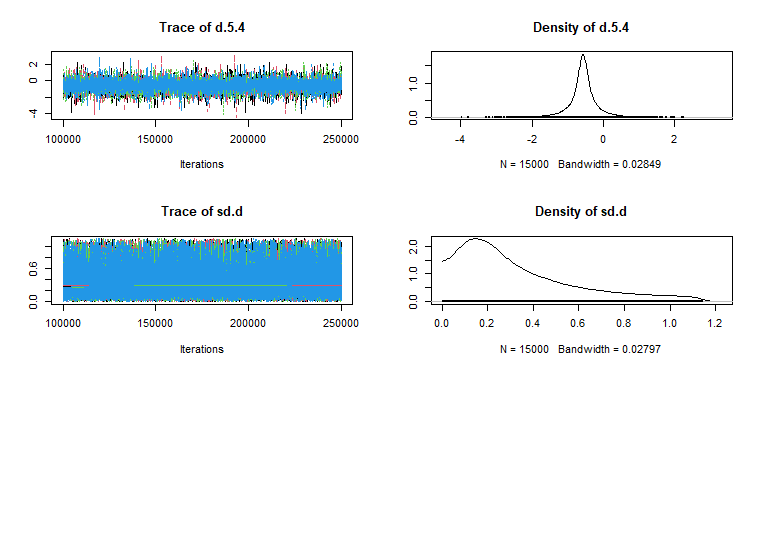


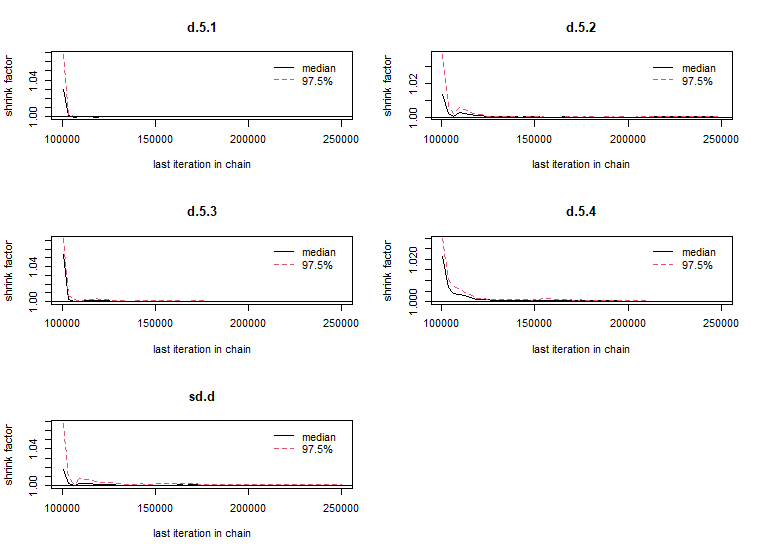


1. ORR


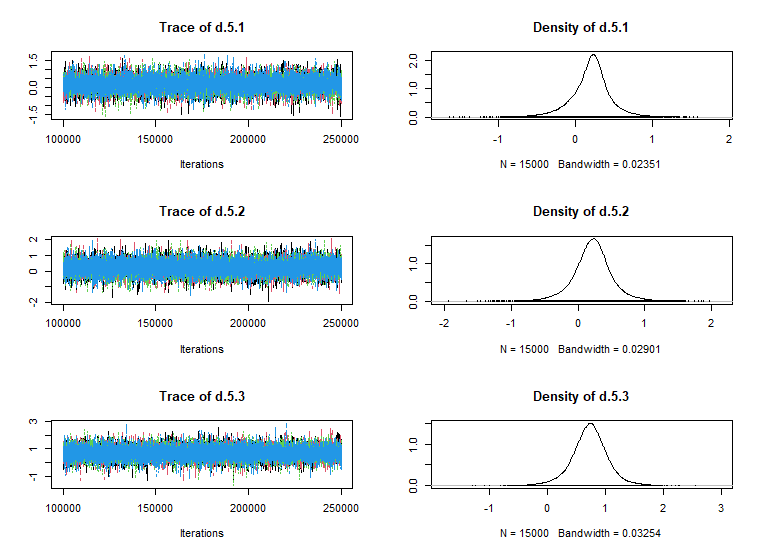

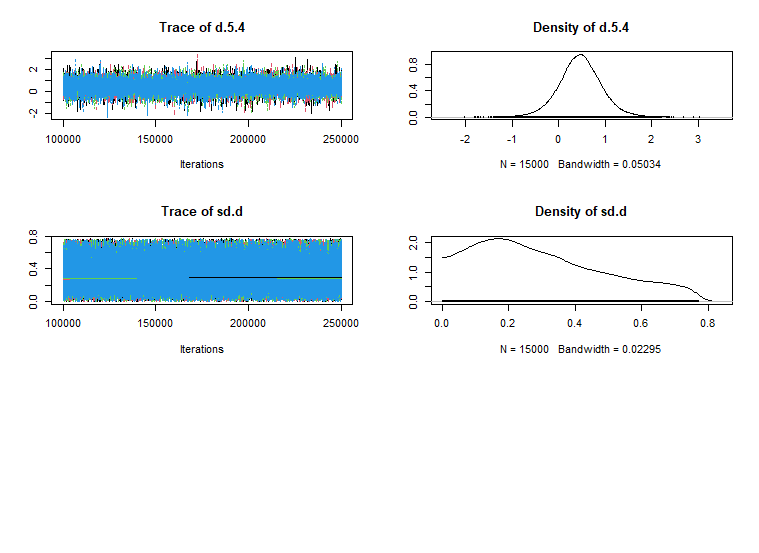

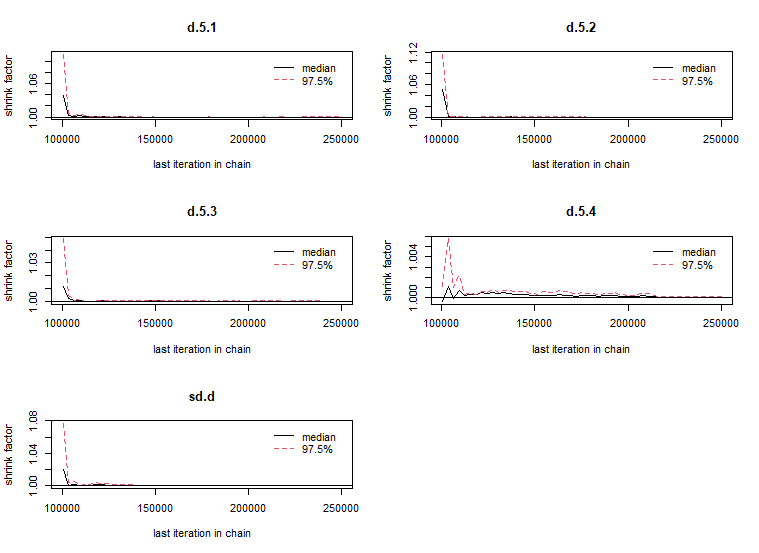


1. AEs


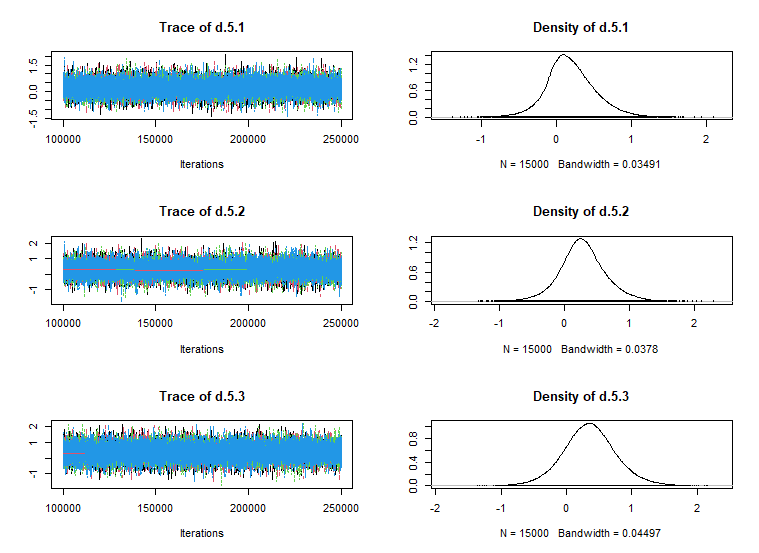

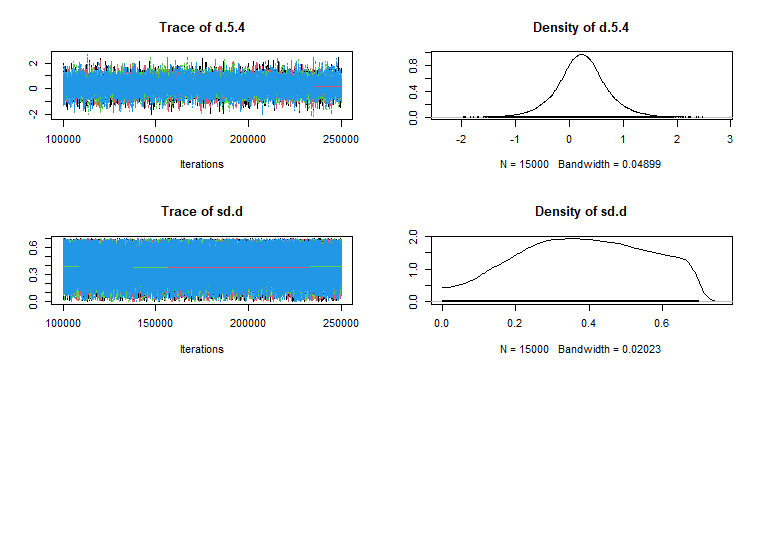

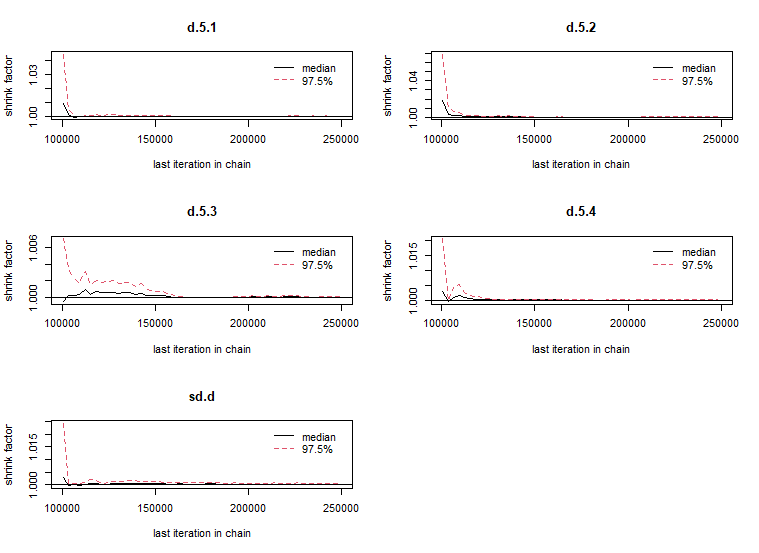


Convergence of the four chains established by inspection of the Brooks-Gelman-Rubin diagnostic and the density trace plot: (A) overall survival (B) progression-free survival, (C) objective response rate, (D) adverse events of grade 3 or higher. 1. ICI + Chemo (Excluding ipilimumab + chemotherapy); 2. ICI + ICI + Chemo; 3. ICI + Antiangio + Chemo; 4. Antiangio + Chemo; 5. Chemo; ICI, Immune checkpoint inhibitors; Antiangio, Antiangiogenic agent; Chemo, Chemotherapy.
